# Rapid iPSC inclusionopathy models shed light on formation, consequence, and molecular subtype of α-synuclein inclusions

**DOI:** 10.1016/j.neuron.2024.06.002

**Published:** 2024-07-29

**Authors:** Isabel Lam, Alain Ndayisaba, Amanda J. Lewis, YuHong Fu, Giselle T. Sagredo, Anastasia Kuzkina, Ludovica Zaccagnini, Meral Celikag, Jackson Sandoe, Ricardo L. Sanz, Aazam Vahdatshoar, Timothy D. Martin, Nader Morshed, Toru Ichihashi, Arati Tripathi, Nagendran Ramalingam, Charlotte Oettgen-Suazo, Theresa Bartels, Manel Boussouf, Max Schäbinger, Erinc Hallacli, Xin Jiang, Amrita Verma, Challana Tea, Zichen Wang, Hiroyuki Hakozaki, Xiao Yu, Kelly Hyles, Chansaem Park, Xinyuan Wang, Thorold W. Theunissen, Haoyi Wang, Rudolf Jaenisch, Susan Lindquist, Beth Stevens, Nadia Stefanova, Gregor Wenning, Wilma D.J. van de Berg, Kelvin C. Luk, Rosario Sanchez-Pernaute, Juan Carlos Gómez-Esteban, Daniel Felsky, Yasujiro Kiyota, Nidhi Sahni, S. Stephen Yi, Chee Yeun Chung, Henning Stahlberg, Isidro Ferrer, Johannes Schöneberg, Stephen J. Elledge, Ulf Dettmer, Glenda M. Halliday, Tim Bartels, Vikram Khurana

**Affiliations:** 1 Ann Romney Center for Neurologic Diseases, Brigham and Women’s Hospital, Boston, MA, USA; 2 Division of Movement Disorders, American Parkinson Disease Association (APDA) Center for Advanced Research and MSA Center of Excellence, Department of Neurology, Brigham and Women’s Hospital, Boston, MA, USA; 3 Harvard Medical School, Boston, MA, USA; 4 Division of Neurobiology, Department of Neurology, Medical University of Innsbruck, Innsbruck, Austria; 5 École Polytechnique Fédérale de Lausanne and University of Lausanne, Lausanne, Switzerland; 6 The University of Sydney Brain and Mind Centre and Faculty of Medicine and Health School of Medical Science, Sydney, NSW, Australia; 7 Dementia Research Institute, University College London, London, UK; 8 Whitehead Institute for Biomedical Research, Cambridge, MA, USA; 9 Division of Genetics, Department of Medicine, Brigham and Women’s Hospital, Boston, MA, USA; 10 Howard Hughes Medical Institute, Chevy Chase, MD, USA; 11 Boston Children’s Hospital, Boston, MA, USA; 12 The Broad Institute of MIT and Harvard, Cambridge, MA, USA; 13 Nikon Corporation, Tokyo, Japan; 14 Yumanity Therapeutics, Cambridge, MA, USA; 15 University of California, San Diego, San Diego, CA, USA; 16 Amsterdam University Medical Center, Vrije Universiteit, Amsterdam, the Netherlands; 17 University of Pennsylvania Perelman School of Medicine, Philadelphia, PA, USA; 18 BioBizkaia Health Research Institute, Barakaldo, Spain; 19 Ikerbasque, Basque Foundation for Science, Bilbao, Spain; 20 Centre for Addiction and Mental Health, Toronto, ON, Canada; 21 University of Toronto, Toronto, ON, Canada; 22 The University of Texas MD Anderson Cancer Center, Houston, TX, USA; 23 Baylor College of Medicine, Houston, TX, USA; 24 The University of Texas at Austin, Austin, TX, USA; 25 The University of Barcelona, Institut d’Investigacio Biomedica de Bellvitge IDIBELL, Hospitalet de Llobregat, Barcelona, Spain; 26 Aligning Science Across Parkinson’s (ASAP) Collaborative Research Network, Chevy Chase, MD, USA; 27 Harvard Stem Cell Institute, Cambridge, MA, USA; 28 These authors contributed equally; 29 Deceased; 30 Lead contact

## Abstract

The heterogeneity of protein-rich inclusions and its significance in neurodegeneration is poorly understood. Standard patient-derived iPSC models develop inclusions neither reproducibly nor in a reasonable time frame. Here, we developed screenable iPSC “inclusionopathy” models utilizing piggyBac or targeted transgenes to rapidly induce CNS cells that express aggregation-prone proteins at brain-like levels. Inclusions and their effects on cell survival were trackable at single-inclusion resolution. Exemplar cortical neuron α-synuclein inclusionopathy models were engineered through transgenic expression of α-synuclein mutant forms or exogenous seeding with fibrils. We identified multiple inclusion classes, including neuroprotective p62-positive inclusions versus dynamic and neurotoxic lipid-rich inclusions, both identified in patient brains. Fusion events between these inclusion subtypes altered neuronal survival. Proteome-scale α-synuclein genetic- and physical-interaction screens pinpointed candidate RNA-processing and actin-cytoskeleton-modulator proteins like RhoA whose sequestration into inclusions could enhance toxicity. These tractable CNS models should prove useful in functional genomic analysis and drug development for proteinopathies.

## In brief

Intracellular proteinaceous inclusions in neurodegeneration are heterogeneous. Lam, Ndayisaba, et al. developed an iPSC toolbox to rapidly induce CNS cells with inclusions. α-Synuclein “inclusionopathy” models, with gene- and protein-interaction screens, uncover novel inclusion subtypes, their molecular markers, and their effects on neuronal survival. Biological relevance was established in Parkinson’s postmortem brains.

## Graphical abstract

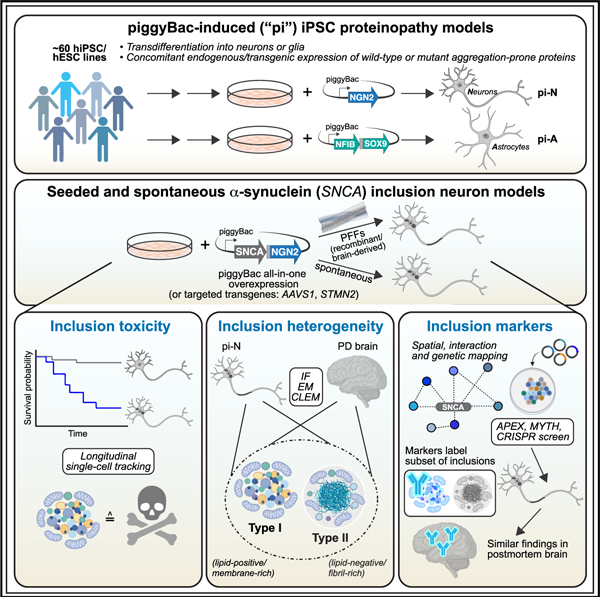

## INTRODUCTION

Neurodegenerative diseases—such as Alzheimer’s disease (AD), Parkinson’s disease (PD), or frontotemporal dementias—are named “proteinopathies” because their hallmark pathology is widespread protein-rich inclusions within various neuronal and glial subtypes of the central nervous system (CNS).^1^ Much attention has been focused on the β-pleated sheet-rich fibrils within these inclusions because they can self-template and may contribute to disease progression. Disease-specific higher-order folds of these amyloids (“strains”) can lead to distinct distributions of cellular pathologies in rodent models and, potentially, different disease phenotypes in humans.^2–5^ Beyond this conformational diversity, histopathologic analysis of postmortem brains reveals a multitude of inclusion morphologies. For example, in “synucleinopathies,” characteristic α-synuclein (αS)-rich inclusion pathologies, including Lewy bodies (LBs), are found in brainstem, cortical, and autonomic neurons in PD and Lewy body dementia. These pathologies can be found in neuronal somata or in neurites. By contrast, entirely distinct types of neuronal and oligodendroglial cytoplasmic and nuclear inclusions are found in another synucleinopathy, multiple system atrophy (MSA).^6^ Inclusions are similarly diverse in other proteinopathies.^7^

Proteinaceous inclusions can even comprise distinct ultra-structures within the same cell type. For example, membrane-rich αS inclusions (“pale bodies” [PBs]) and fibril-rich LBs can coexist in the same neuron, even at the earliest prodromal stages of the disease,^8–10^ and membrane-predominant inclusion pathologies are frequent in PD brains.^11^ While PBs are less uniformly ubiquitinated than LBs,^10^ both αS inclusions stain avidly for standard markers like αS phosphorylated at serine 129 (pS129). Thus, very different inclusion subtypes are indistinguishable by standard neuropathologic markers. These subtypes may have biologically distinct effects, potentially explaining why inclusions and neurodegeneration are not always correlated.^12–15^ Inclusions have been described as protective^16,17^ or detrimental.^18,19^ Both may be true, and a finer-grained classification of inclusion subtypes could help explain the dichotomy.

Induced pluripotent stem cell (iPSC)-derived CNS models present a potential patient-specific model system for visualizing inclusion formation in real time, counteracting limitations of end-stage postmortem analysis. However, current human iPSC models have limited tractability, often requiring lengthy differentiation. Mature inclusions do not form in a reasonable time frame.^20,21^ When iPSC-derived neurons have been exposed to amyloid “seeds,” the heterogeneity of inclusions and relationship to brain pathology has also not been thoroughly addressed.^22–24^ Here, we present “inclusionopathy” models that combine inclusion formation with one-step transdifferentiation from iPSCs to CNS cells. We use scalable and virus-free expression of transdifferentiation factors with Gateway-compatible piggyBac (pB) vectors. Concomitant expression of aggregation-prone proteins at levels similar to human brain is achieved through all-in-one pB transgenes or transgenes targeted to specific genomic loci. We present ~60 pB-transfected lines for directed iPSC-to-CNS transdifferentiation into cortical neurons or astrocytes.

Exemplar cortical αS inclusionopathy models demonstrate rapid (~2 weeks) induction of either spontaneously forming lipid-rich inclusions or exogenously seeded fibril-rich inclusions. These models are amenable to longitudinal tracking at single-cell and single-inclusion resolution. They reveal subclasses of inclusions in matched familial and sporadic PD brains. One class of p62-/ubiquitin-positive fibril-rich inclusions is relatively stable in neurons and neuroprotective. By contrast, a lipid-rich class is highly dynamic and toxic. Finally, genome-scale CRISPR screens and systematic αS protein-interaction mapping pinpoint key proteins, including RhoA, that are likely toxic when sequestered into specific inclusion subtypes. These models shed light on the dynamic interactions between αS inclusions, molecular interactions within them, and biologically important subtypes in postmortem brain. We anticipate that scalable inclusionopathy models will be useful for biological and drug discovery in neurodegeneration as well as functional genomics and personalized medicine.

## RESULTS

### A robust pB-based expression vector facilitates iPSC transdifferentiation

**Figure 1.**
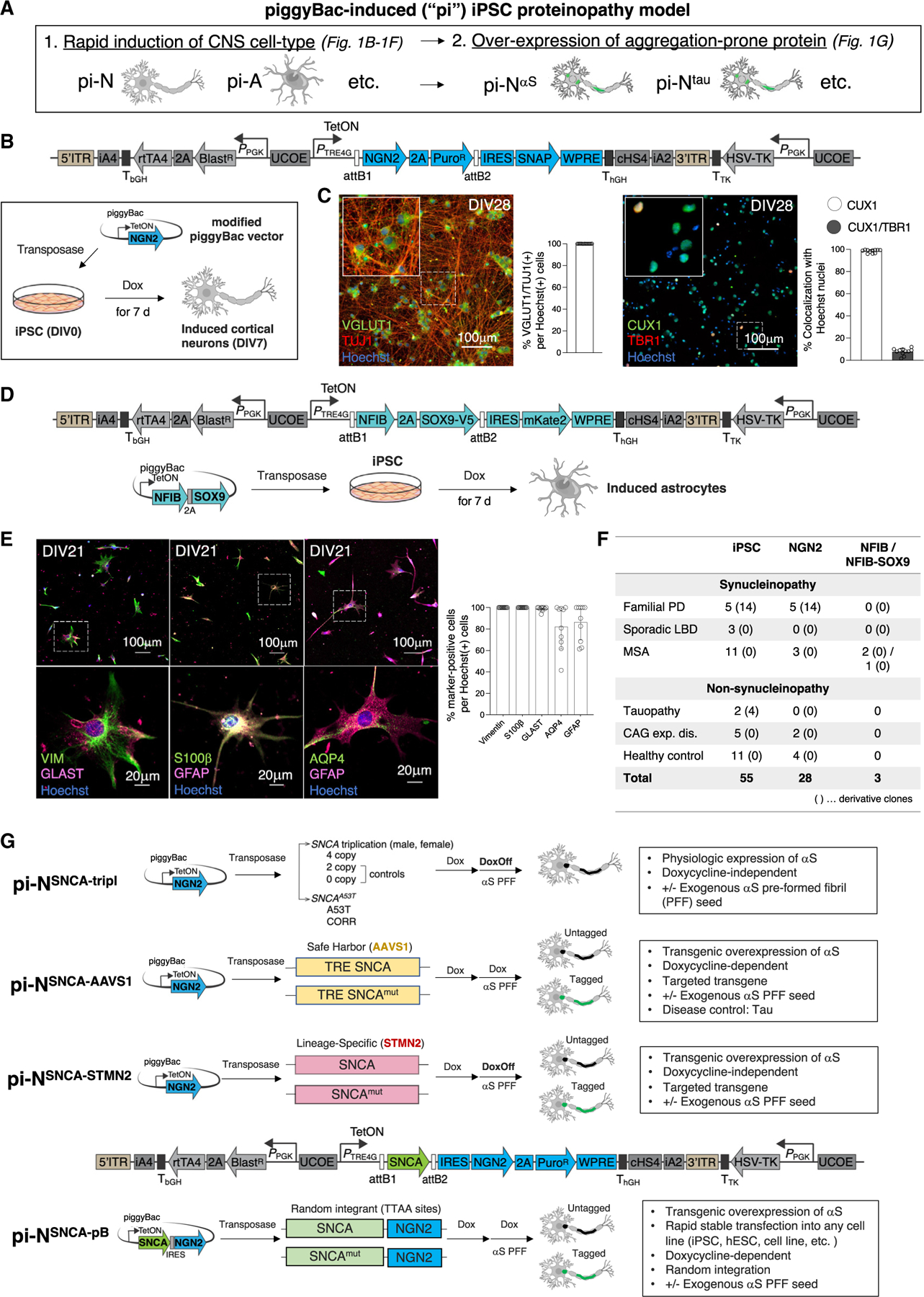
Overview of piggyBac-induced iPSC proteinopathy models (A) Classification of piggyBac-induced (pi) iPSC proteinopathy model system. (B) A modified piggyBac (pB) vector for transdifferentiation of iPSCs into cortical neurons. (C) Immunofluorescence (IF) staining and quantification of neurons (pi-Ns) transdifferentiated from H9 hESC confirm cortical glutamatergic neuron identity (layer II/III). (D) Modified pB vector with NFIB-SOX9 allows iPSC transdifferentiation into astrocytes. (E) Left, IF of H9 hESC-derived pB-induced astrocytes (pi-A) stained for canonical astrocyte markers. Right, IF quantification. (F) Summary of iPSC lines introduced with pB-NGN2, pB-NFIB, or pB-NFIB-SOX9. CAG exp. dis., CAG expansion disease. (G) Overview of proteinopathy platform, including pathologic (but endogenous) versus transgenic overexpression through targeting to a safe harbor locus (*AAVS1*), a lineage-specific locus (*STMN2*), or pB random integration. Quantification of (C) and (E): 4 (C) and 3 (E) independent replicates each across 3 separate neuronal differentiations. Error bars = SD.

Our “inclusionopathy models” combined (1) directed transdifferentiation in which forced expression of lineage-specific transcription factors in iPSCs/human embryonic stem cells (hESCs) rapidly and reproducibly generates distinct CNS cell types (Figure 1A, left)^25,26^ with (2) rapid induction of inclusions through transgenic expression of the aggregation-prone toxic protein of interest (Figure 1A, right).

For transdifferentiation, we settled upon a pB delivery system because it was virus-free and scalable, had large cargo capacity, and could be integrated with simple transfection. We coupled it with Gateway cloning, enabling straightforward shuttling in of different transgenes. We introduced key modifications to the pB integrating vector to ensure stable and high expression levels of the transgene cargo.^27^

For proof of principle, we selected CNS cell types for which robust methods for lentiviral vectors have been published—NGN2 for layer II/III cortical glutamatergic neurons (Figures 1B and 1C)^26^ and NFIB and SOX9 for astrocytes (Figure 1D).^25^ hESC-derived neurons with pB Ngn2 (pB-NGN2) exhibited appropriate markers for glutamatergic cortical neuron fate (Vglut1 and Tuj1/β-tubulin III) and for superficial (Cux1; layer II/III) but not deep (Tbr1; layer V) cortical layers (Figure 1C). pB-NFIB-SOX9 or pB-NFIB expression in hESCs resulted in astrocytic cell type-specific markers—glial fibrillary acidic protein (GFAP), S100β, vimentin, glutamate aspartate transporter 1, and aquaporin 4 (AQP4)—compared with parental hESC lines (Figures 1E and S1A–S1C). We refer to these as pB-induced neurons (pi-Ns) and pB-induced astrocytes (pi-As). We assembled approximately 100 pluripotent stem cell lines, from hESCs to hiPSCs (Mass General Brigham Stem Cells in Neurodegeneration [SCiN] study), across multiple neurodegenerative diseases (Figures 1F and S1L; Table S1). We introduced pB-NGN2 into 56 iPSC lines and derivative clones, pB-NFIB into 2 iPSC lines, and pB-NFIB-SOX9 into 1 iPSC line (Figure 1F; Table S1).

### iPSC inclusionopathy models are generated through concomitant transdifferentiation and ectopic expression of aggregation-prone proteins

We utilized multiple complementary methods to express aggregation-prone proteins, each with distinct advantages. αS served as the exemplar case, though we have also generated tau, β-amyloid, and ApoE constructs (Figures 1G and S1L; Table S1; data not shown). In a more physiologic overexpression system, we integrated pB-NGN2 into iPSCs reprogrammed from female^27^ and male^28,29^ Iowa kindred patients harboring *SNCA* gene triplication with concomitant dementia and parkinsonism associated with diverse pathologies.^30,31^ Isogenic allelic series were generated through CRISPR-Cas9 engineering (*SNCA* “4-copy” parental, wild-type “2-copy” knockdown, and null “0-copy” knockout) (Figures 1G, top, and 2A, left). We also introduced pB-NGN2 into iPSCs generated from a patient harboring the *SNCA* A53>T mutation (A53T) or its isogenic mutation-corrected control line (CORR)^27^ (Figures 1G, top, and 2A, right). In these models, doxycycline is only required for 7-day Ngn2 expression.

We coupled Ngn2 with transgenic expression of aggregation-prone proteins. For proof of concept, we targeted αS or tau transgenic constructs under a tetracycline response element (TRE) to the *AAVS1*/*PPP1R12C* locus on one allele^32^ (Figures 1G, second from top, and S1D). The rtTA transactivator for tetracycline-inducible expression was targeted to the same locus of the other allele. Targeting was verified by Southern blot (Figure S1E) and expression was doxycycline dose dependent (Figure S1D, inset). The advantage here is that a single copy of the αS or tau transgene is targeted to a defined locus. Thus, different proteins or mutations can be cross-compared at equivalent expression levels. However, the system was limited by silencing of the TRE-driven transgene (Figure S1F) and poor expression in certain cell types, including astrocytes (Figure S1G). Introduction of pB-NGN2 (Figures S1F, right, and S1H–S1J; Table S2) overcame this problem, with αS transgene expression in >95% of pi-Ns at DIV25 (pi-N^SNCA-mK2-AAVS1^).

For lineage-specific and doxycycline-independent expression, we CRISPR-targeted *SNCA-GFP* to the 5′UTR of the *STMN2* locus (Figure 3G), a gene with highly selective expression in neurons (Brain RNA-Seq, brainrnaseq.org)^33^ (Figures 1G, third from top, and S1K, left). This does not lead to reduction in *STMN2* expression (Figure S1K, right). Doxycycline is only required to induce pB-NGN2 expression for 7 days. Combining this system with pB-NGN2 enabled transgene expression in >95% of pi-Ns at DIV25 (Figure S1H).

Finally, to avoid the need to target each line by genome editing, we opted for a simple all-in-one pB plasmid. This contained both the transgene expressing the aggregation-prone protein as well as the transcription factor driving differentiation, separated by an IRES sequence (Figure 1G, lowermost).

### Induction of αS inclusions through amyloid seeds is enhanced by pB-based transgenic expression of αS

**Figure 2.**
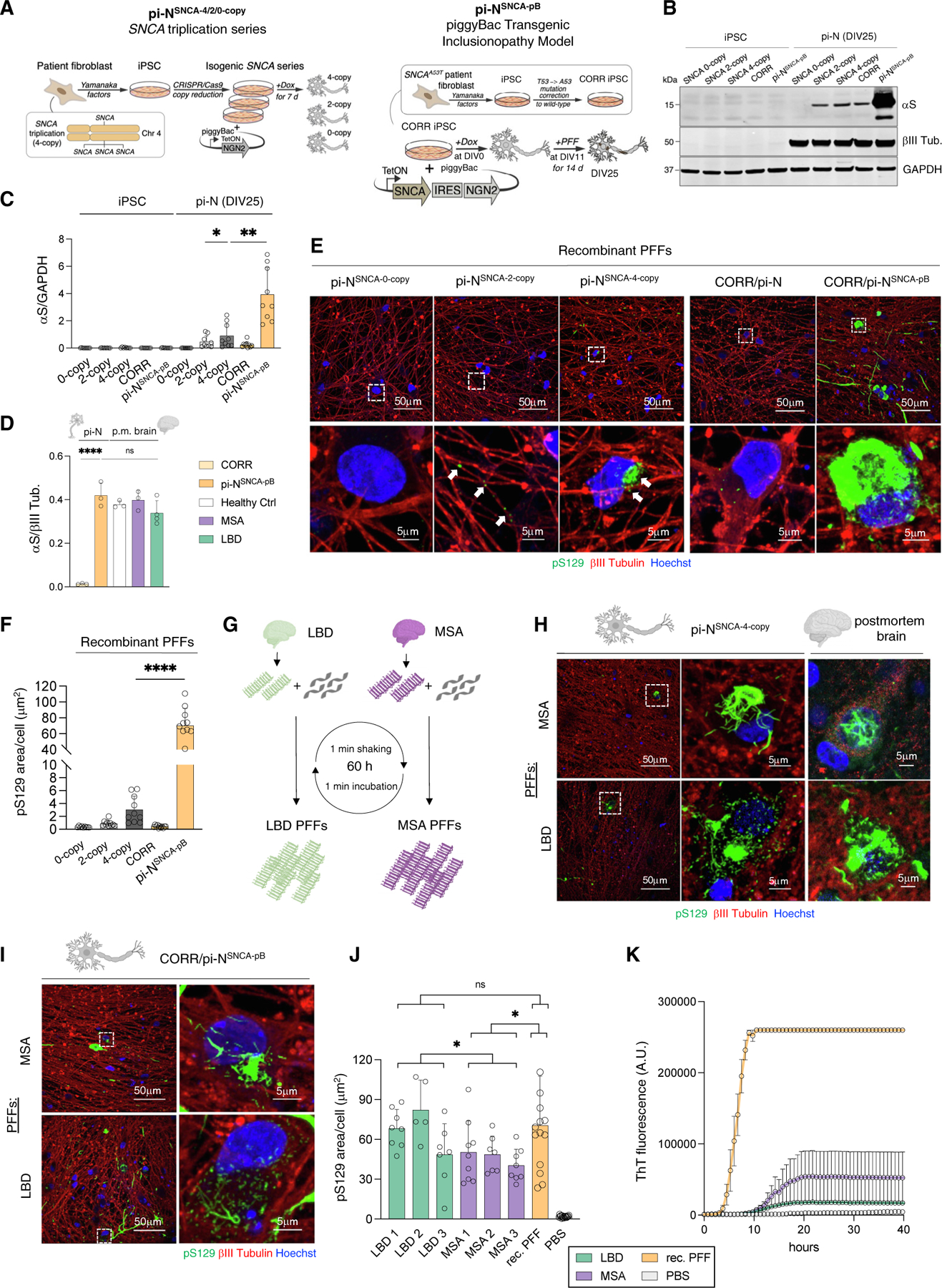
αS inclusion formation through amyloid seeds is enhanced by pB-based αS overexpression (A) Schematic diagrams of pathologic overexpression (*SNCA* 4-copy) (left) and pB transgenic (right) proteinopathy models. Left, generation of isogenic lines with different *SNCA* copy numbers (pi-N^SNCA−4/2/0-copy^) by CRISPR-Cas9 gene knockout. pB-NGN2 integration allows neuronal transdifferentiation. Right, generation of a mutation-corrected line (CORR) from an A53T familial PD patient (inset). All-in-one pB-SNCA-IRES-NGN2 integration into CORR iPSC line facilitates doxycycline-inducible overexpression of αS (pi-N^SNCA-pB^). (B) αS western blot in pi-N models and iPSCs. (C) Quantification of αS levels from (B); paired t test: *p < 0.05, **p < 0.01. (D) Quantification of western blot in pB neurons versus postmortem brain lysate (frontal cortex) from 3 control, 4 LBD, and 3 MSA brains. Related to Figure S2C. (E) αS-pS129 IF in PFF-seeded cortical neurons. Arrows in pi-N^SNCA−4/2-copy^ indicate pS129(+) inclusions. (F) Quantification of (E). (G) Schematic of seed amplification assay (SAA) from LBD and MSA postmortem brain. (H) αS-pS129 IF in pi-N^SNCA−4-copy^ model (left and center) seeded with MSA or LBD αS-PFFs versus postmortem PD and MSA brain inclusions (right). (I) αS-pS129 IF in transgenic pi-N^SNCA-pB^ model seeded with MSA or LBD αS-PFFs. (J) Quantification of (I) (MSA PFFs [*n* = 3], LBD PFFs [*n* = 3]). (K) SAA reamplification of CORR/pi-N^SNCA-pB^ neuronal lysates previously seeded with recombinant, MSA, and LBD PFFs (*n* = 3 each) for 14 days. One-way ANOVA plus Tukey’s multiple comparison test for (D), (F), and (J): *p < 0.05, ****p < 0.0001. Experimental replicates: 3 (C), 3–4 (F), and 2–4 (J) independent replicates each across 3 separate neuronal differentiations. Error bars = SD.

We exposed pi-Ns to αS preformed fibrils (PFFs), a standard way to accelerate αS amyloid pathology.^34–37^ We conducted a head-to-head comparison of non-transgenic expression (*SNCA* triplication series) versus pB transgenic models. For the latter, we genetically corrected an A53T patient iPSC line to generate a “synucleinopathy-permissive” genetic background^27^ (Figure 2A, right) and introduced our all-in-one pB expressing wild-type αS, henceforth “pi-N^SNCA-pB^” (Figure 1G, lowermost). This line was compared with the triplication allelic series transfected with pB-NGN2 alone (Figures 2A, left, and 1G, uppermost; pi-N^SNCA−4/2/0-copy^). Both models exhibited the appropriate markers for superficial cortical glutamatergic neuron fate (Figures S2A and S2B). The pi-N^SNCA-pB^ model exhibited substantially higher αS steady-state protein level than the pi-N^SNCA−4-copy^ model (Figures 2B and 2C). Importantly, αS levels normalized to neuronal β-tubulin III in this pB model were more closely matched to postmortem brain (frontal cortex) of synucleinopathy cases and controls (Figures 2D and S2C). This underscores the relatively low levels of endogenous αS expression in iPSC-derived neurons under standard differentiation conditions.

We triggered inclusion formation by exposing DIV11 pi-Ns (with Ngn2 induction day defined as DIV0) to recombinant αS PFFs (Figures S2D and S2E). Inclusion deposition indicated by pS129 immunostaining was far more robust in the pi-N^SNCA-pB^ model compared with the pi-N^SNCA−4-copy^ model, within both neurites and soma (Figures 2E and 2F). By contrast, there was no seeding in lines without αS (pi-N^SNCA−0-copy^), minimal at wild-type levels of αS (pi-N^SNCA−2-copy^, arrows; pi-N^CORR^), and none in simply PBS-treated lines (except for spontaneous inclusion formation in pi-N^SNCA-pB^; addressed below) (Figure S2F).

Next, we amplified αS fibrils from synucleinopathy brains (3 MSA and 3 Lewy body disease [LBD] cases)^38^ through a seed amplification assay (SAA)^39,40^ (Figure 2G). The resultant αS fibrils (Figure S2G) were comparable to the insoluble fraction of the matching brain lysate on western blot following proteinase K digestion (Figure S2H). Seeding pi-N^SNCA−4-copy^ and pi-N^SNCA-pB^ models with MSA- and LBD-brain-derived PFFs resulted in inclusions reminiscent of postmortem brain^41^ (MSA, “skein-like” perinuclear neuronal inclusion; LBD, diffuse intraneuronal inclusion) (Figures 2H and 2I). MSA PFFs from these 3 cases demonstrated slightly lower seeding efficiency in our pi-N^SNCA-pB^ model when compared to LBD and recombinant PFFs (Figure 2J). Repeat SAA with thioflavin T (ThT) on neuronal lysates confirmed that αS could be reamplified after “passaging” from pi-N^SNCA-pB^ lines previously seeded with recombinant or brain PFFs (Figures 2K and S2H).

Thus, in the pB transgenic expression system, αS levels more closely matched to brain result in rapid self-templating inclusionopathy reminiscent of human postmortem brain.

### Formation of seeded pS129-positive αS-A53T inclusions is dependent on αS NAC domain

**Figure 3.**
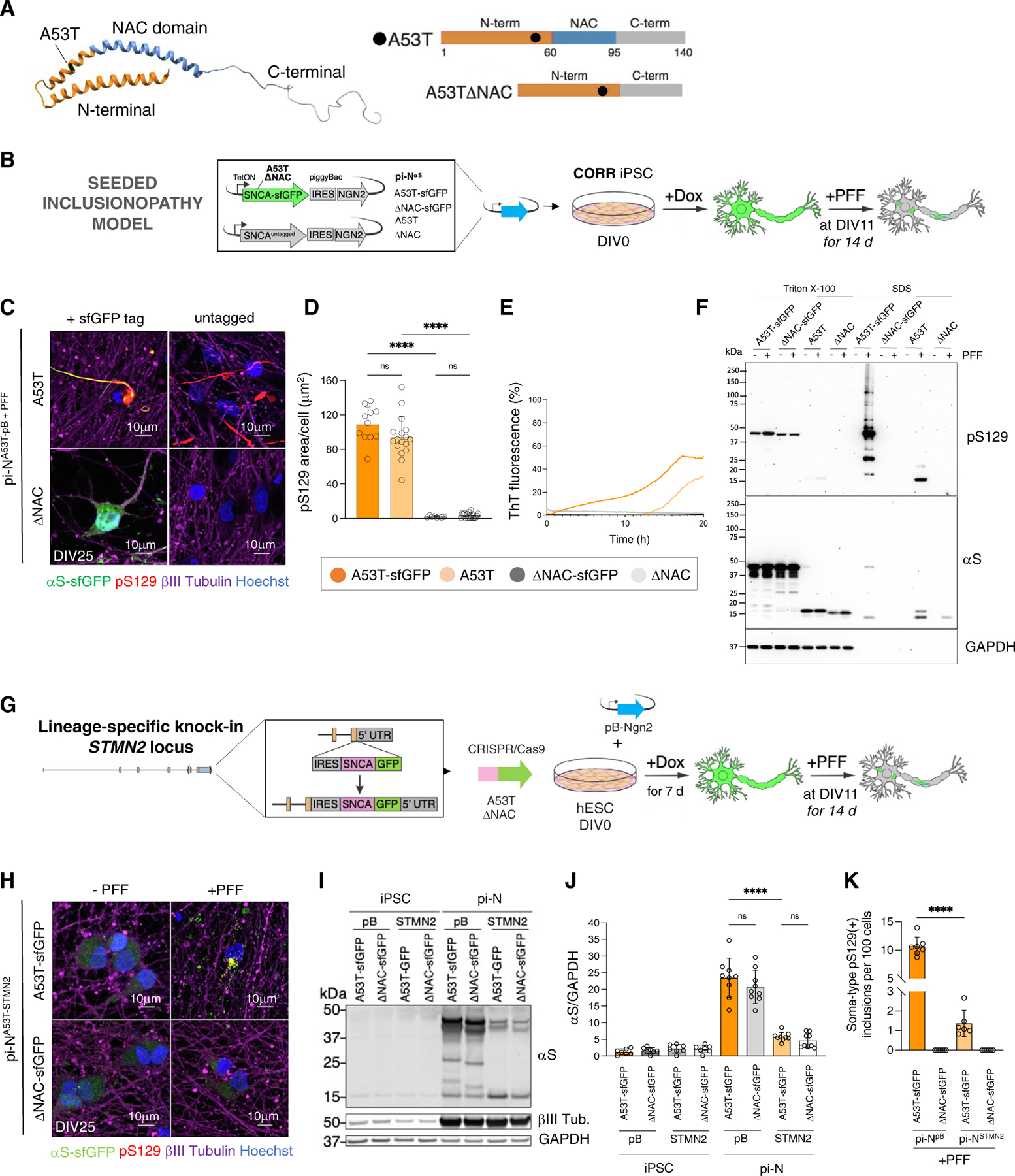
Characterization of PFF-seeded inclusionopathy model (A) αS protein structure (PDB: 1xq8) juxtaposed with linear maps of αS-A53T and αS-A53T-ΔNAC indicating relevant amino acid positions. (B) Schematic outline of seeded inclusionopathy model. (C) pS129 IF in PFF-seeded inclusionopathy model overexpressing sfGFP-tagged or untagged αS. (D) Quantification of (C). (E) SAA reamplification of insoluble αS in PFF-seeded transgenic neurons. (F) Western blot for total αS and pS129 after sequential Triton X-100/SDS extraction of soluble and insoluble protein fractions. (G) Schematic outline of *STMN2*-driven transgenic αS overexpression. (H) pS129 IF in PFF-seeded versus unseeded *STMN2* transgenic models. (I) αS western blot in pB versus *STMN2* transgenic lines. (J) Quantification of (I). (K) Quantification of pS129(+) soma-type inclusions in PFF-seeded pB or *STMN2* transgenic neurons. One-way ANOVA plus Tukey’s multiple comparison test for (D), (J), and (K): *p < 0.05, **p < 0.01, ***p < 0.001, ****p < 0.0001; ns, not significant. Experimental replicates: 3–5 (D), 3 (J), and 3 (K) independent replicates each across 3 separate neuronal differentiations. Error bars = SD.

Mutations in αS can alter its tendency to aggregate and bind membranes.^42,43^ We explored such mutations with our pi-N system. We also compared untagged αS versus αS tagged with superfolder GFP (sfGFP).^44^ sfGFP is large (26.8 kDa) relative to αS (14 kDa) and could alter αS aggregation properties, although prior studies suggested otherwise^45^ and known physiologic αS-protein interactions are faithfully recovered when αS is tagged with similarly large APEX2 (28 kDa).^46^ The A53T familial αS point mutation increases the propensity of αS to aggregate and fibrillize.^43^ Moreover, A53T with the “non-amyloid component domain” deleted (ΔNAC)^47^ is unable to do so (Figure 3A). We expressed these with our all-in-one pB construct (Figure 1G, lowermost) in the CORR iPSC line. Transgenes were either sfGFP tagged or untagged (Figure 3B). Steady-state protein levels were similar between tagged and untagged A53T transgenes, although they were reproducibly higher in ΔNAC-sfGFP compared with ΔNAC (Figure S3A).

Initially, we did not appreciate spontaneous inclusion formation in these neurons (Figure S3B). We thus induced inclusion formation with exogenous recombinant αS PFFs. Seeding with A53T αS PFFs for 14 days (DIV11–DIV25) triggered inclusion formation in A53T pi-Ns but not in ΔNAC (Figures 3C and 3D). pS129 signal colocalized with sfGFP signal in the A53T-sfGFP line (Figure 3C). Seeded amplification occurred within 12 h for both sfGFP-tagged and untagged pi-N^A53T-pB+PFF^ models but not in ΔNAC controls (Figure 3E). Sequential extraction into Triton X-100 (TX-100) and SDS fractions, and subsequent immunoblotting for pS129 and total αS,^34^ revealed TX-100-insoluble (SDS-soluble) αS in pi-N^A53T-pB+PFF^ but not in pi-N^ΔNAC-pB+PFF^ models, regardless of tagging. Without PFFs, αS was only detected in the TX-100-soluble fraction (Figure 3F). C-terminal tagging of αS-A53T with sfGFP modestly increased the formation of higher molecular weight species compared with untagged αS (Figures 3C–3F). PFF-induced inclusion formation in pi-N^A53T-pB+PFF^ cultures was not associated with cellular pathologies previously tied to synucleinopathy, including mitochondrial respiration, mitochondrial subunit expression, and lysosomal flux,^48–51^ despite some differences in unseeded ΔNAC versus A53T conditions (Figures S3C–S3G).

We compared two transgenic inclusionopathy models: the all-in-one pB versus single-copy *STMN2* integrants (Figures 1G, third from top, and 3G). *SNCA* transgene knockin at the *STMN2* locus exhibited neuron-specific expression indicated by colocalization with MAP2 (Figure S4A) but not with astroglial markers (in forebrain organoids data not shown). Transdifferentiation of pi-N^A53T-sfGFP-STMN2^ into cortical neurons with pB-NGN2 gave rise to the appropriate markers for glutamatergic cortical neuron fate and superficial cortical layers (Figures S4B and S4C). As with the pi-N^SNCA-pB^ line, PFF seeding of pi-N^STMN2^ A53T developed pS129(+) inclusions, whereas neurons expressing ΔNAC did not (Figure 3H). Higher expression levels correlated with considerably more abundant pS129(+) inclusions in the pi-N^SNCA-pB^ line compared with the pi-N^SNCA-STMN2^ line (Figures 3I–3K and S4D). While the *STMN2* neurons and triplication pi-Ns offer lineage specificity and doxycycline independence, we selected the pi-N^A53T-sfGFP-pB^ line for further investigation in this study because of higher efficiency of inclusion formation.

### Inclusion subtypes within pB-induced inclusionopathy models recapitulate those within postmortem synucleinopathy brain

Beyond pS129, αS-rich inclusions comprise many other molecular components,^6,41,52^ including ubiquitin and the ubiquitin-binding p62 protein that transports targets for degradation by autophagosomes.^53,54^ While p62 and ubiquitin labeling are often considered to go together, prior comparisons between ubiquitin and p62 in nigral inclusions hint at differences in immunoreactivity among inclusions.^55^

**Figure 4.**
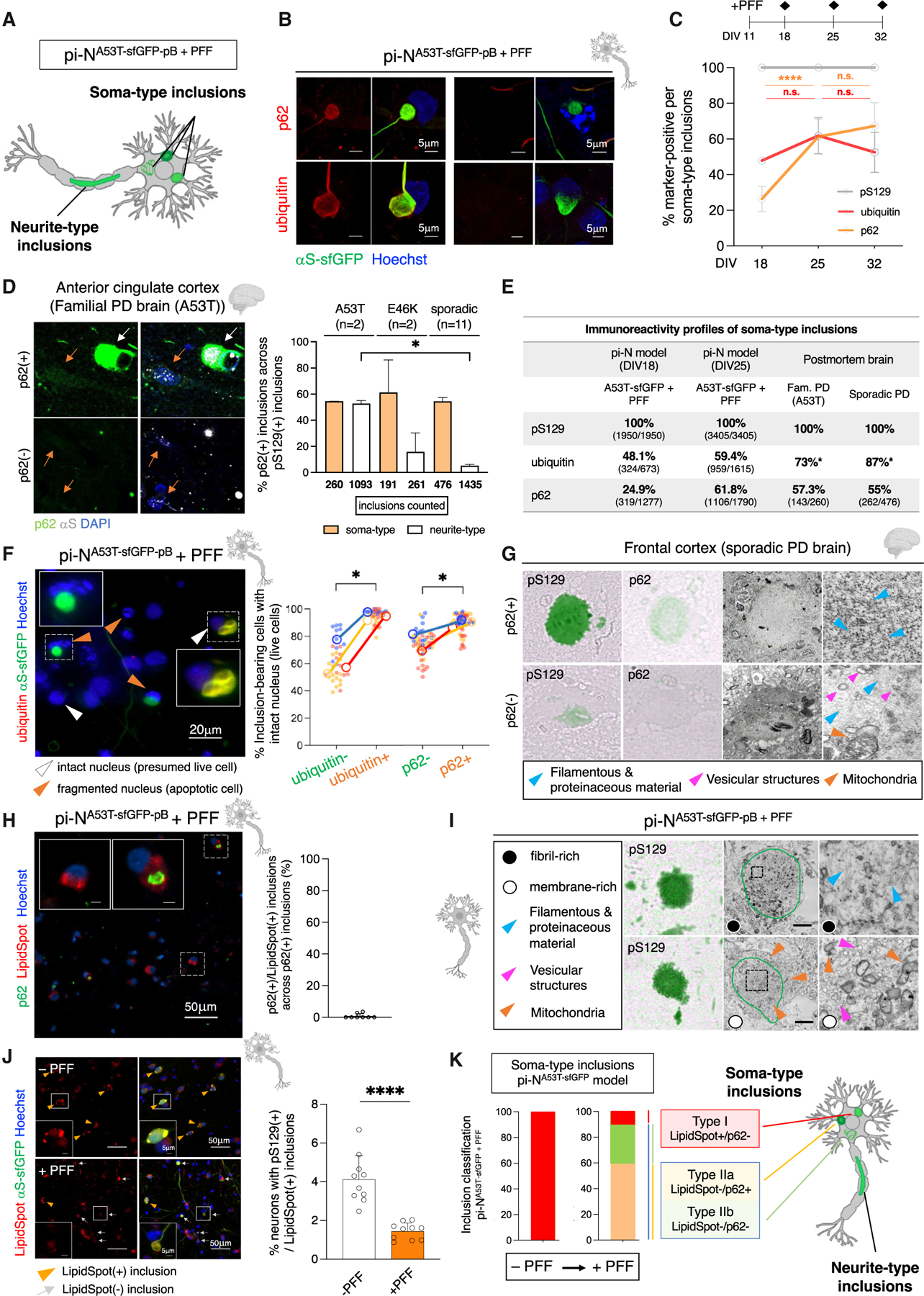
Inclusion classification is conserved from pi-N model to postmortem brain (A) Inclusion classification by subcellular location. (B) Ubiquitin and p62 IF of soma-type inclusions in seeded inclusionopathy model. (C) Quantification of pS129, p62, and ubiquitin inclusion immunopositivity in seeded pi-Ns. One-way ANOVA plus Tukey’s multiple comparison test: ***p < 0.001, ****p < 0.0001; n.s., not significant. (D) Left, p62 and αS IF in familial PD brain. Orange arrows, cells with p62(−) inclusions; white arrow, p62(+) inclusion. Right, frequency of p62(+) inclusions in soma and neurites in cingulate cortex of familial PD and sporadic postmortem brains. (E) Immunoreactivity profiles of soma-type inclusions in pi-N model and postmortem PD brain. *Ratios of ubiquitin(+)/pS129(+) inclusions were inferred from pS129/p62 and p62/ubiquitin double stains. Numbers in parentheses indicate total number of inclusions counted. (F) Cross-sectional analysis of seeded inclusionopathy model (DIV25) to evaluate association of inclusion p62 and ubiquitin immunopositivity with intact nuclei (presumed live cell) versus fragmented nuclei (presumed dead cell). Paired t test: *p < 0.05. (G) CLEM for pS129 and p62 in frontal cortex of sporadic PD brain. (H) IF for p62 and LipidSpot in pi-N model. (I) CLEM for pS129 in seeded inclusionopathy model. (J) Left, IF for LipidSpot in pi-N^A53T-sfGFP-pB^ model. Right, quantification of IF. One-way ANOVA plus Tukey’s multiple comparison test: ****p < 0.0001. (K) Inclusion subtype classification in seeded inclusionopathy model. Type II inclusions data are from (F). Experimental replicates: 3 (B), 3 (F), 2–3 (H), 3–4 (J), and 2–3 (K, quantification of Type I LipidSpot(+) inclusions) independent replicates each across 3 separate neuronal differentiations. Error bars = SD.

In our PFF-seeded tagged (pi-N^A53T-sfGFP-pB^) or untagged (pi-N^A53T-pB^) inclusion models, most inclusions appeared as thread-like along neurites (Figures 3C and 4A). Distinct subtypes of somatic inclusions also formed (Figure 4A), distinguishable by ribbon-like or punctate morphologies and empirically different resistance to detergents during immunostaining (e.g., 0.2% TX-100 or 0.1% saponin, data not shown). We thus quantitated inclusion markers pS129, p62, and ubiquitin longitudinally. While all inclusions stained positive for pS129 (Figures 4E and S4E), they surprisingly differed according to staining with p62 or ubiquitin markers (Figures 4B and S4F–S4I). One week after seeding with PFFs, ~48% of inclusions in pi-N^A53T-sfGFP-pB^ soma were ubiquitin(+), whereas only a minority (~25%) were p62(+) (Figure 4C, DIV18). At DIV32, the frequency of p62(+) inclusions increased to ~70%. Similar trends were detected in the untagged pi-N^A53T-pB^ model (Figure S4J). Thus, in our models, ubiquitination likely precedes p62 labeling of seeded inclusions.

We next asked whether this inclusion heterogeneity is also a feature in postmortem A53 > T brain. Such specimens are rare and often suboptimally preserved (e.g., delipidated and poorly amenable for EM). We thus cross-compared brains of 2 patients with A53T αS mutation, 2 patients with E46K αS mutation (the only 2 now known to be available for analysis worldwide), and 11 patients with sporadic PD.^56^ We analyzed >3,700 distinct cortical somatic and neuritic pS129(+) inclusions across frontal cortices of these brains. We confirmed that, just as in our inclusionopathy models, p62 is far from being homogeneous (Figures 4D and 4E). While the limited number of postmortem brain samples precluded statistical tests, the frequency of somatic and neuritic p62(+) inclusions appeared to be similar in A53T postmortem samples and trended toward far fewer p62(+) neurite-type inclusions in E46K or sporadic PD (Figure 4D).

We investigated the relationship of ubiquitin(+) or p62(+) inclusions to survival, an analysis that cannot be performed in brain. We quantified cells with intact nuclei (presumed live cells, detected with Hoechst) and fragmented nuclei (presumed apoptotic cells) (Figures 4F, left, and S4K). Ubiquitin(+) and p62(+) inclusions were more likely associated with intact neuronal nuclei than ubiquitin(−) and p62(−) inclusions. Since the proportion of p62(+) inclusions in our cultures increased over time (Figure 4C), our data suggest that ubiquitin(+) and p62(+) inclusions may be neuroprotective (Figures 4F, right, and S4L).

To characterize ultrastructure, we employed correlative light and electron microscopy (CLEM) in frontal cortex of sporadic PD cases. This method enables ultrastructural and immunohistochemical analysis of the same inclusion. pS129(+)/p62(+)-immunopositive inclusions were enriched in filamentous and proteinaceous material, whereas pS129(+)/p62(−) inclusions contained a mixture of vesicular structures, mitochondria, and filamentous material (Figure 4G). Double staining and quantification of pi-N A53T models for p62 and the neutral lipid dye LipidSpot confirmed negligible colocalization (6/236 p62(+) inclusions) (Figure 4H).

These data are consistent with recent CLEM data in brain showing that some αS(+) inclusions comprise a dense fibril-rich core, while others are rich in clustered vesicles and dysmorphic organelles.^11^ In our models, both sfGFP-tagged and untagged pi-N^A53T-pB^ models included a fibrillar class of somatic inclusions (Figures 4I, top, and S5B, top) versus another class composed of clustered vesicles, often interspersed with lipid droplets and containing dysmorphic mitochondria (Figures 4I, bottom, and S5B, bottom). By contrast, neurite-type inclusions comprised dysmorphic mitochondria and likely disrupted neurofilaments as revealed by GFP-immunogold labeling in the seeded pi-N A53T inclusion model (Figure S5A).^11^

Thus, inclusions in the seeded pi-N A53T model are heterogeneous and comprise fibril- and membrane-rich subtypes, reminiscent of inclusions in sporadic and A53T postmortem brain (Figures 4G and S5C).

### Spontaneous membrane and lipid-rich (Type I) inclusions are dynamic in contrast to seeded Type II inclusions

Upon closer scrutiny, we noted that LipidSpot(+) (i.e., lipid-rich) inclusions occurred in this model prior to seeding with PFFs, regardless of tag. Thus, lipid-rich inclusions form spontaneously upon αS-A53T overexpression (Figures 4J, S3B, and S5D). Surprisingly, treatment with PFFs led to reduction in LipidSpot(+) soma-type inclusions despite an increase in the overall frequency of pS129(+) inclusions (Figures 4J and S5D, right). Addition of exogenous PFFs led to a change from purely p62(−) lipid-rich inclusions to a mixture of p62(+) and p62(−) inclusions (Figure 4K).

We developed a subclassification of inclusions: Type I LipidSpot(+)/p62(−), Type IIa LipidSpot(−)/p62(+), and Type IIb LipidSpot(−)/p62(−). At DIV25 in the pi-N^A53T-sfGFP-pB^ model, without seeding, all pS129(+) inclusions were Type I (Figure 4K, left). After seeding, the frequencies of Types I and IIa pS129(+) inclusions were, respectively, 12.86% ± 7.7% (SD) and 61.26% ± 9.0% (SD) across 3 biologically distinct differentiations, with the remaining 26% being Type IIb inclusions (Figures 4J and 4K). LipidSpot staining in pi-N^ΔNAC-sfGFP-pB^ and pi-N^sfGFP-pB^ controls revealed either diffuse intracellular labeling or sparse smooth circular structures consistent with physiologic lipid droplets (Figure S5E).

**Figure 5.**
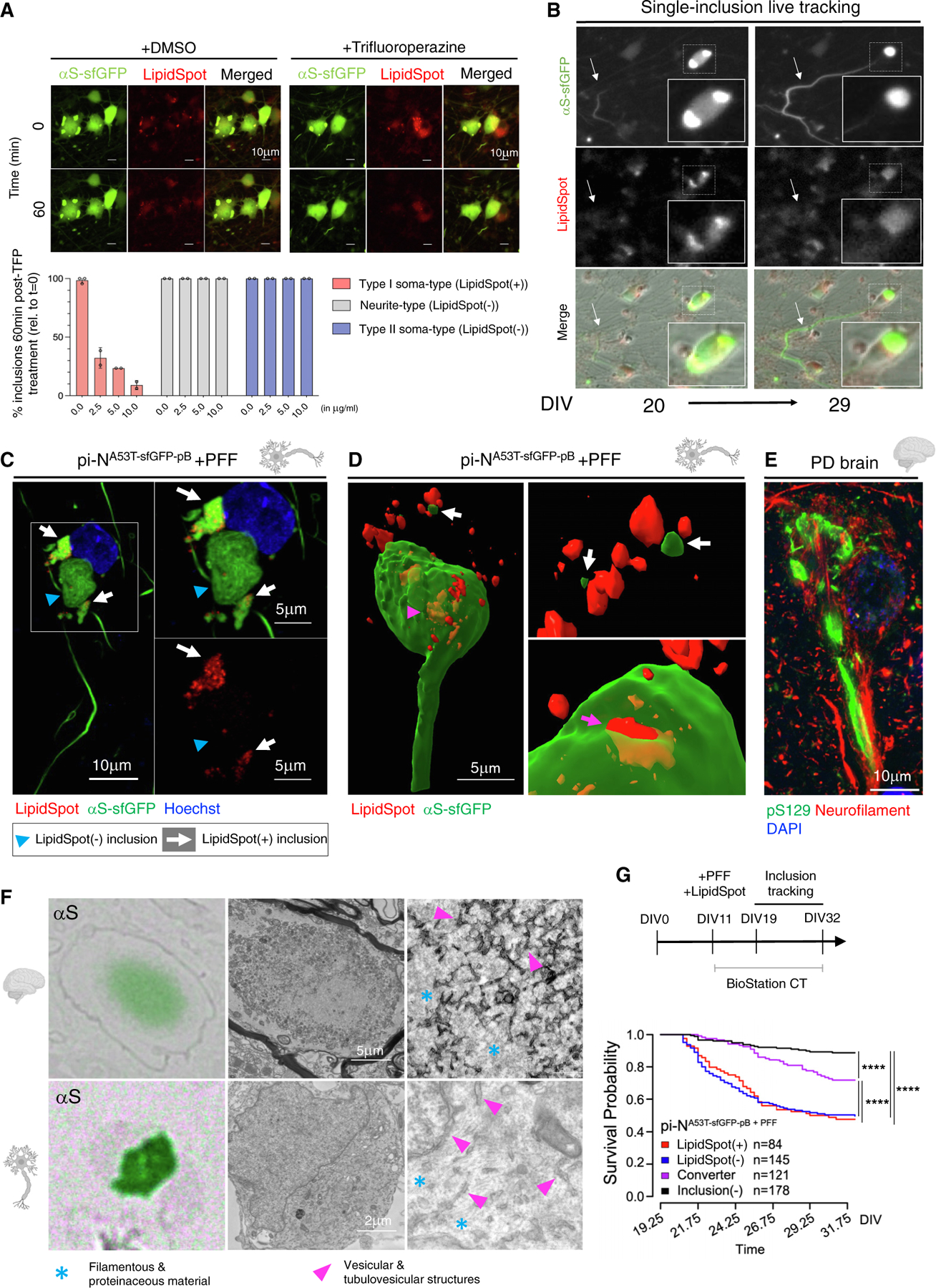
Fusion events between lipid-rich and presumed fibril-rich inclusions (A) Top, time-lapse imaging of LipidSpot(+) inclusions pre- (T = 0 min) and post-treatment (T = 60 min) with DMSO or trifluoperazine. Bottom, quantification of LipidSpot(+), LipidSpot(−) neurite-type, or soma-type inclusions at T = 60 min post-treatment (3 independent replicates per condition, reproduced across 3 neuronal differentiations using the highest treatment concentrations). (B) Time-lapse imaging in seeded PFF model capturing interaction between LipidSpot(+) inclusions and elongating neurite-type inclusion in the same cell. White arrow, neurite-type inclusion (GFP(+)/LipidSpot(−)). Inset, cell soma with two GFP(+)/LipidSpot(+) inclusions (DIV20), which become one GFP(+)/LipidSpot(−) inclusion (DIV29). (C) Confocal image of adjacent LipidSpot(+) and LipidSpot(−) inclusions. (D) Dynamic lattice light-sheet microscopy (3D rendering) of a LipidSpot(+)/GFP(+) soma-type inclusion. White arrows, small αS-sfGFP aggregates; pink arrowheads, sequestered lipid accumulations; pink arrow, lipid aggregate partially internalized into the inclusion. (E) pS129 and neurofilament IF in sporadic PD brain (frontal cortex) shows soma-type inclusion reminiscent of fusion examples in (B)–(D). (F) CLEM example of αS(+) inclusions with mixed amyloid and lipid pathology in substantia nigra of sporadic PD brain (top) and seeded inclusionopathy model (bottom). (G) Manual longitudinal single-inclusion and single-cell survival tracking. Log rank test: ****p < 0.0001. Error bars = SD.

Given the reduction in membrane-rich inclusions upon PFF seeding, we tested whether these inclusions were truly dynamic. Lipid-rich inclusions can dissipate in the presence of trifluoperazine (TFP)^57^ or nortriptyline (NOR)^58^ at high doses. While such doses are toxic in our pi-N^A53T-sfGFP-pB^ model after multiple days of exposure (Figure S5G), both compounds selectively abrogated lipid-rich (Type I) inclusions within minutes in a dose-dependent manner (Figures 5A and S5F; Videos S1 and S2). However, these tool compounds had no effect on seeded fibrillar neurite-type or ribbon (putative Type II) soma-type inclusions, suggesting a fundamentally different biology among these inclusion subtypes.

### Intraneuronal fusion events between inclusion subtypes impact neuronal survival

In synucleinopathy brain, the mechanism through which membrane-rich PBs and fibril-rich LBs form is unknown: do they represent different stages of inclusion formation, a dynamic progression from one form to another, or distinct inclusions that form in parallel?^8^ We explored this in our pi-N^A53T-pB^ model.

We examined inclusion dynamics with single-cell longitudinal tracking. Unexpectedly, we detected intraneuronal fusion events between Type I lipid-rich (LipidSpot(+)) inclusions and Type II (LipidSpot(−)) inclusions. In one common scenario, movement of a Type II fibrillar LipidSpot(−) neurite-type inclusion toward the cell soma resulted in dispersal of LipidSpot(+) signal that was present in the soma (Figure 5B; Video S3). Neurons containing both Type I LipidSpot(+) (white arrows) and Type II LipidSpot(−) (blue arrowheads) inclusions were also detected by confocal microscopy (Figure 5C). 4D dynamic lattice-sheet microscopy (x, y, z, time) enabled visualization of neurite-type inclusions protruding into lipid-rich structures in the cell, wherein αS-sfGFP and LipidSpot(+) fragments directly apposed each other, signifying an apparent fusion event (Figure 5D; Video S4). In sporadic PD brain, some neurite-type inclusions (pS129(+) and neurofilament(+)) appeared continuous with pS129(+) inclusions in the soma (Figure 5E). Furthermore, CLEM demonstrated dense filamentous material immediately adjacent to vesicular structures in both sporadic PD brain and the seeded inclusion model (Figure 5F), consistent with prior observations that membrane-rich and fibril-rich αS pathologies are not mutually exclusive in postmortem brain.^55^

To examine the biological effect of fusion events, we conducted manual single-inclusion survival tracking. The pi-N^A53T-sfGFP-pB^ line was seeded with PFFs and stained with LipidSpot, and inclusions were then tracked for ~2 weeks (Figure 5G, top) from DIV21, a time point at which most inclusions are p62(−) (see Figure 4C). Both classes of soma-type inclusions (Type I LipidSpot(+) and Type II LipidSpot(−)) conferred lower neuron survival probability compared with neurons that did not develop inclusions at all (inclusion(−)) (Figure 5G, bottom), confirming that distinct somatic inclusion subtypes are neurotoxic. Surprisingly, neurons that contained Type I LipidSpot(+) inclusions at the start of tracking but then converted to Type II LipidSpot(−) (converter) as a result of fusion had improved survival. These data suggest that Type I lipid-rich inclusions are neurotoxic and that intraneuronal fusion events with Type II fibril-rich inclusions lead to their detoxification.

### Longitudinal single-cell tracking reveals that lipid-rich (Type I) αS inclusions are neurotoxic

Amplification of E46>K familial PD mutation within three imperfect repeats in the αS N-terminal helix (E35>K+E46>K+E61>K [3K]) enhances membrane binding of αS.^27,59^ A transgenic 3K mouse model recapitulates PD cellular pathologies and clinical manifestations such as levodopa-responsive tremor.^60^ Lentiviral 3K expression in neuroblastoma cells induces membrane-rich inclusions.^59,61^ We thus hypothesized that this mutant would spontaneously generate pure Type I inclusions in human cortical neurons without need for exogenous seeding.

**Figure 6.**
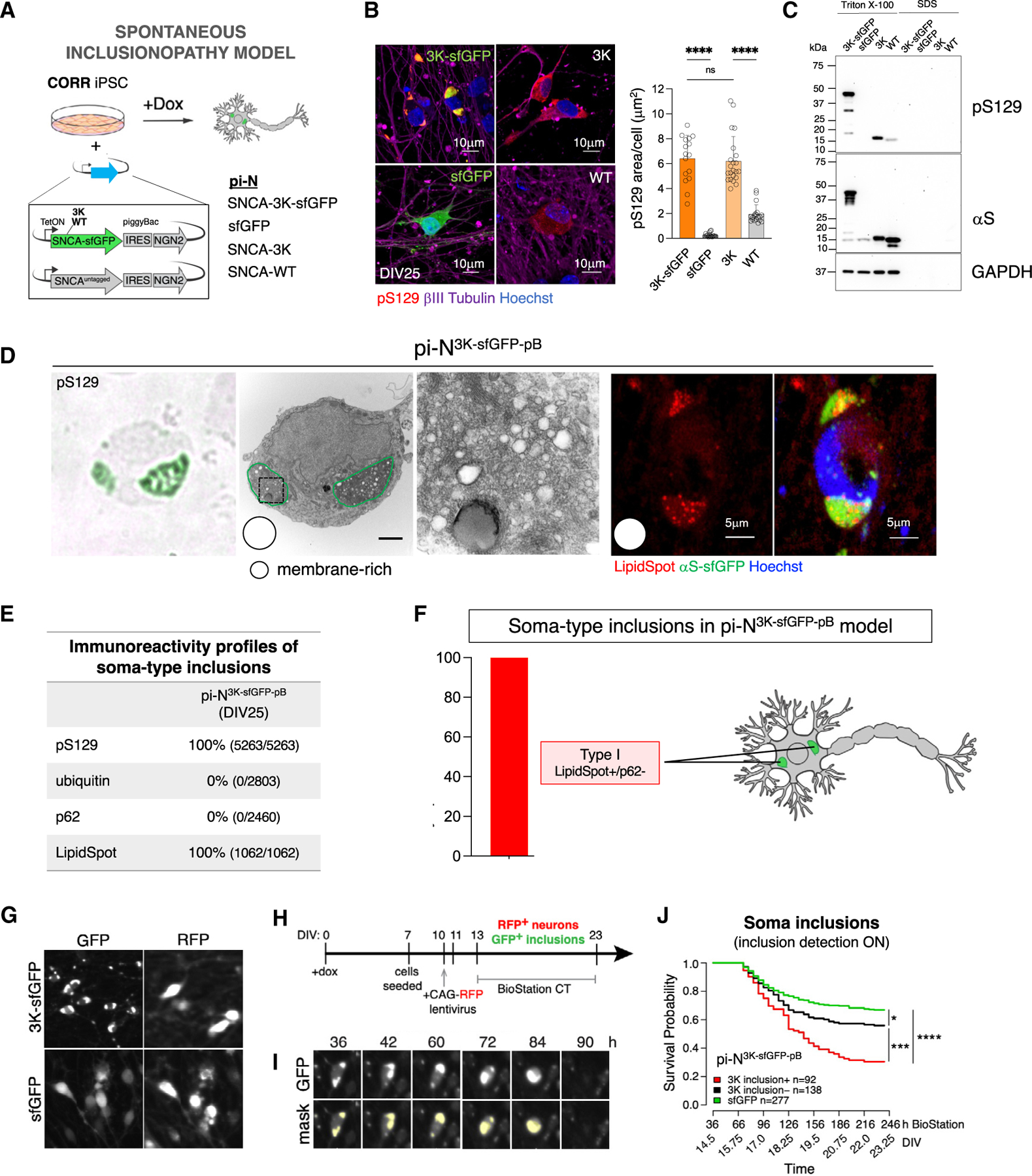
A spontaneous aggregation model recapitulates features of lipid-rich inclusions in seeded inclusionopathy model (A) Schematic of spontaneous inclusionopathy model. (B) Left, pS129 IF in pi-Ns overexpressing sfGFP-tagged or untagged αS-3K, untagged αS-WT, or sfGFP control. Right, quantification of IF. One-way ANOVA plus Tukey’s multiple comparison test: ****p < 0.0001; ns, not significant. (C) Western blot after sequential TX-100/SDS extraction of soluble and insoluble protein fractions. (D) CLEM for αS-pS129 (leftmost 3 panels) and LipidSpot labeling (right 2 panels) in spontaneous inclusionopathy model. (E) Immunoreactivity profiles of soma-type inclusions in pi-N^3K-sfGFP-pB^ model. Total number of inclusions counted shown in parentheses. (F) Inclusion subtype classification in spontaneous inclusionopathy model. (G) Inclusion survival tracking in spontaneous inclusionopathy model. Examples of GFP(+)/RFP(+) neurons detected in the BioStation CT. (H) Experimental time line for BioStation CT imaging. (I) Example of automated mask for soma-type inclusions. Neuron was identified as dead at the 90 h time point. (J) Kaplan-Meier curve comparing survival probabilities of pi-N^3K-sfGFP-pB^ model with and without inclusions and pi-N^sfGFP-pB^ control neurons. Log rank test: *p < 0.05, ***p < 0.001, ****p < 0.0001. Data are representative of 3 separate neuronal differentiations. Error bars in (B) = SD.

We created an αS-3K pi-N model with our all-in-one pB in the CORR iPSC background (pi-N^3K-pB^) (Figure 6A). pS129(+) inclusions indeed formed spontaneously in sfGFP-tagged and untagged models (Figure 6B) and did not require the NAC domain (Figure S9A, right), affirming fundamental biophysical differences with seeded inclusions. Sequential TX-100/SDS extraction at DIV25 confirmed that αS-3K is largely soluble (Figure 6C). pi-N^3K-sfGFP-pB^ inclusions were only of the vesicle- and lipid-rich class and were LipidSpot(+) (Figures 6D and 6E). In addition, all inclusions assessed at DIV25 were consistently negative for ubiquitin and p62, also indicative of pure Type I inclusions (Figures 6E, 6F, and S6A). Thus, this model offered a unique opportunity to study the impact of one specific subset of inclusions on neuronal survival.^62^

We developed algorithms for single-cell inclusion survival tracking with longitudinal imaging (BioStation CT, Nikon) for both spontaneous and seeded inclusionopathy models (Figures S6B–S6D). The algorithms input time-lapse images of live neuronal cultures and automate detection of neurons, inclusions, and live/dead status based on fluorescence intensity and size. For the seeded inclusion model, single-cell inclusion survival tracking is based only on soma-type inclusions; neurite-type inclusions are tracked on a population basis because of challenges in linking neurite-type inclusions to corresponding cell bodies (Figures S7A and S7B).

To investigate survival, we tracked single neurons longitudinally in the spontaneous inclusion model (Figures 6G–6I). pi-N^3K-sfGFP-pB^ models exhibited higher risk of death than control pi-N^sfGFP-pB^ models (Figure 6J). Among pi-N^3K-sfGFP-pB^ models, those neurons with inclusions at the start of tracking had a higher risk of death than neurons that never developed inclusions (Figure 6J). Thus, αS-3K Type I inclusions confer toxicity to neurons, recapitulating manual-tracking results (Figure 5G).

We also conducted longitudinal single-cell tracking in the seeded inclusion model (Figure S7C). pi-N^A53T-sfGFP-pB^ models had lower survival probability than pi-N^ΔNAC-sfGFP-pB^ models (Figure S7D). Seeding with PFFs conferred a similar level of toxicity in both A53T and the aggregation-dead mutant ΔNAC, suggesting that PFFs result in aggregation-independent toxicity, though we cannot rule out this toxicity was partially due to seeding of endogenous αS in the CORR line (Figures 2B and 2C).

The inclusion survival tracking algorithm does not distinguish between Types I and II inclusions and instead interrogates survival status of all neurons with GFP(+) inclusions in the soma. We found that inclusion-bearing pi-N^A53T-sfGFP-pB+PFF^ bearing inclusions at early time points (when most inclusions are p62(−); see Figure 4C) exhibited a higher risk of death than neurons that never developed inclusions (Figure S7E). Cumulative length of neurite-type inclusions increased with time in seeded pi-N^A53T-sfGFP-pB+PFF^ (Figure S7F). However, despite the abundance of such inclusions, the seeded toxicity in A53T neurons was not detectably higher than in ΔNAC neurons (Figures S7D and S7F). Thus, in this model system, neurite-type inclusions may not be intrinsically toxic to neurons, whereas soma-type inclusions are. Among the latter, Type I inclusions are toxic, as demonstrated in both the 3K model (Figure 6J) and the manual tracking of fusion events (Figure 5G). Type IIa p62(+)/LipidSpot(−) inclusions are likely protective, as suggested by our cross-sectional analyses (Figure 4F), whereas Type IIb p62(−)/LipidSpot(−) inclusions are likely toxic.

Thus, our automated algorithms can accurately track survival at single-cell and single-inclusion resolution, revealing that specific subtypes of inclusions within the soma, rather than neurites, may be particularly toxic to human neurons, at least within the time frame examined.

### αS-protein interaction analyses pinpoint proteins sequestered in membrane-rich inclusions

**Figure 7.**
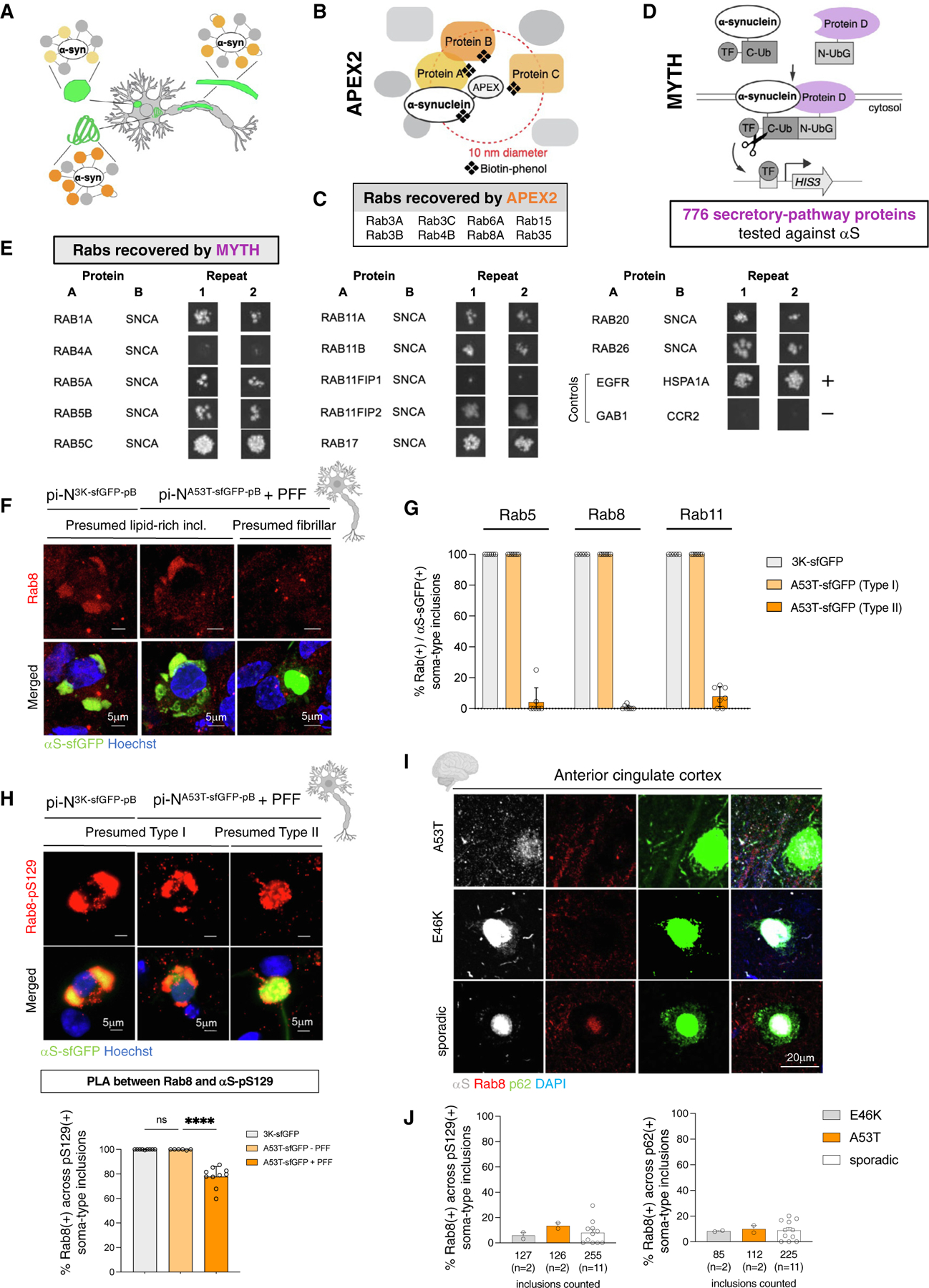
Proximity labeling and membrane two-hybrid assay as tools for identifying proteins sequestered in membrane-rich inclusions (A) Diagram of αS protein-protein interactions within different inclusion subtypes. (B) Cartoon of αS-APEX2 proximity labeling. (C) Rab proteins found in the vicinity of αS by APEX2.^46^ (D) Schematic of membrane yeast two-hybrid (MYTH) assay. (E) Rab-αS protein interaction by MYTH (2 replicate experiments). (F) Rab8 IF in seeded and spontaneous inclusionopathy models. (G) Quantification of Rab(+)/pS129(+) inclusions in seeded and spontaneous inclusionopathy models. (H) *In situ* detection of Rab8 and pS129 interaction by proximity ligation assay (PLA). Bottom, quantification of pS129(+)/Rab8(+) soma-type inclusions. One-way ANOVA plus Tukey’s multiple comparison test: ****p < 0.0001; n.s., not significant. (I) IF for Rab8, p62, and αS in familial A53T and E46K (*n* = 2 each) and sporadic PD (*n* = 11) brain. (J) Left, quantification of pS129(+)/Rab8(+) inclusions in PD brain. Right, quantification of (I). Experimental replicates: 3 (G) and 3–4 (H) independent replicates each across 3 separate neuronal differentiations. Error bars = SD.

The distinct biological impact of inclusion subtypes may relate to sequestration, redistribution, or destabilization of different proteins, lipids, and metabolites as a consequence of inclusion formation (Figure 7A).^63^ Because Type I lipid-/membrane-rich αS inclusions (Figure 4K) were clearly toxic (Figure 6J), we focused on identifying proteins sequestered into this class of inclusions and exploited αS-protein interaction mapping to narrow down potential targets.

We previously reported 255 proteins in the immediate vicinity of ascorbate peroxidase (APEX2)-tagged αS in primary rat cortical neurons (Figure 7B).^46^ APEX2 hits included proteins related to endocytic vesicle trafficking, actin stabilization, retromer complex, synaptic processes, and mRNA metabolism. Among vesicle trafficking proteins, Rab family members were prominent (Figure 7C).^46^ To identify proteins directly complexed to αS, we turned to a binary interaction assay known as membrane yeast two-hybrid (MYTH)^64^ (Figure 7D). In total, 776 proteins in the secretory pathway were tested for interactions with αS. Overall, 12 Rab proteins interacted with αS by MYTH (Figure 7E). Notably, Rab proteins were a strong class of hits in both APEX2 and MYTH screens (8/32 Rabs identified by mass spectrometry were APEX2 hits; 12/15 Rabs tested were hits by MYTH). We prioritized Rab hits from APEX2 (Rab5 and Rab8) and MYTH (Rab11) with good available antibodies for further analysis.

Type I lipid-rich inclusions in pi-N^3K-pB^ or seeded pi-N^A53T-pB+PFF^ stained positive for Rab5, Rab8, and Rab11 (“3K-sfGFP” and “A53T-sfGFP” in Figures 7G and S8A, top). Inclusions that were fibrillar based on ribbon-like morphology (“Presumed Fibrillar” or “Type II” in Figures 7F and 7G) were Rab immunonegative. Rab35 (originally recovered in APEX2) gave similar results (data not shown). Importantly, another Rab, Rab7, implicated in PD but not recovered in either of our interaction assays, did not colocalize with any αS inclusion (data not shown). Labeling of these Rab proteins was diffuse in control neurons without inclusions (Figure S8A, bottom). Thus, Rab proteins with roles in different membrane-trafficking compartments interact with αS and are enriched within lipid-rich inclusions.

The proximity ligation assay (PLA) utilizes oligonucleotide-hybridized antibodies to detect close protein-protein interactions more sensitively than immunofluorescence *in situ*.^27,64^ All Type I inclusions demonstrated αS (pS129)-Rab8 interaction in both pi-N^3K-sfGFP-pB^ (100%, *n* = 2267) and unseeded pi-N^A53T-sfGFP-pB^ (99.88% ± 0.3% [SD], *n* = 631) models (Figure 7H, bottom). Surprisingly, PLA also detected αS (pS129)-Rab8 interaction, albeit less uniformly, in presumed Type II fibrillar inclusions (77.85% ± 7.8% [SD], *n* = 1666) (Figure 7H). PLA signal was not detected without primary antibodies or in a single-primary-antibody PLA control (Figure S8B) and was only sparsely detected in control neurons (Figure S8C).

Since lipid-rich (Type I) and presumed fibrillar lipid-negative (Type II) inclusions differentially colabeled with Rab8 in our pi-N models (Figures 7F–7H), we asked if this was also the case in human brain. While classic PBs and LBs are identifiable with H&E and pS129 staining in the substantia nigra, these dichotomous inclusion types may also present in the cortex, albeit far more subtly. Rab8 colocalized with a subset of pS129(+) or p62(+) inclusions in familial A53T, E46K, or sporadic PD postmortem brains (Figures 7I and 7J, right). Notably, the frequency of pS129(+)/Rab8(+) cytoplasmic inclusions in matched patient brain (A53T) was lower (13.61% ± 2.3% [SD], *n* = 126) than in our neuronal models and even lower in E46K (5.28% ± 3.1% [SD], *n* = 127) and sporadic brains (7.94% ± 8.8% [SD], *n* = 255) (Figure 7J, left). This could reflect decreased survival of neurons harboring such inclusions in end-stage brain. Consistent with this, Rab8 colocalized with inclusions staining positive for the neutral lipid marker BODIPY in formalin-fixed paraffin-embedded (FFPE) sections of A53T familial and sporadic PD brains (Figure S8D), as predicted by our models (Figure 7H). Notably, beyond optimizing deparaffinization (see STAR Methods), we also directly confirmed that two different neutral lipid dyes, BODIPY and Nile Red, avidly stained lipids in formalin-fixed frozen cryostat (FFFC) mouse white adipose positive-control tissue (Figure S8E) as well as in immediately adjacent FFFC and FFPE sections from the same human postmortem brain (Figure S8F). We then confirmed that both lipid dyes marked a subset of αS-immunopositive inclusions in FFFC brain tissue from A53T (*n* = 2) and sporadic PD (*n* = 4) cases (Figure S8G).

Thus, protein-interaction mapping can uncover markers that label specific inclusion subtypes in our pi-N inclusion models and human postmortem brain, added to information provided by a generic marker of αS inclusions like pS129.

### Convergence of genetic and protein-protein αS interaction analyses identify RhoA-positive inclusions in postmortem brain

**Figure 8.**
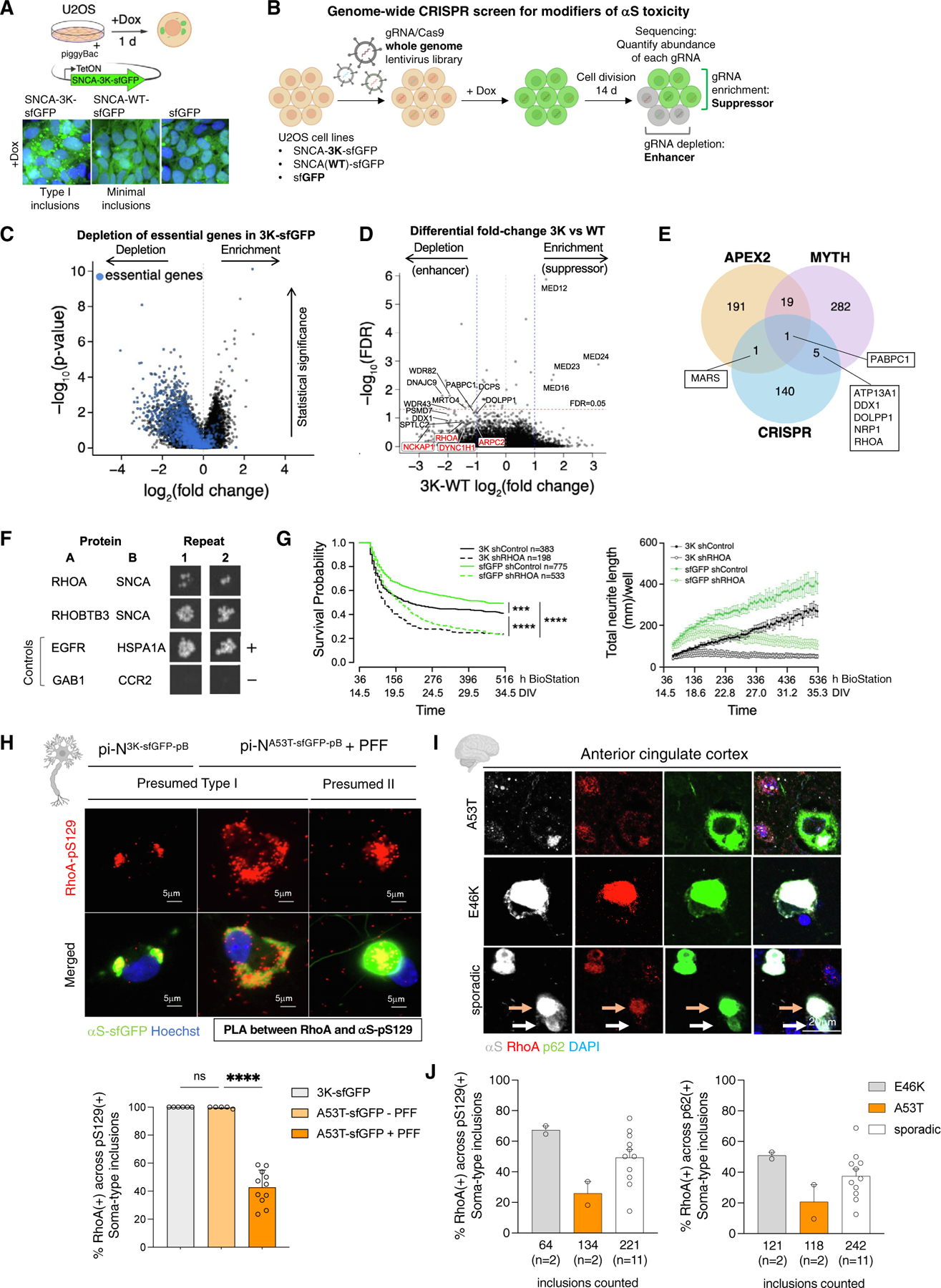
Convergence of CRISPR screen and MYTH on cytoskeleton regulators leads to identification of RhoA(+) inclusions in postmortem brain (A) Cartoon of U2OS model harboring pB-SNCA-3K-sfGFP transgene. Micrographs show transgene GFP signal in doxycycline-treated cells. (B) Genome-wide CRISPR-Cas9 knockout screen for modifiers of αS toxicity. (C) Volcano plot showing depletion of essential genes (blue).^67^ (D) Volcano plot comparing fold-change differential between SNCA-3K-sfGFP and SNCA-WT-sfGFP genotypes. (E) Overlap between spatial (APEX2 and MYTH) and genetic (CRISPR) screen hits. (F) Interaction of actin cytoskeleton-related proteins RhoA and RhoBTB3 with αS by MYTH. (G) Left, Kaplan-Meier curve of single-cell survival tracking in pi-N^3K-sfGFP-pB^ and pi-N^sfGFP-pB^ models transduced with shRNA lentivirus. Log rank test: ***p < 0.001, ****p < 0.0001. Data are representative of 2 neuronal differentiations with shRNA lentivirus at MOI20. Right, neurite measurement based on RFP signal. (H) PLA of RhoA-pS129 in inclusionopathy models. Bottom, quantification of pS129(+) soma-type inclusions from 3 to 4 independent replicates across 3 separate neuronal differentiations. One-way ANOVA plus Tukey’s multiple comparison test: ****p < 0.0001; n.s., not significant. (I) IF for RhoA, p62, and αS in A53T (*n* = 2), E46K (*n* = 2), and sporadic (*n* = 11) PD brain. Orange arrow, αS(+)/p62(+)/RhoA(+) inclusion; white arrow, αS(+)/p62(+)/RhoA(−) inclusion. (J) Left, quantification of pS129(+)/RhoA(+) inclusions in PD brain. Right, quantification of (I). Error bars = SD.

Some proteins sequestered into αS inclusions may contribute to neurodegeneration, while others may simply be bystanders. We thus asked which αS-interacting proteins found in the toxic Type I lipid-rich inclusions lead to lethality when depleted in the presence of 3K inclusions but not with equivalent levels of WT αS. We performed a CRISPR-Cas9-based genome-scale deletion screen in human cells.^65,66^ The pB system was adapted easily to cell lines, generating discovery models amenable to high-throughput and genome-wide genetic screening (requiring ~180 million cells). A pB U2OS cell model expressing SNCA-3K-sfGFP along with equivalent levels of SNCA-WT-sfGFP and sfGFP in control lines was made (Figures 8A and S9A, left). The 3K model formed inclusions with concomitant cytotoxicity (or reduced growth) upon doxycycline induction, whereas few inclusions or toxicity were detected with WT expression (Figures 8A and S9A, center). In contrast to A53T inclusions, 3K inclusions formed independent of the NAC domain (Figures S9A and S9B).

U2OS 3K and control cells were transduced with a ~90,000 single guide RNA (sgRNA)/Cas9 lentivirus library for genome-wide knockout screening, followed by doxycycline induction of transgene expression (Figure 8B). Cells were harvested at 0, 7, and 14 days post-induction and processed for next-generation sequencing. Depletion of guide RNAs (gRNAs) relative to t = 0 indicates genes that enhance toxicity (or reduce growth) when knocked out, whereas enriched gRNAs indicate suppressors. Depletion of essential genes confirmed that the screening pipeline was effective (Figure 8C).

Genes selectively toxic to 3K versus WT cells when knocked out (Figures 8D and S9C) were enriched in Gene Ontology (GO) PANTHER pathways such as positive regulation of cytoskeleton organization (FDR = 2.72×10^−2^), RNA metabolic process (false discovery rate [FDR] = 8.11 × 10^−8^), ribosome biogenesis (FDR = 1.34 × 10^−7^), and protein metabolic process (FDR = 7.81 × 10^−3^) (Figures S9D and S9E; Tables S3 and S4). Genes encoding cytoskeleton regulators (*ARPC2*, *DYNC1H1*, *NCKAP1*, and *RHOA*) were recovered in the screen as enhancers of *SNCA* toxicity, as were RNA processing genes (*DCPS*, *WDR82*, *MRTO4*, *DDX1*, and *DDX49*), two classes of genes previously tied to αS toxicity.^27,68^ Enhancers of 3K toxicity also included genes relating to protein misfolding, protein aggregation, and lipid posttranslational modifications: heat shock protein family members (*DNAJC2* and *DNAJC9*), proteasome-related genes (*PSMD7* and *PSMG2*), prefoldin subunit (*PDRG1*),^69^ and a palmitoyltransferase (*SPTLC2*).^70^ We compared top hits in this CRISPR screen to top hits in our αS MYTH assay. There were 6 overlapping genes (Figure 8E). One, *PABPC1*, was also a top hit in the APEX2 screen and was previously discovered as a genetic modifier of αS toxicity that is translationally dysregulated in αS mutant neurons.^27,46^

Our attention was drawn to RhoA. RhoA is a major regulator of actin stabilization. As a class, regulators of actin stability were enriched in both our CRISPR and prior αS-APEX2 screens. Moreover, *RHOA* and *RHOBTB3* also physically interact with αS, as indicated by MYTH (Figure 8F). Sequestration of RhoA and other cytoskeletal factors could be neurotoxic as αS inclusions form. Treatment with short hairpin RNA (shRNA)-*RHOA* lentivirus resulted in dose- and time-dependent knockdown of the protein and unbranched neurites in the pi-N^3K-sfGFP-pB^ line (Figures S10C and S10D). *RHOA* knockdown was highly toxic in cortical neurons (Figure 8G, left). Total neurite length, a proxy for neuronal health, was reduced in both pi-N^3K-sfGFP-pB^ and pi-N^sfGFP-pB^ models (Figure 8G, right).

Our data suggested that sequestration of RhoA in inclusions could be neurotoxic by mislocalizing the protein from its physiologic active site in neurites. We thus examined whether RhoA’s subcellular localization was altered in the presence of 3K inclusions compared with other top hits from our CRISPR screen. While ArpC2 redistributed from punctate (control neurons, pi-N^sfGFP-pB^) to diffuse staining pattern in the pi-N^3K-sfGFP-pB^ model (Figure S10A), it did not colocalize with inclusions. PABPC1 did not colocalize, either (Figure S10B). By contrast, RhoA strongly colocalized with Type I inclusions in the 3K and unseeded A53T models (pi-N^3K-pB^: 100%, *n* = 1,198; unseeded pi-N^A53T-pB^: 99.72% ± 0.6% [SD], *n* = 338) and to a lesser extent in the PFF-seeded A53T model (42.82% ± 11.6% [SD], *n* = 1,331) by PLA (Figures 8H, S10G, and S10H, controls). Only few (0.88% ± 1.8% [SD], *n* = 647) inclusions exhibited a RhoA-p62 PLA signal, lending support to a direct αS (pS129)-RhoA interaction that occurs only within an inclusion subset (Figure S10F).

Moving to postmortem brain, RhoA also marked a subset of lipid-rich (BODIPY(+)) inclusions in familial A53T and sporadic PD patient brains (Figure S10I). In A53T brains, just as in our PFF model, we detected occasional colocalization between RhoA and pS129(+) (25.92% ± 7.7% [SD], *n* = 134), with higher colocalization rates observed in the E46K (49.25% ± 16.6% [SD], *n* = 64) and sporadic PD (67.35% ± 2.7% [SD], *n* = 221) brains (Figure 8J, left). RhoA/p62 colocalization was similar (Figures 8I and 8J, right). These data demonstrate that a subset of RhoA(+) inclusions exist in synucleinopathy brain. Our pB models thus enabled discovery of novel inclusion subtypes in the brain that are rich in RhoA, a protein that may be neurotoxic when sequestered.

To establish whether our screens identified genes and proteins relevant to synucleinopathy more broadly, we turned to the Religious Orders Study and Memory and Aging Project (ROS/MAP). ROS/MAP is a population-based study with detailed measures of postmortem neuropathology that can be directly related to clinical and molecular phenotypes. We analyzed mRNA abundance in dorsolateral prefrontal cortex (DLPFC) of 1,011 brains.^71^ DLPFC is matched to our iPSC cortical neuron model and is also a region with relatively early PD pathology, which avoids confounding end-stage neuronal and glial responses. We asked whether transcriptional changes in our top hits were altered in response to αS accumulation, as measured by LB staging. We detected significant enrichment of MYTH (*n*_genes_ = 269; *p* = 0.01) and combined MYTH/CRISPR screen (*n*_genes_ = 401; *p* = 0.0066) gene sets with LB stage (Figure S10J). Enrichments were in the positive direction with LB stage, indicating increasing dysregulation as LB pathology advances.

## DISCUSSION

A linear “monomer to oligomer to amyloid” model of proteinaceous aggregation neglects the conformational, ultrastructural, and spatial heterogeneity of inclusions in neurodegenerative diseases. Equally, this heterogeneity eludes common neuropathologic markers (e.g., αS-pS129 or phospho-tau). Moreover, the end-stage “snapshot” in postmortem studies does not capture the cause and consequence of these inclusions. Here, we have developed a set of tractable, reproducible, and readily transferable human stem cell-based models to fill these gaps.

We focused here on cortical αS inclusionopathy models. Our investigations revealed that, when αS reaches high concentrations in cortical neurons, lipid-rich and Triton-extractable pS129(+) inclusions form (Type I; Figures 4J and 4K). This fundamental behavior is greatly facilitated by E→K αS mutations (Figures 6B–6F), just as in cell lines^59,61^ and mice.^60^ Parallels trace back to organisms like yeast that die when αS forms inclusions that stall vesicle trafficking.^28,72–74^ Notably, genetic modifiers of αS toxicity in yeast comprise multiple membrane-trafficking genes that are known PD genetic risk factors.^75–77^ This lipid-rich inclusion subtype is also very toxic (Figure 6J) and highly dynamic, capable of being “dissolved” within minutes of exposure to NOR and TFP (Figures 5A and S5F), drugs that protect against αS toxicity at lower doses.^58^ We have previously shown that preventing the formation of these types of inclusions with stearoyl-CoA desaturase inhibitors is neuroprotective in cellular and animal models.^78–82^

By contrast, exogenous seeding with αS induces inclusions that are SDS extractable but Triton insoluble. These are pS129(+), neutral lipid negative, and p62(+) (Type IIa) or p62(−) (Type IIb) (Figures 3D–3F and 4K). While Type IIb inclusions are also toxic (Figures 5G and S7E), Type IIa inclusions are neuroprotective (Figures 4F and S4K) and can detoxify lipid-rich inclusions by fusing with them (Figures 5B and 5G; Video S3). Such fusion events could explain how PBs and LBs coexist within single neurons.^8^ Alternatively, PB may also evolve into a more fibrillar inclusion (or vice versa), as noted in a mouse (3K) model^60^ and in a rodent PFF culture model.^18^ The transition from lipid-rich to amyloidogenic self-amplifying assembly needs further investigation, and numerous existing tools may be useful for doing this.^83^

Types I and II αS inclusions are found in postmortem brain. While dichotomous fibril- versus lipid-rich αS inclusions are more obvious in substantia nigra dopaminergic neurons, our study suggests that this dichotomy is just as strong ultrastructurally in cortical neurons. We also identified bystander proteins sequestered within inclusions. Combining prior αS APEX2 proximity labeling^46^ and MYTH assays^46,84^ in this study, we narrowed in on secretory pathway proteins that colocalized with Type I inclusions in our models (Rabs 5, 8, and 11) (Figure 7G) and in postmortem brain (Rab8) (Figures 7I and 7J). We performed a genome-wide CRISPR-Cas9 screen to identify proteins that lead to neurotoxicity in inclusion-bearing neurons when deleted. RNA-processing and actin-cytoskeleton modulators emerged as major classes of genes that, when knocked out, led to specific dropout of inclusion-bearing cells. These pathways are already implicated in synucleinopathy.^27,68,85^ One of these hits, the cytoskeletal regulator RhoA, labels a subset of inclusions in synucleinopathy brains (Figures 8I and 8J). RhoA depletion was toxic to neurons (Figure 8G) and perhaps too neurotoxic at the levels of knockdown and early time points we tested to distinguish inclusion-bearing (αS-3K) from control neurons. We speculate that RhoA regulation in polarized neurons may be more complex than in cell lines. Deletion of GTPase-activating proteins of RhoA can have opposing effects, suggesting tight regulation in neurons.^86^ Our data caution that a nuanced understanding of RhoA-ROCK inhibition is required before adoption as a PD therapeutic target.^87,88^ These data are reminiscent of AD and AD mouse models in which RhoA activity decreases with pathology progression, a phenomenon associated with neuritic dystrophy and RhoA sequestration within neurofibrillary tangles.^89^

Altogether, our data suggest that neurodegenerative proteinopathies should be viewed as “polyproteinopathies” in which the misfolding of one protein like αS, tau, or TDP-43 leads to a multitude of misfolding, redistribution, and protein-sequestration events. We speculate that these play a role in differential vulnerability of specific cell types and heterogeneity of responses among patients. Beyond proteins, it will also be important to analyze our models for non-proteinaceous components that can be sequestered into fibrils.^5^

### Limitations of the study

Limitations of our models include (1) use in some models of doxycycline, a mitochondrial^90^ and αS aggregation^91^ modulator that could impede analysis of mitochondrial and lysosomal pathologies (Figures S3C–S3G); (2) developmentally immature neurons—future studies should concomitantly accelerate both maturation^92^ and aging^93–95^ “in the dish”; (3) random integration of pB transgene; (4) potential αS-independent PFF toxicity (Figure S7D; preliminary results in the pi-N^SNCA−0-copy^ line support this possibility [data not shown]); (5) reliance on fluorescence intensity to assess cell survival (Figure S6B), which may be improvable with phase contrast morphology or direct cell-death indicators^96^; and (6) absence of glial co-culture,^97^ leaving open the possibility that lack of PFF-induced toxicity in our neurons compared to *in vivo*^37,98,99^ may relate to a lack of inflammatory responses.^100–102^

We combined different screening systems (U2OS cells, CRISPR; neurons, APEX2; yeast, MYTH) as discovery tools. In future investigations, these analyses should be performed in the same system.

Finally, the induced astrocytes presented here mature rapidly, albeit at the expense of poor expandability. Alternative transcription factors may offer superior scalability but less rapid maturation.^103^ Certain non-cell-autonomous effects may only be recapitulated in 3D sphere/organoid^104^ and human-mouse chimeric systems.^105^ We envisage that our models will also aid these efforts.

### Conclusions

We anticipate that the rapid and scalable iPSC inclusionopathy models like those described here will contribute to molecular-level understanding of inclusion subclasses and their distinct biological consequences. They will enable systematic mapping of genetic and physical interactors of different αS conformers (or “strains”) in distinct CNS cell types and co-cultures and also facilitate modeling of mixed proteinaceous pathologies. These models now offer a path to a personalized model incorporating both patient-specific CNS cells and proteinaceous strains amplifiable from patient tissue and body fluids.^106,107^ Candidate diagnostic agents like radiotracers or therapeutic agents like antibodies and small molecules can now in principle be tested in stem-cell models derived from individual patients.

## STAR★METHODS

### RESOURCE AVAILABILITY

#### Lead contact

Further information and requests for resources and reagents should be directed to and will be fulfilled by the lead contact, Vikram Khurana (khuranalab_admin@bwh.harvard.edu).

#### Materials availability

All unique/stable reagents generated in this study are available from the lead contact with a completed materials transfer agreement. Key plasmids have been deposited at Addgene (Table S1).

#### Data and code availability

- A comprehensive key resources table listing all research outputs can be found at Zenodo: https://doi.org/10.5281/zenodo.12549027. All tabular data can be found at Zenodo: https://doi.org/10.5281/zenodo.12549027, and code relating to figures can be found at Zenodo: https://doi.org/10.5281/zenodo.12574231. The U2OS CRISPR-Cas9 genetic screen data (raw counts and analysis files) are available at Mendeley Data (https://data.mendeley.com) at the DOIs listed in the key resources table.
- Original code relating to the Nikon BioStation CT survival algorithms is available at Zenodo at the DOIs listed in the key resources table.
- All data reported in this paper will be shared by the lead contact upon request.
- Any additional information required to reanalyze the data reported in this paper is available from the lead contact upon request.

### METHOD DETAILS

#### Molecular cloning

For Gateway cloning, gene blocks (double-stranded DNA fragments) and primers were purchased from IDT (Integrated DNA Technologies). LR and BP clonase mix were used per recommended protocol from supplier for Gateway cloning (Gateway LR Clonase II Enzyme mix, ThermoFisher 11791100; Gateway BP Clonase II Enzyme mix, ThermoFisher 11789100). Donor or destination plasmids containing *ccdB* sequence were propagated in *ccdB*-resistant *E. coli* strain One Shot *ccdB* Survival 2 T1^R^ Competent Cells (Life Technologies A10460). Expression clones were transformed into 10-beta competent *E. coli* (New England Biolabs C3019).

#### Generation of targeted inducible transgene at *AAVS1* locus in hESC via TALENs

To establish Tet-On system transgene at the *AAVS1* locus within the *PPP1R12C* gene, two rounds of TALEN-mediated gene editing were conducted in hESC lines (male WIBR-1, clone 22, or female WIBR-3, clone 38). First, one construct containing the M2rtTA reverse tetracycline transactivator under the control of the constitutive CAGGS promoter (P_CAGGS_-M2rtTA) was targeted to one *AAVS1* allele. The second *AAVS1* allele was subsequently targeted with a construct containing the transgene of interest driven by the M2rtTA-responsive TRE-Tight promoter (e.g., P_TRE-Tight_-SNCA-mK2). Both constructs have flanking 5′ *AAVS1* and 3′ *AAVS1* homology arms.

#### Southern blotting

Correct integration of the Tet-On constructs at the *AAVS1* locus within the *PPP1R12C* gene was confirmed by Southern blot analysis. An *AAVS1* internal 5′-probe, corresponding to the 5′ homology arm of the *AAVS1* donor targeting vector, was used to detect extra integration sites beyond the *AAVS1* locus. An *AAVS1* external 3′-probe, which hybridizes with a sequence downstream of exon 3 of the *PPP1R12C* gene, was used to confirm integrity of the *AAVS1* locus.

Genomic DNA was extracted according to the manufacturer’s instructions (DNeasy Blood and Tissue Kit, Qiagen, 69504) from hESCs harvested from a well of a 12-well plate, at 70–90% confluency. Genomic DNA was digested with EcoRV-HF restriction enzyme according to the manufacturer’s instructions (New England Biolabs, 3195). DNA restriction fragments were size-fractionated by electrophoresis in a 0.8% agarose gel (SeaKem GTG agarose, Lonza 50070) in Tris-acetate-EDTA (TAE) electrophoresis buffer containing 0.5 μg/mL ethidium bromide (Thermo Fisher Scientific, 15585011). The gel was washed for 15 min in 0.25 M HCl solution (nicking buffer) at 80 rpm, followed by 15 min at 80 rpm in 0.4 M NaOH solution (denaturing and transfer buffer), and assembled in a transfer stack for alkaline Southern transfer of the single-stranded DNA fragments onto a nylon membrane (Amersham Hybond-XL, GE Healthcare, RPN2222S). Southern transfer was conducted overnight via upwards capillary action mediated by the transfer buffer. The next day, the transfer membrane was rinsed in 0.2 M Tris-Cl, pH 7.0 and 2X saline sodium citrate (SSC; 0.3 M NaCl with 7.5 mM trisodium citrate), for 2 min each at 80 rpm. The transfer membrane was dried for 15 min in a 55°C oven, followed by a pre-hybridization (blocking) step with hybridization buffer (1% [w/v] bovine serum albumin/BSA, 1 mM ethylenediaminetetraacetic acid/EDTA, 0.5 M NaPO_4_, 7% [w/v] sodium dodecyl sulfate/SDS in deionized water; all Sigma-Aldrich) for 1 h in a 60°C hybridization oven with rotation.

In preparation for radioactive labeling of the *AAVS1* internal 5′-probe, a restriction fragment within the 5′ homology arm was derived by restriction endonuclease digestion of the *AAVS1* donor targeting vector with SacI (New England Biolabs R0156) and EcoRI (New England Biolabs, R101) according to the manufacturer’s instructions. DNA restriction fragments were size-fractionated by electrophoresis in a 1% agarose gel as described above and the 643 bp restriction fragment was recovered after gel excision using silica membrane spin columns according to the manufacturer’s instructions (MinElute gel extraction kit, Qiagen, 28604). DNA concentration was determined with a NanoDrop ND-1000 Spectrophotometer. Radiolabeling of the 5′-probe was carried out by random-sequence oligonucleotide-primed DNA synthesis. A 28.5 μL reaction volume containing 100 ng of the 5′ homology arm fragment and 5 μL of 50 μM random nonamers (Sigma-Aldrich) in Ambion nuclease-free water (Thermo Fisher Scientific AM9916) were incubated for 5 min at 100°C for denaturation into single-stranded DNA. After 5 min on ice, 5 μL 10X NEBuffer 2 (New England Biolabs, B7002S), 5 μL 100 mM 3dNTPs (minus dCTP; Thermo Fisher Scientific), 5 μL of the radioactively labeled nucleotide [α−^32^P]dCTP (PerkinElmer; 10 μCi/μL) and 1.5 μL Klenow fragment of the *E. coli* DNA Polymerase I (New England Biolabs, M0210) were added for a final volume of 50 μL, and incubated for 30 min at 37°C. The reaction was stopped with 50 μL of buffer TE (Qiagen), and the radiolabeled probe DNA was separated from unincorporated dNTPs by gel filtration chromatography using pre-equilibrated CHROMA SPIN columns (Clontech) with centrifugation at 3,500 rpm for 5 min. The double-stranded probe DNA was denatured for 5 min at 100°C.

The transfer membrane was hybridized with the single-stranded 5′-probe DNA, diluted in fresh hybridization buffer, overnight in the 60°C hybridization oven with rotation. After the hybridization step, the DNA blot was washed at low-stringency in 2X SSC with 0.2% (w/v) SDS for 30 min in a gently shaking 60°C water bath. Any remaining nonspecifically bound probe DNA was washed off during a high-stringency wash with 0.2X SSC (0.03 M NaCl with 0.75 mM trisodium citrate) with 0.2% (w/v) SDS for a minimum of 20 min in a 60°C water bath with gentle shaking. The membrane was sealed in Saran wrap, placed between an autoradiography film (Carestream Kodak BioMax MS film, Eastman Kodak) and an intensifying screen (Eastman Kodak), exposed for 24–72 h at −80°C, brought to room temperature, and developed using the Kodak X-OMAT 1000A film processor.

To re-hybridize the DNA blot with an *AAVS1* external 3′-probe, the transfer membrane was rinsed in 0.08 M NaOH solution (stripping buffer) for a minimum of 15 min at room temperature with gentle shaking. The transfer membrane was washed three times for 5 min with 2X SSC. If any radioactive signal was still detectable, the nylon membrane was stripped in 0.4 M NaOH for 30 min at room temperature, with gentle shaking. The transfer membrane was dried in a 55°C oven before the pre-hybridization, hybridization and autoradiography steps were repeated for the external 3′-probe (a gift from the Rudolf Jaenisch laboratory, Whitehead Institute for Biomedical Research) as described above.

The size of the DNA restriction fragments as detected by the *AAVS1* internal 5′- and external 3′-probes was calculated using the SeqBuilder program in the DNASTAR Lasergene Core Suite v12.0.0 based on the EcoRV restriction sites (one within the integrated targeting vector; one each upstream of exon 1 and downstream of exon 3 of the *PPP1R12C* gene).

#### Generation of targeted transgene at *STMN2* locus in hESC via CRISPR-Cas9

*STMN2* is a neuron-specific gene, which allows for relatively neuron-specific expression of the targeted transgene from the *STMN2* locus. Site-specific genome editing via CRISPR-Cas9 was used to insert sequences coding for *SNCA* into endogenous *STMN2* gene locus.

To target the *SNCA*-*GFP* cassette into the *STMN2* locus, a plasmid was generated bearing ~1800 bp of homology surrounding the *STMN2* stop codon. An IRES-SNCA-GFP coding sequence was then cloned into the *STMN2* homologous sequence such that ~900 bp of homology flanked the IRES-SNCA-GFP cassette. An FRT flanked PGK-Neomycin cassette was then cloned between the IRES-SNCA-GFP cassette and the *STMN2* 3′ homology arm. To incorporate the cassette into the *STMN2* locus, 800,000 H9 hES cells were nucleofected using the Amaxa P3 Primary Cell 4D-Nucleofector X Kit with program CA137. The nucleofection reaction contained 15 μg of sgRNA (5′-tgtctggctgaagcaaggga-3′), 20 μg of ThermoFisher Truecut Cas9 v2 protein and 5.5 μg of the *STMN2* targeting plasmid. After the nucleofection, cells were plated in a 1:1 mixture of StemFlex (Gibco, A3349401) and MEF conditioned StemFlex with Rock inhibitor (Peprotech, 1293823). The cells were allowed to recover for 48 h before G418 selection was initiated. After visible colonies survived the selection, they were picked and plated into a 96-well plate. The expanded cells were replica-plated into two 96-well plates, one of which was used for genotyping. PCR was used to confirm the proper integration of the 5’ (primers STMN2.FOR2 and IRES-REV) and 3’ (primers NEO-F and STMN2-REV1) arms of the targeting cassette into the *STMN2* locus. After targeting confirmation, a clone was expanded and a CAG-FLPo-Puro cassette was nucleofected into the cells following the above protocol. Puromycin selection allowed for the identification of cells which expressed FLP recombinase and colonies derived from these cells were picked, expanded, and genotyped by PCR (primers STMN2.FOR2 and STMN2-REV1) to confirm removal of the PGK-Neo cassette.

#### Quantitative PCR for *STMN2* expression in pi-N^SNCA-STMN2^ neurons

For RNA isolation, DIV21 neurons (see Induced Neuron Differentiation methods) were harvested from 6-well plate cultures by directly applying 1 mL Trizol (ThermoFisher, 15596018) on the cells and slowly shaking them for 10 min at room temperature. 200 μL of chloroform-isoamyl alcohol (Sigma, 25668) was added to 1 mL of Trizol extract and shaken at full speed on a thermoblock for 30 s at room temperature, followed by 15 min 21000*g* centrifugation (table top, 4°C). The resultant aqueous phase (~400–500 μL) was recovered with PureLink RNA mini kit (ThermoFisher, 12183018A) as per manufacturer’s guidelines and final RNA was eluted with 50 μL RNAse Free water. 100 ng of RNA from each sample was reverse transcribed for cDNA production by SuperScript IV VILO Master Mix with ezDNase Enzyme (ThermoFisher, 11766050). Real-time qPCR measurement was performed with TaqMan Fast Advanced Master Mix (ThermoFisher, 4444557) with the following inventoried Taqman probe assays (ThermoFisher, 4331182); GAPDH: Hs02786624_g1, PGK1: Hs00943178_g1, SNCA: Hs01103383_m1, STMN2: Hs00199796_m1. The amplification was carried out on an Applied Biosciences Vii7 thermal cycler.

#### Stable integration of piggyBac plasmids into iPSCs

Transfection of hiPSCs with the piggyBac constructs was carried out as follows: iPSCs were dissociated into single cells using Accutase (Life Technologies, 00-4555-56) and replated at a density of 1.5×10^6^ cells in one well of a 6-well plate coated with Matrigel (Corning, 354230). The following day, 2 μg of piggyBac construct pEXP-piB-BsD-Tet-NGN2-Puro-SNAP-PGKtk, 1.5 μg transposase pEF1α-hyPBase, and 10.5 μL TransIT-LT1 transfection reagent (Mirus, MIR2300) were added to 200 mL serum-free OPTI-MEM (Gibco, 31985062). The transfection mix was incubated at room temperature for 20 min and added to cell culture containing 2 mL StemFlex medium (Gibco, A3349401) that supports the robust expansion of feeder-free pluripotent stem cells, supplemented with 10 μM ROCK inhibitor (Peprotech, 1293823-50MG). After 6 h incubation at 37°C CO_2_ incubator, the medium was changed to StemFlex plus 10 μM ROCK inhibitor. On the second day of transfection, 5 mg/mL blasticidin (InvivoGen ant-bl-1) was added to 2 mL StemFlex plus 10 3bcM ROCK inhibitor. Media change was performed daily. After five days of blasticidin selection in the presence of ROCK inhibitor, cells were cultured in StemFlex without blasticidin or ROCK inhibitor until the culture became confluent. The stably transfected cell line was then ready for passaging and expansion.

#### Stable integration of piggyBac plasmids into U2OS cells

U2OS cells were dissociated using 0.25% Trypsin-EDTA into single cells and replated at 1.5×10^6^ cells in a 6-well plate. On the following day, 2 μg piggyBac construct, 1.5 μg transpose pEF1α-hyPBase and 10.5 μL TransIT-LT1 transfection reagent (Mirus, MIR2300) were added in 200 μL serum-free OPTI-MEM (Gibco, 31985062). The transfection mix was incubated at room temperature for 20 min and was added to the cell culture containing McCoy’s 5A medium (ATCC, 30–2007) supplemented with 10% FBS (Sigma-Aldrich, 12306C-500ML). After 6 h incubation in a 37°C CO_2_ incubator, the medium was changed to McCoy’s 5A media supplemented with 10% FBS. On the second day of transfection, 10 μg/mL blasticidin (InvivoGen ant-bl-1) was added to 2 μL growth media (McCoy’s 5A media supplemented with 10% FBS) to select for transfected cells. Media was changed every 3 days. After 5 days, the stably transfected cells were passaged and expanded.

#### U2OS cell culture

The U2OS cell line was purchased from ATCC. U2OS cells were maintained in McCoy’s 5A medium (ATCC, 30–2007) supplemented with 10% heat-inactivated Fetal Bovine Serum (FBS, Sigma-Aldrich, 12306C-500ML) and incubated in 5% CO_2_ at 37°C. U2OS cell lines with piggyBac plasmid integration were cultured in the presence of 10 μg/mL blasticidin (InvivoGen ant-bl-1). piggyBac transgene was induced by adding 100 ng/mL doxycycline (Sigma-Aldrich, D9891) to media. Media was changed every other day. Confluent cells (80–100% confluency) were passaged by washing cells with 1X DPBS (Corning, 21-031-CV) and incubating with Trypsin-EDTA (Gibco, 25200114) at 37°C for 3 min. Once cells had lifted, DMEM was added and the cell suspension transferred to a Falcon tube for centrifugation at 500*g* for 5 min. The resulting cell pellet was resuspended in McCoy’s 5A with 10% FBS media, counted, and plated at the desired cell density. Mycoplasma testing was performed every other week on medium from overnight cultures. U2OS cell confluence and inclusion area (GFP) (Figures S9A and S9B) were tracked and quantified with Incucyte live-cell analysis instrument (Sartorius).

#### iPSC generation and lines

Fibroblasts obtained from a female Contursi kindred (*SNCA* A53T) and a female Iowa kindred patient (*SNCA* triplication) were previously described in.^27,28^ Fibroblasts from a male Iowa kindred patient (age 48) with severe early-onset parkinsonism were collected under Stanford protocol (IRB-15028) and previously described in.^28,29^ Fibroblast cultures were subjected to mRNA-based reprogramming at Cellular Reprogramming Inc. using engineered and chimeric transcription factors to facilitate lineage conversion.^108–110^ Polyclonal pools were generated. Resulting iPSC colonies were further expanded on rLaminin-521 (BioLamina, LN521) in Nutristem XF media (Corning, 40-05-100-1A) for at least 3 passages prior to freezing; iPSCs were maintained on Matrigel in StemFlex Medium (Thermo Fisher Scientific, A3349401). iPSC identity was confirmed via staining for pluripotency markers Oct4 and Tra-1-60.

Isogenic *SNCA* knock-down/-out controls were obtained via CRISPR-Cas9 mediated gene editing. Generation of SNCA allelic series in the female Iowa kindred patient line was described in.^27^ For generation of SNCA allelic series in the male Iowa kindred patient line, Guide4 (sequence: 5′ GCCATGGATGTATTCATGAA) targeting exon 2 was used to knock out *SNCA*. The sequence is 16 bp downstream of Guide3, which was used for knock-down/-out of the female Iowa kindred line. The *SNCA* knock-down/-out lines from the male Iowa kindred patient were made using Neon transfection system to transfect ribonucleoprotein (RNP) of sgRNA and Cas9 protein with the standard protocol for iPSC transfection. The sgRNAs were purchased from Synthego and the TrueCut Cas9 protein was purchased from ThermoFisher. Genotypes were subsequently confirmed by Sanger sequencing as described in Hallacli et al.^27^ Quality control steps for selected clones include normal karyotype, confirmation of trilineage pluripotency, and copy number variation at the Bcl2-2 via CGH array. αS protein levels were assessed across the isogenic series (4-copy, 2-copy, 0-copy) via western blot. All *SNCA* triplication experiments in this study were performed with the male Iowa kindred patient iPSC line.

#### Conventional feeder-based hESC culture

WIBR-3 (clone 38)-derived hESCs were routinely cultured as colonies on a monolayer of mouse embryonic fibroblasts (MEFs) and served as starting material for conventional human cortical neuron (c-N) differentiation (Figures S1F, left, and S1G), as well as for immunoblotting (Figure S1D, inset) and Southern blotting (Figure S1E).

hESCs were kept in 6-well plates at 37°C with 5% O_2_ and 3% CO_2_; cell culture and wash media were pre-warmed to 37°C before use. Primary MEFs were prepared and mitotically inactivated using mitomycin C (Sigma-Aldrich) as described previously.^111^ MEFs were plated at a density of 4 × 10^5^ cells/cm^2^ onto gelatinized cell culture plates, which were prepared by incubating with a 0.2% (w/v) gelatin solution (Sigma-Aldrich) for 1 h at 37°C. hESC cultures were supplied daily with hESC medium, and tested for mycoplasma infection every 2–4 weeks according to the manufacturer’s instructions (MycoAlert, Lonza, LT07-318). hESC medium was DMEM/F-12, HEPES (Thermo Fisher Scientific), supplemented with 15% (v/v) Hyclone defined fetal bovine serum (Hyclone Laboratories), 5% (v/v) Knockout serum replacement (Thermo Fisher Scientific), 1% (v/v) GlutaMAX supplement (Thermo Fisher Scientific), 1% (v/v) 100X MEM non-essential amino acids (Thermo Fisher Scientific), 1% (v/v) penicillin-streptomycin (Thermo Fisher Scientific), 0.1 mM β-mercaptoethanol (Sigma-Aldrich) and 4 ng/mL fibroblast growth factor 2 (FGF2; R&D Systems). hESCs cultures were passaged, either manually or enzymatically, when they reached 70–80% confluency. For manual passaging, colonies were cut in a grid excluding differentiated parts, using a stereomicroscope and a 26-G needle that was attached to a 1 mL syringe and bent to a 45° angle. The colony fragments were dislodged and collected in a 15 mL centrifuge tube primed with hESC medium. Colony fragments were re-plated at a 1:3–1:6 ratio onto fresh 6-well feeder plates, which were primed with hESC medium. For enzymatic passaging, differentiated parts were removed by aspiration with a glass Pasteur pipette, followed by incubation with 1.5 mg/mL collagenase type IV (Thermo Fisher Scientific) in DMEM/F-12 (Thermo Fisher Scientific) for 15–30 min at 37°C. Colonies were washed off the plate with DMEM/F-12, collected in a 15 mL centrifuge tube and broken into smaller fragments by trituration. The supernatant was aspirated, the colony fragments were washed with DMEM/F-12 twice and were reconstituted in hESC medium. hESCs were replated as described above.

To achieve clonal purity, hESC colonies were grown from a single cell suspension plated at very low seeding density. The resultant colonies were exposed to 2 μg/mL doxycycline for 10 days (*SNCA-mK2-AAVS1*) or 21 days (*GFP-AAVS1*) from DIV10 for the induction of transgene expression. The micrographs showing transgene-driven GFP/mKate2 fluorescence in Figures S1F–S1H were obtained using an inverted epifluorescent microscope (Eclipse Ti, Nikon Instruments), and were visualized and processed with the NIS-Elements AR software package (Nikon). In preparation for creating a single cell suspension, routinely grown feeder-based hESC cultures were pre-incubated for 30 min at 37°C with hESC medium that was supplemented with 10 μM of the small molecule Y-27632 (Stemgent); Y-27632 was used as rho-associated protein kinase (ROCK) inhibitor (RI). After a wash step with Dulbecco’s phosphate-buffered saline (DPBS; Thermo Fisher Scientific), cells were incubated with StemPro Accutase cell dissociation reagent (“Accutase”; Thermo Fisher Scientific) for 10 min at 37°C. Accutase was diluted with hESC medium/RI, the cell suspension was collected in a 15 mL centrifuge tube and each well was washed with hESC medium/RI. Cells were centrifuged at 350*g* for 10 min, resuspended in hESC medium/RI, followed by trituration to create a single cell suspension and filtered through a 40 μm cell strainer. The single cell suspension was further diluted with hESC medium/RI and re-plated at a density of 50–2,000 cells per well of a 6-well feeder plate. ROCK inhibitor was withdrawn after 48 h.

#### Conventional human cortical neuron (c-N) differentiation

For conventional neuronal differentiation (c-N^SNCA-mK2-AAVS1^ in Figure S1F left, and c-N^GFP-AAVS1^ in Figure S1G), WIBR-3 (38)-derived hESC lines harboring integration of *SNCA-mK2* or *GFP* transgene, respectively, were cultured feeder-free prior to differentiation and neuralized by embryoid body (EB) formation. Neural progenitor cells were differentiated into cortical neurons of anterior forebrain identity.^28^ The full protocol has been published previously^28,76^ and results in cell cultures that are enriched for VGLUT1+ glutamatergic neurons and also contain a fraction of GFAP+ astrocytes.^28^

#### Induced neuron differentiation

On day 0, iPSCs at ~95% confluency were lifted by incubating with Accutase (Life Technologies, 00-4555-56), a natural enzyme mixture with proteolytic and collagenolytic enzyme activity, for 4 min at room temperature, combined with equal volume of StemFlex media (Gibco, A3349401), centrifuged at 800 rpm for 4 min, resuspended in StemFlex media, and counted. Cells were seeded at a density of 1.25 × 10^6^ cells per well (for 6-well plates) with 0.5 μg/mL doxycycline (Sigma, D9891-5G) to induce expression of Ngn2 on the piggyBac transgene. For 10-cm plates, 10 million cells were seeded. This was considered day 0 (“days *in vitro*” DIV0). Plates were previously coated with Matrigel (Corning, 354230). For the first 2 days of neuron differentiation, media change was conducted daily with Neurobasal N2/B27 media (1X B27 supplement (Life Technologies, 175-4-044), 1X N2 supplement (Life Technologies, 17502-048), 1X Non-Essential Amino Acids (Gibco, 11140–050), 1X GlutaMAX (Gibco, 35050–061), 1X Pen-Strep (Life Technologies, 15070–063), Neurobasal Media (Life Technologies, 21103–049)), 5 μg/mL blasticidin (Invivogen, #ANT-BL-1) and 0.5 μg/mL doxycycline (Sigma, D9891-5G); for days 3–6, media changes were done the same as for days 1–2, with the addition of 1 μg/mL puromycin (Fisher Scientific, #ANT-PR-1) to select cells expressing the piggyBac transgene.

On day 7 (DIV7), Accutase was used to dissociate the neurons before re-plating them onto the appropriate polyethyleneimine (PEI) (Sigma, 181978-100G)/laminin (Sigma, L2020)-coated plates for downstream assays (e.g., 3 million cells per well of 6-well, 1 million cells per well of 24-well, 50,000 cells/well of 96-well plates). The following day (day 8, DIV8), an equal volume of Neurobasal N2/B27 media supplemented with 20 ng/mL Brain-derived Neurotrophic Factor (BDNF; Peprotech, 450–02), 20 ng/mL Glia-derived Neurotrophic Factors (GDNF; Peprotech, 450–10), 2 mM Dibutyryl cyclic AMP (cAMP; Sigma, D0260), 2 μg/mL laminin (Sigma, L2020), 0.5 μM cytosine β-D-arabinofuranoside hydrochloride (AraC; Sigma) was added to the existing cell media. Doxycycline was withdrawn from medium on day 8. For neurons transfected with the all-in-one piggyBac transgene containing *NGN2* and *SNCA*, doxycycline supplementation was continued throughout neuronal culture to maintain αS overexpression. At day 11 (DIV11), media change occurred with equal volumes of Neurobasal N2/B27 and Neurobasal Plus (Life Technology, A35829-01) N2/B27 Plus media, and 10 ng/mL BDNF, 10 ng/mL GDNF, 1 mM cAMP, 1 μg/mL laminin. At day 14 (DIV14), half media change occurred with Neurobasal Plus media, 10 ng/mL BDNF, 10 ng/mL GDNF, 1 mM cAMP, 1 μg/mL laminin. Thereafter, half media change occurred every three days with Neurobasal Plus media, 10 ng/mL BDNF, 10 ng/mL GDNF, 1 mM cAMP, 1 μg/mL laminin.

For shRNA knockdown experiments, the following were used: shRNA-Control is MISSION pLKO.1-puro non-mammalian shRNA control plasmid DNA (Sigma, SHC002), which targets no known mammalian genes, and shRNA-RHOA is MISSION pLKO.1-shRNA against *RHOA* (Sigma, TRC clone ID TRCN0000047710). Glycerol stocks of predesigned shRNA clones were purchased from Sigma. The pCDH-CAG-RFP plasmid (generously provided by Marty Fernandez and Tracy Young-Pearse, BWH) consists of mRFP cloned into the EcoRI site of pCDH-CB (Addgene plasmid 72267) through In-Fusion cloning (Takara). Lentivirus packaging of shRNA and RFP constructs was done by Boston Children’s Hospital Viral Core Facility. For knockdown in neurons, a full-media change with day 11 media plus shRNA lentivirus at MOI 5, 10, 20, or 30 was conducted on day 10 (DIV10). A full-media change was conducted on day 11 (DIV11) to remove the virus. For Biostation imaging experiments, shRNA lentivirus (at MOI 5, 10, 20, or 30) was co-transduced with pCDH-CAG-RFP at MOI30 for sparse labeling of neurons (see more details in section “culturing of induced inclusion neurons for live imaging with BioStation CT” within “automated longitudinal single-cell survival tracking” methods).

#### Induced astrocyte differentiation

On day 0, H1 hESCs at ~95% confluency were dissociated with Accutase, and 4 × 10^6^ cells were replated in Matrigel-coated 10-cm dishes using StemFlex medium with 10 mM ROCK inhibitor (StemCell Technologies, Y-27632) and 500 ng/mL doxycycline to induce human NFIB or NFIB-SOX9 expression. On days 1 and 2, cells were cultured in Expansion medium (DMEM/F-12, 10% FBS, 1% N2 supplement, 1% Glutamax (Thermo Fisher Scientific). From days 3 to day 5, Expansion medium was gradually switched to FGF medium (Neurobasal, 2% B27 supplement, 1% NEAA, 1% Glutamax, and 1% FBS (Thermo Fisher Scientific); 8 ng/mL FGF, 5 ng/mL CNTF, and 10 ng/mL BMP4 (Peprotech). On day 6, the mixed medium was replaced by FGF medium. Selection was carried out on days 1–6 with 5 μg/mL blasticidin for cell lines harboring vectors conferring blasticidin resistance. On day 7, cells were dissociated with Accutase and replated in Matrigel-coated wells. The day after, FGF medium was replaced, and afterward 50% of the medium was replaced by Maturation medium (1:1 DMEM/F-12 and Neurobasal, 1% N2, 1% sodium pyruvate, and 1% Glutamax (Thermo Fisher Scientific); 5 μg/mL *N*-acetyl-cysteine, 500 μg/mL dbcAMP (Sigma-Aldrich); 5 ng/mL heparin-binding EGF-like growth factor, 10 ng/mL CNTF), 10 ng/mL BMP4 (Peprotech) every 2–3 days, and cells were kept for 21 days.

#### Recombinant αS expression and purification

Lyophilized monomeric αS was provided by Dr. Tim Bartels. Briefly, plasmid pET21a-SNCA was expressed in BL21(DE3) *E. coli*. After cell lysis, αS was purified via ion-exchange chromatography (5 mL HiTrap Q HP columns, GE Life Sciences, 17516301) and size exclusion chromatography (13 mL HiPrep 26/60 Sephacryl S-200 HR, GE Life Sciences, 17119501) using the ÄKTAprime plus FPLC system^112^ and subsequently lyophilized in protein low binding tubes (Eppendorf).

#### Generation of preformed αS fibrils

For the generation of wild-type recombinant preformed fibrils (PFFs), 1 mg of lyophilized monomeric wild-type αS was reconstituted with 100 μL of sterile DPBS (pre-cooled to 4°C) on ice without further resuspending. Tubes were then rotated on a tube rotator for 10 min at 4°C and subsequently centrifuged for 10 min at 15,000 *g* at 4°C to pellet preformed aggregates. The supernatant was then transferred to a new protein low binding tube and concentration was measured spectrophotometrically using Nanodrop (A280 – MW = 14,5 kDa; Extinction coefficient ε for human αS = 5,960 M^−1^cm^−1^). Samples were then diluted down to a final concentration of 5 mg/mL and aliquoted into 100 μL aliquots. A 1–2 mL aliquot was diluted down to 500 μg/mL for electron microscopy and flash-frozen in a dry ice/ethanol slurry. Samples were placed into an orbital thermomixer with a heated lid for 7 days at 37°C, shaking at 1,000 rpm. At the end of the 7-day period, the contents of the tube appeared turbid. The tube was gently flicked to resuspend preformed fibrils, aliquoted in 1–2 μL volumes for TEM, and flash-frozen in a dry ice/ethanol slurry prior to storage at −80°C.

A53T PFFs generated from αS-A53T monomer was kindly provided by Dr. Kelvin Luk.

#### PFF seeding in iPSC-derived cortical neurons

For PFF seeding of cortical neurons, WT PFFs were used for neurons overexpressing WT αS, and A53T PFFs for αS-A53T overexpressing lines to match the amino acid sequence of intracellular overexpressed αS and PFF strain. On DIV11, a PFF aliquot and PBS aliquot (negative control) were thawed at room temperature for 2–3 min and subsequently transferred to an ice bucket for water bath-based sonication using the Bioruptor Plus (Settings: High power, 10 cycles, 30 s on – 30 s off per cycle, temperature 10°C). Sonicated samples were subsequently transferred into the tissue culture hood and diluted in culture medium to a concentration of 10 μg/mL. At DIV14, no media was removed, but instead the same amount of fresh medium was added. From DIV18, half media changes were performed according to NGN2 protocol.

#### PFF seeding in U2OS cells

For seeding of U2OS cells, A53T PFFs were used for cells overexpressing αS-A53T-sfGFP or αS-A53T-ΔNAC-sfGFP to match the amino acid sequence. On Day −2, Cells were plated at 2000 cells/well in 96-well plate (Greiner) in 100 μL McCoy’s 5A medium (ATCC, 30–2007) supplemented with 10% FBS (Sigma-Aldrich, 12306C-500ML), 10 μg/mL blasticidin (InvivoGen, ant-bl-1) and 100 ng/mL doxycycline (Sigma-Aldrich, D9891). Two days later (48 h post-induction of transgene), cells were seeded with PFFs by quick-thawing an aliquot of PFFs in water-bath and re-sonicating with Bioruptor Pico (10°C, 10 cycles, 30 s ON, 30 s OFF, HIGH power). PFFs were resuspended in media at desired concentration (0, 1, 3, 5 and 10 μg/mL) and added to cells. This was considered Day 0. On Day 3 (72 h post-seeding), an equal volume of fresh media (without PFFs) was added to the wells. On Day 6, cells were fixed in 4% paraformaldehyde (PFA), 20% sucrose in PBS for immunostaining.

#### Electron microscopy

For electron microscopy of PFFs, 5 μL of 500 μg/mL PFFs was adsorbed for 1 min to a carbon coated grid (Electron Microscopy Sciences, CF400-CU) that had been made hydrophilic by a 20 s exposure to a glow discharge (25 mA). Excess liquid was removed with a filter paper (Whatman #1), the grid was then floated briefly on a drop of water (to wash away phosphate or salt), blotted again on a filter paper and then stained with 0.75% uranyl formate (Electron Microscopy Sciences, 22451) or 1% uranyl acetate (Electron Microscopy Sciences, 22400) for 20–30 s. After removing the excess stain with a filter paper the grids were examined in a JEOL 1200EX Transmission electron microscope or a TecnaiG^2^ Spirit BioTWIN and images were recorded with an AMT 2k CCD camera.

For electron microscopy of induced neurons, iPSC-derived neurons were seeded on day 7 (DIV7) at 0.6 million cells/well on ACLAR plastic discs pre-coated with polyethyleneimine (PEI) (Sigma, 181978-100G) and laminin (Sigma, L2020) in 12-well plate (Corning). At 25 days of differentiation (DIV25), iPSC-derived neurons were fixed in 2.5% glutaraldehyde (Electron Microscopy Sciences, 16220), 1.25% paraformaldehyde (Electron Microscopy Sciences, 15710), 0.03% picric acid (Electron Microscopy Sciences, 19550) in 0.1 M sodium cacodylate buffer (pH 7.4) (Electron Microscopy Sciences, 12300) for 60 min. After 3 washes in 0.1M cacodylate buffer, post-fixation was done in 1% osmium tetroxide (OsO4) (Electron Microscopy Sciences 19150)/1.5% potassiumferrocyanide (KFeCN6) (Sigma, P9387) for 30 min followed by 3 washes in dH_2_O. Coverslips were then incubated in 15 aqueous uranyl acetate (Electron Microscopy Sciences, 22400) for 30 min, washed twice in dH_2_O and subsequently dehydrated in grades of alcohol (5 min each; 50%, 70%, 95%, 2 × 100%). Cells were then embedded in TAAB Epon (TAAB, UK, T022) and polymerized at 60°C for 48 h. Ultra-thin sections (about 80 nm) were cut on a Reichert Ultracut-S microtome and picked up onto copper grids. For immunogold labeling, the sections were etched using a saturated solution of sodium metaperiodate (Sigma 1878) in water for 5 min at room temperature. Grids were then washed 3 times in dH_2_O and floated on 0.1% Triton X-100 (TX-100) (Sigma, T8787) for 5 min at room temperature. Blocking was carried out using 1% BSA +0.1% TX-100/PBS for 1 h at room temperature. Grids were incubated with an anti-GFP antibody (1:50; Abcam, 6556) in 1% BSA +0.1% TX-100/PBS overnight at 4°C. Grids were washed three times in PBS to remove unbound antibody followed by incubation with 10 nm or 15 nm protein-A gold (University Medical Center, Utrecht, the Netherlands) for 1 h at room temperature. Grids were washed with PBS and water, stained with lead citrate (Electron Microscopy Sciences, 17800) and examined in a JEOL 1200EX Transmission electron microscope (JEOL USA Inc. Peabody, MA USA) and images were recorded with an AMT 2k CCD camera (Advanced Microscopy Techniques, Woburn, MA).

#### Whole-cell protein extraction

For cell lysis, frozen cell pellets were thawed on wet ice and resuspended in 100 μL of 1X NuPage LDS sample buffer (Life Technologies, NP0007) (diluted using dH_2_O) containing protease (cOmplete EDTA-free Protease Inhibitor Cocktail, Sigma Aldrich, 11873580001) and phosphatase inhibitors (PhosSTOP phosphatase inhibitor cocktail, Sigma Aldrich, 4906845001). Samples were sonicated twice for 15 s with a tip-sonicator at 40% power, keeping sample on ice and centrifuged at maximal speed for 10 min. The supernatant was then transferred to a new Eppendorf tube and the cell pellet discarded. To quantify protein concentration, 5 μL of each sample was used in Pierce BCA Protein Assay Kit (Thermo Fisher, 23227) according to manufacturer’s guidelines.

#### Western blotting

For western blots, 30 μg of protein per sample was subjected to SDS-PAGE using NuPAGE 4–12% Bis-Tris protein gels (Life Technologies, NP0322BOX) in NuPAGE MES SDS Running Buffer (Life Technologies, NP000202), electrophoresed at 150 V for 55 min or until the protein ladder and the loading dye indicated a sufficient electrophoretic separation. Dry transfer from polyacrylamide gel to nitrocellulose membrane was conducted with the iBlot 2 Gel Transfer Device (Thermo Fisher) using preset P0 program (20V 1 min; 23V 4 min; 25V 2 min). The membrane was fixed in 4% paraformaldehyde in dH2O (to improve αS detection^113^ for 30 min at room temperature with orbital shaking and washed three times for 5 min with PBS. Membranes were blocked in Li-COR Odyssey blocking buffer (PBS) for 1 h with orbital shaking and subsequently incubated overnight in primary antibody solution, i.e., Odyssey blocking buffer (PBS) (Fisher Scientific, NC9877369), 0.1% Tween 20 (Fisher Scientific, 50213612) and the respective primary antibodies at the desired dilution, at 4°C with orbital shaking. After four washes for 5 min in 0.05% Tween 20/PBS, membranes were incubated with the secondary antibody solution, i.e., Odyssey blocking buffer (TBS), 0.1% Tween 20 and secondary antibody (IRDye 800CW (LI-COR Biosciences, 925–32214), IRDye 680RD (LI-COR Biosciences 925–68074)) at 1:10,000 dilution, at room temperature with orbital shaking and protected from light. After four washes for 5 min in 0.05% Tween 20/PBS, the blot was scanned using the Odyssey CLx Infrared Imager. For quantification, ImageStudio software was used.

For the doxycycline dose-response shown in Figure S1D (inset), routinely grown WIBR-3 (38)-derived hESC cultures harboring integration of wild-type *SNCA* transgene were exposed to different concentrations of doxycycline for 30 h. Control hESC samples were cultured in the absence of doxycycline. After the 30 h treatment, hESCs were harvested and lysed for immunoblotting. Media residues were washed with 4°C chilled DPBS. Fresh chilled DPBS was added and the cell monolayer was detached with a cell scraper or a micropipette. Samples were kept on ice from this point on until cell lysis. Samples were centrifuged at 400*g* for 5 min in a 4°C refrigerated bench-top microcentrifuge. The supernatant was aspirated and fresh chilled DPBS was added, followed by centrifugation as above. The supernatant was aspirated and the cell pellets were resuspended in cell lysis buffer (2 mM EDTA, 1% [v/v] 100X protease inhibitor cocktail, 2% [w/v] SDS, 50 mM Tris-HCl in deionized water [diH_2_O]; all Sigma-Aldrich). The cell lysates were boiled for 10 min at 100°C, followed by centrifugation at 10,000*g* for 10 min. The supernatant was transferred to a new Eppendorf tube and the total protein concentration of cell lysates was determined using the Pierce bicinchoninic acid (BCA) protein assay kit (Thermo Fisher Scientific, 23227) following the manufacturer’s instructions. Briefly, 0.5–1 μL cell lysate were diluted with diH_2_O to a total volume of 25 μL (1:25–1:50 dilution). 50 parts BCA reagent A were mixed with one part BCA reagent B (50:1 ratio) and 200 μL of this solution was mixed with the diluted cell lysate, 25 μL diH_2_O (as a blank control) or 25 μL of a bovine serum albumin (BSA) standard (20–500 μg/mL BSA in diH_2_O), each in triplicate. After incubation for 26 min at 37°C the absorbance of the purple-colored reaction products was measured at 562 nm using a spectrophotometer (Epoch 2 microplate reader, BioTek). The results were averaged and corrected by the blank control. The protein concentration was determined using a standard curve derived from the BSA standards. Next, the extracted proteins were separated using a one-dimensional SDS-PAGE. According to the manufacturer’s instructions (NuPAGE SDS-PAGE gel electrophoresis system, Thermo Fisher Scientific), 20 μg bulk protein were mixed with NuPAGE LDS sample buffer and NuPAGE sample reducing agent and heated to 75°C for 10 min. The protein samples were loaded onto a NuPAGE 10% Bis-Tris gel, together with a protein molecular weight standard (Precision Plus Protein Dual Color Standards, Bio-Rad, 1610374). NuPAGE MES SDS was used as electrophoresis buffer and electrophoresis was conducted at 150 V until the loading dye and the molecular weight control indicated sufficient separation (40–120 min). Electrophoretic transfer to a polyvinylidene difluoride (PVDF) membrane was carried out by wet electrophoretic transfer using an electrophoresis cell (Criterion Cell, Bio-Rad). The NuPAGE gel was briefly washed in transfer buffer (Abbiotec) that was prepared according to the manufacturer’s instructions, and assembled with an activated PVDF membrane (10-s activation in methanol) and Whatman filter papers. The transfer was carried out with chilled transfer buffer at 4 °C at either 60 V for 120 min for one transfer or 65 V for 150 min for two simultaneous transfers. In preparation for chemiluminescent detection, PVDF membranes were briefly washed in PBS (Boston BioProducts) and from this point on all incubation and wash steps were performed on an orbital shaker. Immunoblots were immersed in a blocking solution, consisting of 5% (w/v) nonfat milk powder (Bio-Rad) in PBS with 0.1% (v/v) Tween 20 (Sigma-Aldrich), for 40 min, followed by incubation with mouse anti-α-synuclein (BD Biosciences; 1:500) and mouse anti-GAPDH (EMD Millipore; 1:3,000) primary antibodies, diluted in blocking solution, overnight at 4°C. The next day, immunoblots were briefly washed three times with diH_2_O and three times for 10 min with PBST (PBS with 0.1% [v/v] Tween 20), and then incubated for 1 h in blocking solution, supplemented with horseradish peroxidase (HRP)-conjugated rabbit anti-mouse IgG secondary antibody (Sigma-Aldrich; 1:10,000). Immunoblots were briefly washed three times with diH_2_O and three times for 10 min with PBST. Immunoblots were exposed to SuperSignal West Pico Chemiluminescent Substrate solution (TFS) according to the manufacturer’s instructions and exposed to a film that was developed using a Kodak X-OMAT 1000A film processor.

#### Immunofluorescence and microscopy

Immunofluorescence analysis was performed as follows. iPSC-derived neuron cultures grown in 96-well glass bottom plates (Brooks Life Science Systems, MGB096-1-2-LG-L) were fixed with 100 μL of 4% paraformaldehyde in PBS for 15 min. Cells were blocked and permeabilized using 10% goat serum, 0.1% Saponin (to preserve integrity of lipid-rich inclusions) (Boston Bioproducts, BM-688) in PBS for 1 h at room temperature. Primary antibody was incubated in 2% goat serum, 0.02% Saponin overnight at 4°C. Cells were washed three times with PBS, 5 min per wash, and incubated with secondary antibody in 2% goat serum, 0.02% Saponin and 0.05% Hoechst for 1 h at 37°C. Finally, cells were washed three times with PBS, 5 min per wash. Images of the immunostained cells were captured with a Nikon TiE/C2 confocal microscope.

#### Immunostaining of c-N^GFP-AAVS1^

Conventionally differentiated c-N^GFP-AAVS1^ neuronal cell cultures shown in Figure S1G were plated at high density (500,000 to 1×10^6^ cells/cm^2^) onto poly-D-lysine (2 mg/mL, Sigma) and mouse laminin (1 mg/mL, BD Biosciences)-coated 8-well chambered cover glasses (Lab-Tek, Thermo Fisher Scientific) as described previously.^28^ From DIV8, c-N^GFP-AAVS1^ were exposed to 2 μg/mL doxycycline for 3 weeks. On DIV29, cell cultures were washed in DPBS for 5 min, followed by fixation in 4% (w/v) paraformaldehyde (Electron Microscopy Sciences) in DPBS for 15 min, and washed in DPBS another three times for 5 min. Samples were permeabilized and blocked in a blocking solution, consisting of 10% (v/v) normal donkey serum (Jackson ImmunoResearch) in DPBS with 0.1% (v/v) Triton X-100 (Sigma-Aldrich), for 1 h. Conventionally differentiated c-N cell cultures typically contain a fraction of astrocytes,^28^ which were visualized with immunostaining for the astroglial marker GFAP together with immunostaining for GFP, in order to visualize even low levels of transgene expression. Samples were incubated with rabbit anti-GFAP (Dako, Agilent Technologies) and mouse anti-GFP (Roche Diagnostics) primary antibodies, each diluted 1:1,000 in blocking solution, overnight at 4°C. The next day, samples were washed three times for 5 min with PBST (DPBS with 0.1% [v/v] Triton X-100), and then incubated for 1 h in blocking solution, supplemented with fluorochrome-conjugated donkey anti-rabbit and donkey anti-mouse secondary antibodies (Thermo Fisher Scientific), diluted 1:500, as well as 10 μg/mL Hoechst 33342 nuclear counterstain (Thermo Fisher Scientific). Samples were washed twice for 5 min with PBST and 8-well chambers were supplied with DPBS. Samples were imaged with a multispectral spinning disk confocal microscope (Ultraview PerkinElmer; Zeiss Axiovert 200 inverted microscope; 100X Zeiss 1.4 NA oil immersion lens); images were visualized and processed with Volocity software package (PerkinElmer).

#### Lattice light-sheet microscopy and 3D rendering

Lattice light-sheet microscopy was performed using the lattice light-sheet mode of a custom built Multimodal Optical Scope with Adaptive Imaging Correction (MOSAIC). Neurons were plated on a 25 mm coverslip and imaged at diffraction limited resolution using a Special Optics 0.65 NA, 3.74 mm working distance water dipping objective for excitation and a Zeiss 1.0 NA, with 2.2 mm working distance water-dipping objective for detection on two Hamamatsu Orca Flash 4.0 v3 sCMOS cameras. Upon image deconvolution of the raw data files, 3D surface rendering was performed using Imaris.

#### Sequential extraction of insoluble fraction from human brain

Sequential extraction from human brain was performed according to Peng et al.^3^ Briefly, 0.5 mg of brain tissue was homogenized in high salt (HI) buffer (50 mM Tris-HCl, 750 mM NaCl, 5 mM EDTA, 10 mM NaF, pH 7.40) containing protease inhibitors. After ultracentrifugation at 100,000*g* for 30 min at 4°C, supernatant was removed, and fresh HI buffer added. The same steps were subsequently repeated with HI buffer containing 1% Triton, HI buffer with 1% Triton and 30% sucrose, HI buffer with 1% sarkosyl, and finally in PBS to resuspend the sarkosyl-insoluble fraction of the brain homogenate enriched in aggregated αS.

#### Seed amplification assay (SAA)

Flash-frozen MSA and PD brain tissue (500 μg, frontal cortex) was homogenized and subjected to serial extraction using detergents in increasing strength and subsequent ultracentrifugation to obtain an insoluble protein fraction containing aggregated αS as previously described.^3^ For SAA^40^ to amplify and monitor αS aggregates, 10 μL of brain-derived seed was incubated with recombinant monomeric αS at 42°C in a BMG FLUOstar Omega plate reader to amplify amyloid αS by incorporating monomeric αS into the growing aggregate. Before each SAA experiment, lyophilized monomeric protein was dissolved in 40 mM phosphate buffer (pH = 8), filtered using a 0.22 mm filter, and the concentration of recombinant protein was measured via absorbance at 280 nm using a Nanodrop One spectrophotometer. Brain-derived insoluble protein was tip-sonicated for 30 s (1 s off, 1 s on) at 30% of amplitude and added to a 96 well plate with 230 mM NaCl, 0.4 mg/mL αS and a 3 mm glass bead (Millipore Sigma 1040150500). Repeated shaking (1 min incubation, 1 min double-orbital shaking at 400 rpm) disrupts the aggregates to produce an expanded population of converting units. The amyloid dye thioflavin T was used in adjacent wells to monitor the increase in fibrillar content via fluorescence readings at 480 nm every 30 min until the signal plateaued toward the end of the amplification interval of 6 days.

For SAA on neuronal lysates, neurons were harvested at DIV28 and pelleted at 376 rcf for 5 min. Pellets were sonicated in 100μL PBS, supplemented with 1% Triton X-100 and proteinase inhibitors (Roche), centrifuged for 5 min at 2,000 rcf, and supernatant was aliquoted. A 2 μL of 1:10^6^ diluted (in PBS) lysate was added to 98 μL SAA buffer (PIPES pH 6.5, 500 mM NaCl, 10 μM ThT, 0.5 mg/ml recombinant αS, 5–8 beads of OPS 0.8 mm silica glass) and incubated for 40 h at 37°C, with double-orbital shaking for 1 min at 400 rpm every 5 min. Fluorescence reading was taken every 45 min (Excitation: 448, Emission: 482, Gain: 1200, Settling time: 0.5s). Each lysate was tested in technical triplicate.

#### ELISA

To determine the concentration of αS after amplification using SAA, an αS ELISA protocol from MSD was used. For sulfo-tag labeling of detection antibodies, 200 μL of SOY1 antibody (1.37 mg/mL in PBS) was incubated at room temperature for 2 h with 16 μL of 3 nmol/μL MSD NHS-Sulfotag reagent (150 nmol freshly suspended in 50 μL PBS). Next, 250 μL PBS was added to antibody solutions, concentrated using Amicon ultra filter tubes (10,000 MWCO), and brought up to 500 μL PBS again. This was repeated 5 times to dilute out the tag reagent. Protein concentration was subsequently measured using BCA assay. For plate preparation, MSD Standard plates were coated with 30 μL of 200 ng filtered 2F12 (1 mg/mL) from recently filtered batches diluted in PBS and stored overnight at 4°C. Plates were then tapped out, blocked with 150 μL per well in 5% MSD blocker A in 0.05% PBS-T, sealed and placed on an orbital shaker for 1 h at room temperature. Plates were subsequently washed 5 times with 150 μL PBS-T per well, samples were added in OG-RIPA, PBS-T with 1% MSD blocker A, as well as recombinant αS at different concentration gradients in PBS-T with 1% MSD blocker A (0.5% NP-40) and incubated for 2 h at room temperature with orbital shaking. Plates were washed 5 times with 150 μL TBS-T per well prior to addition of detection antibody solution, i.e., 30 μL per well of 200 ng sulfo-tagged SOY1 antibody in PBS-T with 1% MSD blocker A. Plates were incubated for 1 h at room temperature with orbital shaking and protected from light. After 5 washes with PBS-T, 150 μL of 2X MSD reader buffer diluted in MilliQ water was added and the plate was read with the Meso Sector S 600.

#### Proteinase K digest

Sarkosyl-insoluble and SAA-amplified samples were treated with 1 μg/mL of proteinase K at 37°C for 1 h in gentle shaking. The digestion was stopped by adding NuPAGE LDS sample buffer and boiling the sample at 95°C for 7 min. Samples were then loaded onto a Novex 16% Tricine gels (Invitrogen) for protein separation. After electrophoresis, gels were incubated in 20% ethanol for 5 min at room temperature and blotted onto iBlot 2 NC Regular Stacks (Invitrogen) using the iBlot Dry Blotting. The membrane was rinsed in ultrapure water and incubated in 4% paraformaldehyde/PBS for 30 min at room temperature. The membranes were blocked in Odyssey blocking buffer (PBS)/PBS buffer 1:1 (LI-COR) or casein buffer 0.5% (BioRad) for 1 h at room temperature. After blocking, membranes were incubated overnight at 4°C with anti-αS clone 42 (BD Biosciences). After three washes in PBS-Tween-20 0.1%, the membrane was incubated for 1 h at room temperature with the secondary antibody (goat anti-mouse IgG F(Ab)2 conjugated with horseradish peroxidase (HRP), Abcam) in blocking solution. Membranes were washed in PBS-Tween-20 0.1% and then the signal was detected using Invitrogen iBright imaging system and the Luminata Crescendo Western HRP substrate (Millipore).

#### αS Triton X-100/SDS sequential extraction

Sequential extraction of αS with Triton X-100 and SDS was performed as described in.^34^ Briefly, neurons that were seeded at 3×10^6^ cells/well in 6-well plate were rinsed twice with PBS, kept on ice, and scraped in the presence of 250 μL of 1% (v/v) Triton X-100/TBS with protease and phosphatase inhibitors. The lysate was transferred to polyallomar ultracentrifuge tubes and sonicated ten times at 0.5 s pulse and 10% power (Misonix Sonicator S-4000). Samples were incubated on ice for 30 min, then centrifuged at 100,000 *g* at 4°C for 30 min in an ultracentrifuge. The supernatant (Triton X-100 extract) was transferred to a microcentrifuge tube and combined with 4x Laemmli buffer for SDS-PAGE (small aliquot of ~20 mL is saved prior to mixing with Laemmli buffer for protein assay). In the meantime, 250 μL of 1% Triton X-100/TBS was added to the pellet and sonicated ten times at 0.5 s pulse and 10% power, followed by ultracentrifugation at 100,000 *g* at 4°C for 30 min. Next, 125 μL of 2% (w/v) SDS/TBS with protease and phosphatase inhibitors was added to the pellet. The sample was sonicated fifteen times at 0.5 s pulse and 10% power, ensuring that the pellet is completely dispersed. The supernatant (SDS extract) was transferred to a new microcentrifuge tube and diluted to 2x volume for the corresponding Triton X-100 fraction to make the insoluble αS species more abundant and easier to visualize by western blot. For example, 60 μL of 4x Laemmli buffer was added to 180 μL of Triton X-100 extract, and 30 μL of 4x Laemmli buffer to 90 μL SDS extract.

BCA protein assay was performed on the Triton X-100 supernatant and SDS extract. For SDS-PAGE, 5 mg of protein samples were boiled for 5 min, centrifuged for 2 min at maximum speed, and loaded onto 4–12% Bis-Tris gel. The samples were electrophoresed at 150V for approximately 90 min. Protein was transferred to PVDF membrane using iBlot 2 Dry Blotting System (Invitrogen). The membrane was fixed for 30 min in 0.4% PFA/PBS if detecting untagged αS. The membrane was subsequently blocked for 1 h with 5% (w/v) milk/TBS-T before incubating with primary antibody overnight at 4°C with shaking. The primary antibody was diluted in 5% (w/v) milk/TBS-T. The following primary antibodies were used: rabbit anti-pS129 (Abcam, 51253) 1:5000, mouse anti-αS 4B12 (Thermo Fisher, MA1-90346) 1:1000, goat anti-GFP (Rockland, 600-101-215) 1:5000, mouse anti-GAPDH (Thermo Fisher, MA5-15738) 1:5000. After incubation with primary antibody, the membrane was rinsed three times with TBS/T, 10 min with rocking for each rinse. The membrane was then incubated with HRP-conjugated secondary antibodies for 1 h at room temperature, with rocking. The following secondary antibodies were used: anti-rat-HRP (Sigma Aldrich, NA935) 1:10,000, anti-rabbit-HRP 1:10,000 (Bio-Rad, 170–6515), anti-goat-HRP 1:10,000 (R&D Systems, HAF109). The membrane was rinsed three times with TBST/T, 10 min per rinse, with rocking, before developing with chemiluminescence.

#### Seahorse XF cell mito stress test

iPSC-derived neurons were seeded at day 8 (DIV8) on polyethyleneimine (PEI)/laminin coated Seahorse assay plates (Agilent Technologies Inc.) at a density of 1×10^5^ cells/well and cultured for 12 days. For transgenic lines, half of the wells were seeded with 10 μg/mL bath-sonicated recombinant A53T PFFs on day 11 (DIV11). On the day of the experiment, cells were pre-incubated for 1 h in Assay Media (Seahorse XF Base Medium without Phenol Red; Agilent Technologies Inc., 103335–100) supplemented with 10 mM Glucose, 1 mM Pyruvate solution, 2 mM glutamine solution (Agilent Technologies Inc., 103577–100, 103578–100, 103579–100). Measurement of intact cellular respiration was performed using the Seahorse XF96 analyzer (Agilent) and the XF Cell Mito Stress Test Kit (Agilent Technologies Inc., 1103010–100) according to the manufacturer’s instructions. Respiration was measured under basal conditions, and in response to ATP synthase inhibitor oligomycin (2 mM) followed by the addition of the ionophore 4-(trifluoromethoxy) phenylhydrazone (FCCP; 0.75 mM) to induce maximal mitochondrial respiration. Finally, respiration was stopped by adding the mitochondrial complex I inhibitors Rotenone and Antimycin A (0.5 mM).

#### Autophagic flux assay

iPSC-derived neurons were seeded at day 7 (DIV7) on polyethyleneimine (PEI)/laminin coated 24-well plates at a density of 1×10^6^ cells/well and cultured up to DIV56. For the autophagic flux assay, 100 nM bafilomycin (Sigma, B1793) and DMSO for control conditions was added and incubated at 37°C for 12 h prior to cell collection. Whole cell extraction, BCA analysis and western blotting were performed as described earlier (see ‘whole-cell protein extraction’, ‘western blotting’), however excluding membrane fixation in 4% PFA and subsequent wash steps. Primary antibodies used included rabbit anti-actin 1:1000 (Sigma-Aldrich Inc, A2066), mouse anti-p62 1:1000 (Millipore, MABN130), rabbit anti-LC3 1:1000 (Cell Signaling Technology, 2775S). To determine basal LC3-II and p62 levels, bafilomycin-untreated lanes were used and normalized to actin. To assess autophagic flux, the ratio of normalized LC3-II and p62 levels between treated versus untreated samples was calculated.

#### Automated longitudinal single-cell survival tracking

##### Culturing of induced inclusion neurons for live imaging with BioStation CT

Neurons were differentiated and cultured as described in “induced neuron differentiation”. Neurons were seeded on 96-well plate at DIV7 at 50,000 cells/well. On DIV10, neurons were transduced with pCDH-CAG-RFP lentivirus at MOI30 for sparse labeling of neurons, and on DIV11 virus was removed through a full-media change. On DIV11 piN^A53T-sfGFP-pB^ and piN^ΔNAC-sfGFP-pB^ neurons were treated with 10 μg/mL recombinant PFFs. Treatment with virus on DIV10 and with PFFs on DIV11 were both done through a full-media change with day 11 media (see “induced neuron differentiation” section). On DIV13 live imaging in the BioStation CT (Nikon) was initiated with the following settings: 10X objective, 5 × 5 tiled images per well; 2.5 × 100 ms exposure time and 10 nm luminance for excitation at 475 for Ch2; 3 × 100 ms exposure time and 50 nm luminance for excitation at 542 for Ch 3; imaging every 6 h for 10 days.

##### Single-cell inclusion survival tracking and image analysis

Image analysis for algorithm development was performed at Nikon Corporation. Survival curves were plotted with the “survival” package library in R. To detect the morphologic properties of the inclusion neuron models in culture, morphologic masks were designed to identify and quantify the different features, including cell body size, inclusions, neurites, and intensity. After the masks for detection of each cell were optimized, the tracking was performed using the mask for cell body. The setting parameters used for this tracking are described below.

###### Cell and inclusion detection in seeded inclusion model (“PFF2chTracking”).

Detection and tracking of inclusion-positive neurons was based on GFP fluorescence. Only inclusions in the soma were tracked, since it was not always possible to assign individual neuritic inclusions to the originating cell body longitudinally over time. Whereas bright, discrete GFP signal was detected in soma in inclusion-positive neurons, the GFP signal in inclusion-negative neurons was too diffuse for accurate longitudinal single-cell tracking, so inclusion-negative neurons were tracked by RFP fluorescence. For inclusion-positive cells, objects were identified as cell bodies if GFP fluorescence intensity was greater than 60 and length and width were 6–30 pixels (4.8–24 μm). Areas with fluorescent intensities that were the mean fluorescent intensity of the cell body plus 70 or higher were identified as inclusions. Inclusion GFP intensity threshold was a flexible threshold in that it changes for each cell and each frame since it relied on the mean fluorescence intensity of the cell body for the specific cell at the specific frame. If the ratio of inclusion area to cell body area was above 0.015, these cells were tracked as inclusion-positive cells. For survival analysis, a secondary filtering step was implemented to the tracked inclusion-positive cells to eliminate rounded dying cells with bright GFP signal that were incorrectly identified as inclusion-positive cells. The secondary filter consisted of an upper threshold of inclusion area in the soma and mean GFP fluorescence intensity. Tracked neurons with ratio of inclusion area to cell body area of less than 0.4 and cell body mean GFP fluorescent intensity below 150 were used for the survival analysis. For inclusion-negative cells, objects were detected as cell bodies if RFP fluorescence intensity was greater than 250 and length and width were 10–30 pixels (8–24 μm).

###### Cell and inclusion detection in spontaneous inclusion model (“E3K2chTracking”).

Detection and tracking of inclusion-positive neurons was based on GFP fluorescence, whereas that of inclusion-negative neurons was based on RFP fluorescence. For inclusion-positive cells, objects were identified as cell bodies if GFP fluorescence intensity was greater than 40 and length and width were 15–30 pixels (12–24 μm). Areas within cell bodies with fluorescent intensities of 160 or higher were identified as inclusions. If the ratio of inclusion area to cell body area was above 0.02, these cells were tracked as inclusion-positive cells. For inclusion-negative cells, objects were detected as cell bodies if RFP fluorescence intensity was greater than 180 and length and width were 10–40 pixels (8–32 μm).

###### Single-cell tracking and live/dead identification.

To track individual cells, a tentative cell identification (cell ID) number was assigned to each identified cell body at the beginning of the analysis. Neurons were tracking targets if they were detected at the analysis starting frame and were tracked for 5 frames or more. When cell area decreased by 50% compared to the previous frame or the fluorescence intensity decreased below the threshold, the cell was considered dead. The matching of individual cells between two time-frames was performed based on distance between each cell and changes in cell size and mean intensity. If the tracked cell merged with other cells, the tracking result was regarded as inaccurate, and the tentative cell ID was deleted. The final tracking ID number was renumbered automatically after tracking analysis.

###### Detection of neuritic inclusions in seeded inclusion model (“PFF2chTracking”).

Neurite-type inclusions in the seeded inclusion model were detected and measured using GFP fluorescence. Objects above length and fluorescence thresholds except for cell bodies were recognized as neurite-type inclusions. Objects that were 20–30 pixels long (16–24 μm) with GFP fluorescence intensity above 80 were identified as short and thick neurite-type inclusions. Objects more than 30 pixels long (≥24 μm) and with GFP fluorescent intensity above 60 were recognized as long neurite-type inclusions. Measurement of neurite-type inclusions was performed per well (population-based), instead of single-cell, since it was not always feasible to assign a neuritic inclusion to a cell soma (especially over long distances).

##### Single-cell survival tracking and image analysis (“Survival v2”)

Image analysis for automated neuron detection and survival tracking algorithm development was performed at Nikon Corporation. Survival curves were plotted with the “survival” package library in R. To detect the morphologic properties of the inclusion neuron models in culture, morphologic masks were designed to identify and quantify the different features, including cell body size, neurites, and intensity. After the masks for detection of each cell were optimized, the tracking was performed using the mask for cell body. The workflow used for this tracking is summarized in Figure S6E.

##### Neuron detection

Detection and tracking of neurons were based on RFP fluorescence. For survival analysis, a secondary filtering step was implemented to the tracked neurons to eliminate rounded dying cells with bright RFP signal. Tracked neurons with cell body mean GFP fluorescent intensity threshold (160) were used for the survival analysis.

##### Single-cell tracking and live/dead identification

To track individual cells, a tentative cell identification (cell ID) number was assigned to each identified cell body at the beginning of the analysis. Neurons were tracking targets if they were detected at the analysis starting frame and were tracked for 5 frames or more. When cell area decreased by 50% compared to the previous frame or the fluorescence intensity decreased below the threshold, the cell was considered dead. In addition, cells are regarded as dead cells at the time in which the number of nodes is 0. The number of nodes is measured as the counts of the number of thin linear objects connected to the cell body. The matching of individual cells between two time-frames was performed based on distance between each cell and changes in cell size and mean intensity. If the tracked cell merged with other cells, the tracking result was regarded as inaccurate, and the tentative cell ID was deleted. The final tracking ID number was renumbered automatically after tracking analysis.

###### Cell body detection.

To detect cell body area, binary images were first generated from original captured images by fluorescent intensity threshold. Secondly, binary images were processed with 5 cycles of erosion and dilation in the starting frame. After the start frame, binary images were processed with 4 cycles of erosion and dilation to refine the accuracy of the cell body area and especially to eliminate thin linear areas. Dilation adds pixels to the boundaries of objects in an image, whereas erosion removes pixels on object boundaries.

###### Neurite detection.

To detect the neurite area, original captured images were smooth-filtered to reduce spatial noise and to maintain the thin linear object information. Secondly, binary images were generated from smooth-filtered images by fluorescent intensity threshold. Lastly, binary images were eroded and dilated to refine the accurate neurite area.

#### LipidSpot live-cell staining and manual quantification

For live cell staining, LipidSpot 610 dye (Biotium, 70069) was added at 1:1000 dilution on DIV11 of NGN2 transdifferentiation together with preformed fibrils and twice every week to maintain lipid stain. For manual quantification, image frames were assessed using the CL-Quant software and findings were validated by an additional independent observer.

#### Live-cell compound treatments and imaging

For live-cell imaging, neurons were pretreated with LipidSpot 610 dye (Biotium, 70069) at 1:1000 dilution to visualize lipid-rich inclusions and imaged using an encoded stage on the Nikon TiE fluorescence microscope with a 20X Plan Apo dry objective and Andor Zyla 4.2P sCMOS camera (No binning, 200 MHz readout rate, 12 bit & Gain4 dynamic range). After 2 h, trifluoperazine and nortriptyline were added at the respective concentrations and images were acquired every 60 min for 4 h and every 24 h thereafter.

#### Postmortem brain tissue

The human ethics for this research with human postmortem tissue were approved by The University of Sydney Human Research Ethics Committee (HREC; 2020/707) under the project title “Determining the biological differences in alpha-synuclein in diverse synucleinopathies.” Transfer of familial E46K postmortem brain samples CES-BIOEF 2016–26 was approved by the CEIC-E (Comitéde Ética de la Investigación Clínica, Euskadi -IRB) on November 23, 2016, recorded in the proceedings Acta 10/2016.

#### Immunohistochemistry and immunofluorescence in postmortem brain

This study has used immunofluorescence in 16 sporadic PD cases (mean age 79 ± 6 years old, 13 males and 3 females, mean postmortem delay 18 ± 8 h), 2 A53T cases (51 ± 4 years old, both males, postmortem delay of 38 ± 15 h), 2 E46K cases (78 ± 20 years old, 2 females, mean postmortem delay 16 ± 13 h), and 8 non-neuropathological control cases (mean age 90 ± 7 years old, 4 males and 4 females, mean postmortem delay 28 ± 13 h). Other members of the E46K family have been described in.^114^ The E46K cases shown here are III-16 (A00) and IV-20 (A05).

Formalin-fixed cryostat frozen (FFFC) and formalin-fixed paraffin-embedded (FFPE) sections of the cingulate cortex (or available cortical sections from one of the two E46K cases) were cut at 8 μm with a rotary microtome (Thermo/Microm, HM325) and mounted on Series 2 adhesive microscope slides (Trajan Scientific Medical, AU) prior to immunostaining. FFPE sections were de-waxed and rehydrated with xylene and a series of graded ethanol before staining. The sections used for BODIPY and Nile Red are critically stained with a shorter deparaffinization step to preserve lipids: xylene for 3 min x 2 compared to the general xylene treatment of 7 min x 2.^115^ Each antibody was first tested with peroxidase immunohistochemistry to determine the optimal heat-induced antigen retrieval (HIAR). Tests showed that TE buffer (pH 9.0) was best for RhoA, citrate buffer (pH 6.0) was best for Rab8, citraconic anhydride buffer (0.05%, pH7.4) was best for beta-III tubulin, and TE buffer (pH 9.0) or CB buffer (pH 6.0) and additional formic acid treatment (70% concentration for 30 min) was best for αS staining. Immunofluorescence (IF) colabeling using combinations of primary antibodies (see Table S5) was then optimized to allow three antigens to be detected simultaneously. HIAR was performed in a programmable antigen retrieval cooker (Aptum Bio Retriever 2100, Aptum Biologics Ltd, UK) at a peak temperature of ~121°C, followed by gradual cooling for 2 h. After HIAR, sections were immersed in PBS with 0.1% sodium borohydride for 30 min on ice. After washing with PBS, sections were incubated with 100mM glycine in PBS for 30 min; then elimination of lipofuscin autofluorescence was performed with 0.1% Sudan Black in 70% ethanol for 30 min. After treatment with blocking buffer (containing 2% donkey serum and 1% BSA in PBS) for 1 h at room temperature, the slides were incubated with the cocktail of primary antibodies in blocking buffer at the appropriate dilutions for 48 h at 4^o^C, followed by their corresponding Alexa Fluor 488/568/647 secondary antibodies (dilution 1:250, see Table S5 for secondary antibodies) and 4′,6-diamidino-2-phenylindole (DAPI, Sigma D9542, 1 mg/mL) for 2 h at room temperature. To further quench autofluorescence, the fluorophore-labelled slides were finally treated with 10 mM CuSO_4_ in 50 mM ammonium acetate buffer (pH 5.0) for 1 h before being mounted with mounting medium (DAKO, cat# S3023) and sealing with nail polish. Negative controls were performed for each batch of staining by omitting either the primary or secondary antibodies and using control case sections. Sections were scanned using a confocal microscope (Nikon C2) at 40× objective for 3×3 large image mode (each AOI size is 887.14 × 887.14 μm^2^). To enhance the sampling of different types of aggregations, each section was scanned for 2–4 large images in the middle of the deep cortical layer where more αSyn pathology is located. Images were captured using a confocal microscope (Nikon C2) with parameters (laser power, gain, and offset) set based on the negative control and single channel labeling.

#### Correlative light and electron microscopy (CLEM) in iPSC-derived neurons and postmortem brain

This study performed CLEM in the substantia nigra and frontal cortex of 2 subjects with sporadic PD cases (aged 71 and 88 years old, male, postmortem delay 6 h and 4 h respectively) and 1 A53T case (51 ± 4 years old, male, postmortem delay of 38 ± 15 h). The sporadic cases were fixed at autopsy in 4% PFA and 0.1% glutaraldehyde in 0.15M cacodylate buffer pH 7.4 for 24 h. The A53T case was fixed at autopsy in 4% formalin, and post-fixed in 2.5% PFA and 2% glutaraldehyde for 24 h. 60 µm vibratome sections were prepared for EM as described in.^11^

iPSC-derived cortical neurons were seeded at day 7 (DIV7) at 0.6 million cells/well on ACLAR plastic discs coated with polyethyleneimine (PEI) (Sigma, 181978-100G) and laminin (Sigma, L2020) in a 12-well plate (Corning). At 25 days of differentiation (DIV25), iPSC-derived neurons were fixed in 0.1% glutaraldehyde (Electron Microscopy Sciences, 16220), 4% EM grade paraformaldehyde (Electron Microscopy Sciences, 15710), in 0.1 M cacodylate buffer (Electron Microscopy Sciences, 12300) in water for 1 h at room temperature. The coverslips were further processed by washing three times with 0.1M cacodylate buffer, incubating in 1% osmium tetroxide (OsO4) (Electron Microscopy Sciences 19150)/1.5% potassium ferrocyanide (KFeCN6) (Sigma P9387) for 30 min, followed by a series of three washes and 30 min incubation in 1% aqueous uranyl acetate (Electron Microscopy Sciences, 22400). Water was used to wash the neurons twice and they were dehydrated in 50%, 70%, 95% and twice in 100% alcohol. Neurons were embedded at 60°C for 2 days in TAAB Epon (TAAB, UK, T022).

CLEM was performed as described in.^11^ Briefly, serial 150 nm ultramicrotome sections of the resin embedded samples were cut and collected alternating on glass slides and EM grids. Immunohistochemistry was performed on the glass slides using the full-length αS antibody clone 42 (BD Biosciences, 610786) after formic acid pre-treatment in Tris-EDTA, pH 9.0, for 30 min at 95°C and a 1:100 dilution for 4 h at room temperature, or the p62 antibody SQSTM1 clone UMAB12 (VWR, ORIGUM500012) at a 1:100 dilution for 1 h at 37°C. Inclusions were detected with HRP-green (42lifesciences, S-99056-103). Light microscopy images were taken on a THUNDER 3D tissue imager (Leica). Immediately adjacent tissue sections mounted onto electron microscopy grids were imaged on a Philips CM100 transmission electron microscope at 80kV equipped with a TVIPS F416 camera.

#### Membrane yeast two-hybrid

Membrane yeast two-hybrid (MYTH) relies on a split ubiquitin system: a “bait” protein is fused to the N-terminal half of ubiquitin and directed to the ER membrane, while the “prey” protein (αS) is fused to the C-terminal half of ubiquitin. If the bait and prey proteins interact, the split ubiquitin is reconstituted, and cleavage by a deubiquitinase releases a transcription factor (TF) that controls expression of *HIS3*, thus allowing growth in media lacking histidine. The MYTH assay was conducted in two rounds, with 2 technical replicates per round. Interactions between 20 pairs of prey-bait proteins were included as positive controls (*EGFR* or *ATP13A2* as prey with 10 bait proteins each), and 188 prey-bait pairs were included as random controls in the second round. A total of 776 proteins were tested for interaction with αS. The MYTH method was as described in.^46^ Briefly, host yeast strain NMY51 (Dualsystems Biotech AG; genotype *MAT*a *his3delta200 trp1-901 leu2-3,112 ade2 LYS2::(lexAop)4-HIS3 ura3::(lexAop)8-lacZ (lexAop)8-ADE2 GAL4*) was transformed with bait vector pGBT3-STE harboring TF-Cub-Bait and prey vector pGPR3-N harboring Nub-Prey. Control construct (either Nubi-Ost1 or NubG-Ost1) was initially co-transformed with the bait vector to serve as one positive or negative control, respectively, to calibrate and optimize the assay conditions. Yeast harboring both bait and prey plasmids exhibited growth on synthetic complete (SC) medium depleted of Leu and Trp (SC-Leu-Trp). Protein interactions between bait and prey were detected by growth on SC-Leu-Trp-His supplemented with 10 mM of 3-amino-12,4-triazole (3AT) to test for *HIS3* reporter expression. Yeast growth phenotypes were scored 1–4 based on the size of the growth spot. Protein interactions were considered positive if both repeats got a score ≥1 (lenient interaction call), or if both scores were >1, or at least one score was 4 and the other ≥1 (stringent interaction call).

#### Proximity ligation assay (PLA)

For the PLA assay, the Duolink *In Situ* Orange Starter Kit Mouse/Rabbit (Sigma, DUO92102) was used. For permeabilization, 1X Saponin in PBS was incubated for 1 h at room temperature prior to blocking in Duolink Blocking Solution (80 μL/well; vortex well before use) for 1 h at 37°C. Primary antibodies were diluted in the Duolink Antibody Diluent corresponding to IF concentrations. For PLA negative controls, only one or no primary antibody was added. The plate was sealed tight with aluminum tape to prevent dehydration and incubated at 4°C overnight. Wash Buffers A and B were prepared to 1X concentration according to Duolink manufacturer protocol. In short, each wash buffer pouch was dissolved in 1 L autoclaved MilliQ water. The wash buffers were stored at 4°C shielded from light and were brought to room temperature before use. Following overnight primary antibody incubation, neurons were washed twice with 1X Wash Buffer A (200 μL/well) at room temperature for 5 min each. PLUS and MINUS PLA Probes were diluted 1:5 in pre-vortexed Duolink Antibody Diluent accordingly and incubated in the diluted PLA probe solution for 1 h at 37°C, followed by two subsequent washes with 1X Wash Buffer A at room temperature. 5X Ligation Buffer was thawed and diluted to 1X with Ultrapure distilled water (Invitrogen, 10977–015) immediately before use and Ligase was added to the freshly made 1X Ligation Buffer at 1:40 dilution. The neurons were incubated in the ligase solution for 30 min at 37°C. Following the ligation step, the neurons were washed twice with 1X Wash Buffer A at room temperature. 5X Amplification Buffer was thawed and diluted to 1X with UltraPure distilled water immediately before use, and Polymerase was added to the freshly made 1X Ligation Buffer at 1:80 dilution. The neurons were incubated in the ligase solution for 100 min at 37°C. After two washes with 1X Wash Buffer B (200 μL/well) at room temperature for 10 min each and an additional wash with 0.01X Wash Buffer B (diluted in UltraPure distilled water) at room temperature for 2 min, neurons were incubated with PBS-Hoechst solution (2000x fold dilution to PBS from commercial stock, Hoechst 33342, Invitrogen) at room temperature for 10 min, and further washed with 1X DPBS twice for 5 min each. The plate was sealed with aluminum tape and imaged immediately.

#### Genome-wide CRISPR-Cas9 screen in U2OS cells

A library of sgRNAs targeting 18,166 genes with 5 gRNAs per gene for a total of approximately 91,000 gRNAs was synthesized by oligo array and cloned into the lentiCRISPR V2 puro vector (Addgene plasmid # 52961). Sequences of sgRNAs were chosen based on performance in previous screens which identified essential genes.^116^ Cloning of the library was performed as follows. Oligo array synthesized oligos were PCR amplified and digested with BbsI. Digests were run on a 10% TBE gel and the 28 bp sgRNA band excised and gel purified. Digested sgRNAs were ligated into BsmBI digested lentiCRISPR V2 puro and ligated overnight at 16°C (250 ng digested lentiCRISPR V2, 1 μL Invitrogen T4 ligase, 4 μL 5X ligase buffer, 0.5 or 1 or 2 μL insert, to 20 μL with H_2_O). Ligated library was precipitated for 1 h at −80°C (20 μL ligation reaction +2 μL 3M sodium acetate pH 5.2, 0.6 μL 2 mg/mL glycogen, 50 μL 100% ethanol). Precipitated ligation product was resuspended in 5 μL of water and used to electroporate DH10beta bacteria (1 μL per 25 μL DH10beta electroporation reaction). Electroporated bacteria were grown in 1 μL SOC at 37°C for 1 h and then plated onto LB + ampicillin plates (500 μL of transformation per plate). Transformation efficiency was determined by counting colonies on dilution plates. Transformation should result in sufficient colonies for 20-30X coverage of the library (2–3 million colonies). Colonies were scraped into a 500 mL LB + ampicillin liquid culture and grown overnight at 30°C for pooled library maxiprep (Invitrogen, 2 columns per 500 mL culture). Pooled virus was prepared by transfecting 293T cells with the library plasmid pool with psPax2 and pMD2.G lentiviral packaging vectors. Viral supernatants were harvested at 48 and 72 h post transfection and concentrated with lenti-X concentrator solution (Clontech).

To determine the amount of virus to use in the infection, a titering experiment was performed in which 10-fold serial dilutions of the virus (10 μL, 1 μL, 0.1 μL, 0.01 μL, 0.001 μL) were used to infect cells seeded in a 6-well plate at the same seeding density as a 15-cm plate (i.e., 17-fold fewer cells based on the surface area difference between a 6-well plate and a 15-cm plate). Growth media supplemented with 2 μg/mL puromycin was added 1 day after infection and selection proceeded until the uninfected well was completely dead. The amount of virus resulting in 60–80% killing was recorded. This virus amount translates to a multiplicity of infection (MOI) of around 0.2–0.3 for the screen. The number of cells needed for the start of the screen depends on the size of the library to be screened. For a library of 40,000 gRNAs and a representation of 500 cells/gRNA, 20 million cells are required per replicate. For screening in triplicate, this means that 60 million cells are required. A low MOI is used to ensure that there is only 1 gRNA per cell, thus 3–5 times as many cells as virus are required. Taken together, a library of 40,000 gRNAs at a representation of 500 in triplicate requires 180–300 million cells at the start of the screen.

U2OS cell lines were expanded to 27×15-cm plates/line at 10 million cells/plate for a total of 270 million cells for the start of the screen. U2OS cells were passaged by washing adherent cells with 1X DPBS, incubating with Trypsin for 5 min at 37°C, centrifuging for 5 min at 300 g, aspirating the supernatant, resuspending the cell pellet in growth media (McCoy’s 5A, 10% fetal bovine serum (FBS), penicillin-streptomycin), and plating at the desired cell density. Cells were infected with a gRNA/Cas9 lentivirus library at low MOI (0.2) with a representation of 500 cells/gRNA in triplicate, followed by puromycin (2 μg/mL) selection for 1 week, or until an uninfected control plate completely died. An initial cell pellet (50 million cells) was harvested as day 0 after expansion of the puromycin-selected cells to the appropriate scale to begin the screen (100 million cells/line). The remaining 50 million cells were replated and treated with doxycycline (100 ng/mL) to induce αS. Cell pellets were harvested 7 days (7 population doublings) and 14 days (14 population doublings) after doxycycline induction.

Genomic DNA was isolated from the day 0, 7, 14 cell pellets by phenol:chloroform extraction. Briefly, the cell pellet is resuspended in TE (10 mM Tris pH 8.0, 10 mM EDTA) to a final concentration of 2–10 million cells/mL of TE and combined with 0.5% SDS and 0.5 mg/mL Proteinase K. The suspension was incubated at 55°C overnight, with shaking/inverting the cell suspension over the course of 1 h to ensure complete digestion. Next, 0.2 M NaCl was added, followed by phenol chloroform extraction in phase lock gel tubes. Equal parts of phenol:chloroform and sample were mixed in phase lock gel tubes, shaken for 1 min to extract, then centrifuged for 5 min. The DNA aqueous phase (top layer) was subsequently chloroform extracted by mixing equal parts with chloroform, shaken for 1 min, and centrifuged for 5 min. The tubes were incubated with the caps open for 1 h at 50°C to evaporate the chloroform. Samples were treated with 25 μg/mL RNase A overnight at 37°C, then extracted with phenol:chloroform and chloroform as described above. DNA was precipitated with ethanol overnight at −20°C, or for 3 h at −80°C. Next, 1/10 v/v 3M sodium acetate pH 5.2 and 2 volumes 100% ethanol was added and the mixture centrifuged for 30–45 min at 4500 rpm at 4°C. The DNA pellet was washed once with 70% ethanol and transferred to an Eppendorf tube, followed by two more washes with 70% ethanol. The DNA pellet was dried at 37°C for 10–20 min, then resuspended in 1 mL EB/TE by incubating at 55°C. gRNAs were PCR amplified with barcoded primers for sequencing on an Illumina NextSeq 500. Sequencing reads were aligned to the initial library and counts were obtained for each gRNA. MAGeCK and edgeR were used to calculate *p*-values, FDRs, and log_2_ fold changes for comparison between the day 14 and day 0 samples for each cell line.

A list of 147 top hits from the CRISPR screen was compiled by applying the following thresholds in MAGeCK and edgeR analyses: log_2_ fold-change ≤ −1 or log_2_ fold-change ≥1 and FDR ≤0.1 for pairwise comparisons between SNCA-3K-sfGFP, SNCA-WT-sfGFP, and sfGFP fold-change. For the heatmap in Figure S9C, arbitrary cutoffs were used to categorize the data. The following cutoffs were used for enhancers of SNCA-3K-sfGFP: genes with log_2_ fold-change differential between SNCA-3K-sfGFP and SNCA-WT-sfGFP < −0.7 (dropout in 3K condition), SNCA-3K-sfGFP FDR <0.25 for significance cutoff, and log_2_ fold-change for WT < 1 (to avoid genes that make WT cells grow better). The following cutoffs were used for suppressors of SNCA-3K-sfGFP: log_2_ fold-change differential between SNCA-3K-sfGFP and SNCA-WT-sfGFP >1 to indicate more enrichment in SNCA-3K-sfGFP condition, and SNCA-3K-sfGFP FDR <0.2 for significance cutoff. The list of 147 top hits was used in the PANTHER Gene Ontology (GO) enrichment analysis.

#### ROS/MAP differential expression analysis

##### ROS/MAP study participants

Postmortem data analyzed in this study were gathered as part of the Religious Orders Study and Memory and Aging Project (ROS/MAP),^117–119^ two longitudinal cohort studies of the elderly, one from across the United States and the other from the greater Chicago area. All subjects were recruited free of dementia (mean age at entry = 78 ± 8.7 (SD) years) and signed an Anatomical Gift Act allowing for brain autopsy at time of death. Written informed consent was obtained from all ROS/MAP participants and study protocols were approved by the Rush University Institutional Review Board.

##### Study participants neuropathological evaluation

All subjects’ brains were examined postmortem by a board-certified neuropathologist blinded to clinical data. Brains were removed in a standard fashion as previously described.^120^ Each brain was cut into 1 cm coronal slabs. Slabs from one hemisphere, and slabs from the other hemisphere not designated for rapid freezing, were fixed for at least three days in 4% paraformaldehyde. Tissue blocks from eight brain regions were processed, embedded in paraffin, cut into either 6 μm or 20 μm sections, and mounted on glass slides. Lewy body pathology was measured by immunostaining, as described.^121^ Briefly, Lewy bodies in substantia nigra and cortex were separately identified, with only intracytoplasmic Lewy bodies indicating positive staining. To simplify the staging of pathology, the McKeith criteria^122^ were modified such that nigral predominant Lewy body pathology included cases with Lewy bodies in the substantia nigra without evidence of Lewy bodies in the limbic or neocortical regions. Limbic-type LBD included cases with either anterior cingulate or entorhinal positivity without neocortical Lewy body pathology. Neocortical-type Lewy body pathology required Lewy bodies in either midfrontal, temporal, or inferior parietal cortex with either nigral or limbic positivity, but often with both. Based on this, each subject was assigned to one of four mutually exclusive categories: 0 = no, 1 = nigral-predominant, 2 = limbic-type or 3 = neocortical-type Lewy body pathology. Further descriptions of clinical and pathological outcomes are available at the Rush Alzheimer’s Disease Center Research Resource Sharing Hub (https://www.radc.rush.edu).

##### RNA sequencing data processing and quality control

The pipeline for sequencing has been described previously.^71^ RNA sequencing on DLPFC tissue was carried out in 13 batches within three distinct library preparation and sequencing pipelines. All samples were extracted using Qiagen’s miRNeasy mini kit (Qiagen, 217004) and the RNase free DNase Set (Qiagen, 79254), and quantified by Nanodrop and quality was evaluated by Agilent Bio-analyzer. Full details on these methods are available on the AMP-AD knowledge portal (syn3219045). Briefly, for pipeline #1, The Broad Institutes’s Genomics Platform performed RNA-Seq library preparation using the strand specific dUTP method^123^ with poly-A selection.^124^ Sequencing was performed on the Illumina HiSeq with 101bp paired-end reads and achieved coverage of 150M reads of the first 12 samples. The remaining samples were sequenced with coverage of 50M reads. For pipeline #2, RNA sequencing libraries were prepared using the KAPA Stranded RNA-Seq Kit with RiboErase (kapabiosystems) in accordance with the manufacturer’s instructions. Sequencing was performed on the Illumina NovαSeq6000 using 2 × 100bp cycles targeting 30 million reads per sample. For pipeline #3, RNA was extracted using Chemagic RNA tissue kits (PerkinElmer, CMG-1212) on a Chemagic 360 instrument. 500 ng total RNA was used as input for sequencing library generation and rRNA was depleted with RiboGold (Illumina, 20020599). A Zephyr G3 NGS workstation (PerkinElmer) was utilized to generate TruSeq stranded sequencing libraries (Illumina, 20020599). Sequencing was performed on a NovαSeq 6000 (Illumina) at 40-50M reads (2×150 bp paired end).

##### Differential expression analysis

All analyses were performed in R (R-Core-Team, 2014) (v3.6.3) using the same process as described.^71^ Prior to calculating differential expression, a brain cell type-corrected expression matrix was generated for DLPFC on voom-transformed expression values. This matrix was used as input for differential expression analyses. Cell type proportions were estimated using the Brain Cell Type Specific Gene Expression Analysis (BRETIGEA)^125^ package in R. Human marker genes (*n* = 50 per set) from^126^ were used in the application of a validated singular value decomposition method^127^ (the “adjustBrainCells” function). The limma package ‘lmFit’ function was then used to model cell type-corrected log_2_(expected counts) as a linear function of Lewy body pathological stage, including batch, study, biological sex, age at death, PMI, median coefficient of variation for coverage values of the 1000 most highly expressed genes, % of aligned bases mapping to ribosomal RNA, % coding bases, % UTR bases, log(estimated library size), log(passed filter aligned reads), median 5′ to 3′ bias, % of passed filter reads aligned, % read duplicates, median 3′ bias, and % of intergenic bases as covariates. Robust linear modeling was used (using Huber M estimators^128^), allowing for a high number (10,000) of iterations to reach convergence. Significance for effects of pathological variables on gene expression were determined using empirical Bayes moderation.

##### Rank-based enrichment of U2OS CRISPR-Cas9 screen and MYTH prioritized gene sets

The full set of gene-wise differential expression summary statistics were ranked based on moderated t-statistics. Rank-based Gene Set Enrichment Analysis (GSEA) was performed using the ‘GSEA’ function in the R ‘clusterProfiler’ package (https://bioconductor.org/packages/release/bioc/html/clusterProfiler.html) and three custom gene sets derived from functional screening experiments described above. The gene sets included only genes that were present in the DLPFC transcriptomic data following quality control (MYTH *n*_genes_ = 269; CRISPR *n*_genes_ = 138; combined *n*_genes_ = 401). The GSEA algorithm uses a running sum statistic, the normalized enrichment score (NES), which deviates from 0 over the full distribution of ranked gene association statistics if members of a candidate gene set are found clustered toward the start or end of the distribution. This statistic also accounts for gene set sizes and its significance is determined by permuting gene ranks to calculate a null distribution. An NES greater than 0 indicates a GO group enriched for higher expression with increasing Lewy body pathology, and an NES less than 0 indicates a group enriched for lower expression with increasing Lewy body pathology. *p* values in these comparisons are two-sided and uncorrected, corresponding to the NES-based test for each gene set independently.

### QUANTIFICATION AND STATISTICAL ANALYSIS

Unless otherwise stated, plots and statistical tests were generated by GraphPad Prism or R. Unpaired t-test was used to compare two groups. For multiple t-test comparisons, multiple hypothesis corrections were performed as indicated in the respective figure legends. Log rank test was used to compare the survival distributions of two groups. For image analysis, FIJI distribution of ImageJ, CL-Quant, and ImageStudio software was used. For quantification of αS pS129 area, a custom macro was written. CUX1 expression was determined across Hoechst+ intact nuclei. To exclude fragmented nuclei indicating dead cells, only Hoechst(+) objects with a minimum area size of 60μm^2^ were selected. To quantify Vglut1(+)/Tuj1(+) neurons, all intact nuclei (fragmented nuclei excluded) were quantified using an ImageJ macro, and nuclei without a surrounding Vglut1/Tuj1 signal were subtracted to calculate the percentage of Vglut1(+)/Tuj1(+) neurons. Unless noted otherwise, all error bars reflect standard deviation of the mean.

## Supplementary Material

MMC8

MMC7

MMC6

MMC5

MMC4

MMC3

MMC2

MMC9

MMC10

MMC1

## Figures and Tables

**Table:** KEY RESOURCES TABLE

REAGENT or RESOURCE	SOURCE	IDENTIFIER
Antibodies		
Anti-Alpha-Synuclein	BD Biosciences	Cat. # 610786; RRID:AB_398107
Anti-Alpha-Synuclein	R&D	Cat. # AF1338; RRID:AB_2192798
Anti-Alpha-Synuclein (4B12)	Thermo Fisher	Cat. # MA1-90346; RRID:AB_1954821
Anti-Alpha-Synuclein (clone SOY1)	Sigma Aldrich	Cat. # MABN1818
Anti-Alpha-Synuclein (Syn1)	BD Transduction Laboratories	Cat. # 610787; RRID:AB_398108
Anti-AQP4, clone CL0178	Sigma	Cat. # AMAB90537; RRID:AB_2665579
Anti-AQP4	EMD Millipore	Cat. # AB3594; RRID:AB_91530
Anti-ArpC2	Abcam	Cat. # ab133315
Anti-Beta-Tubulin III, clone TUJ1	Stem Cell Technologies	Cat. # 60052
Anti-Brn2, clone D2C1L	Cell Signaling	Cat. # 12137; RRID:AB_2797827
Anti-Ctip2	Abcam	Cat. # ab18465; RRID:AB_2064130
Anti-Cux1 (Anti-Protein CASP; 2A10)	Abcam	Cat. # ab54583; RRID:AB_941209
Anti-GAPDH	EMD Millipore	Cat. # MAB374; RRID:AB_2107445
Anti-GAPDH	Thermo Fisher	Cat. # MA5-15738; RRID:AB_10977387
Anti-GFAP	Dako, Agilent Technologies	Cat. # Z0334; RRID:AB_10013382
Anti-GFP	Abcam	Cat. # ab6556; RRID:AB_305564
Anti-GFP	Roche Diagnostics	Cat. # 11814460001; RRID:AB_390913
Anti-GFP	Rockland	Cat. # 600-101-215; RRID:AB_218182
Anti-LC3A/B	Cell Signaling	Cat. # 4108; RRID:AB_2137703
Anti-p62	EMD Millipore	Cat. # MABN130
Anti-p62 Lck ligand	BD Bioscience	Cat. # 610833; RRID:AB_398152
Anti-p62 SQSTM1 clone UMAB12	VWR	Cat. # ORIGUM500012
Anti-Phospho-Ser-129	Abcam	Cat. # ab51253; RRID:AB_869973
Anti-Rab5, clone C8B1	Cell Signaling	Cat. # 3547; RRID:AB_2300649
Anti-Rab8	BD Biosciences	Cat. # 610844; RRID:AB_398163
Anti-Rab11, clone D4F5	Cell Signaling	Cat. # 5589; RRID:AB_10693925
Anti-Rab11A	Thermo Fisher	Cat. # 71-5300; RRID:AB_2533987
Anti-Rab35	ProteinTech	Cat. # 11329-2-AP; RRID:AB_2238179
Anti-RhoA, clone 67B9	Cell Signaling	Cat. # 2117; RRID:AB_10693922
Anti-RhoA	Santa Cruz	Cat. # sc-166399; RRID:AB_2269522
Anti-S100b	Sigma	Cat. # S2532; RRID:AB_477499
Anti-Tbr1	Abcam	Cat. # ab183032; RRID:AB_2936859
Anti-TUJ1	BioLegend	Cat. # 801201; RRID:AB_2313773
Anti-Ubiquitin (FK2)	EMD Millipore	Cat. # ST1200; RRID:AB_10681625
Anti-Ubiquitin	Abcam	Cat. # ab7780; RRID:AB_306069
Anti-VGLUT1	Synaptic Systems	Cat. # 135303; RRID:AB_887875
Anti-Vimentin	EMD Millipore	Cat. # CBL202; RRID:AB_93387
BODIPY 493/503	Thermo Fisher	Cat. # D3922
LipidSpot 610	Biotium	Cat. # 70069
Total OXPHOS Human WB Antibody Cocktail	Abcam	Cat. # ab110411; RRID:AB_2756818
Deposited data		
Comprehensive key resources table	This paper	Zenodo: https://doi.org/10.5281/zenodo.12549027
Tabular data relating to graphs	This paper	Zenodo: https://doi.org/10.5281/zenodo.12549027
U2OS pB-SNCA-3K CRISPR-Cas9 genetic screen raw sequencing counts	This paper	Mendeley Data: https://doi.org/10.17632/3jgy6bw5zz.1
Software and algorithms		
Code relating to figures	This paper	Zenodo: https://doi.org/10.5281/zenodo.12574231
U2OS pB-SNCA-3K CRISPR-Cas9 code for screen analysis	This paper	Mendeley Data: https://doi.org/10.17632/t3fr85mfzt.1
Nikon BioStation CT “PFF2chTracking”	This paper	Zenodo: https://doi.org/10.5281/zenodo.12662455
Nikon BioStation CT “E3K2chTracking”	This paper	Zenodo: https://doi.org/10.5281/zenodo.12662183
Nikon BioStation CT “Survival v2”	This paper	Zenodo: https://doi.org/10.5281/zenodo.12662477

## Highlights

- iPSC toolbox to rapidly induce CNS cells with expression of aggregation-prone proteins
- Distinct α-synuclein inclusion subtypes have differing effects on neuron viability
- Key proteins, such as RhoA, and neutral lipids mark especially toxic inclusion subtypes
- Findings in inclusionopathy models validate in postmortem synucleinopathy brain
