# Rapid iPSC inclusionopathy models shed light on formation, consequence, and molecular subtype of α-synuclein inclusions

**DOI:** 10.1016/j.neuron.2025.01.018

**Published:** 2025-02-01

**Authors:** Isabel Lam, Alain Ndayisaba, Amanda J. Lewis, YuHong Fu, Giselle T. Sagredo, Anastasia Kuzkina, Ludovica Zaccagnini, Meral Celikag, Jackson Sandoe, Ricardo L. Sanz, Aazam Vahdatshoar, Timothy D. Martin, Nader Morshed, Toru Ichihashi, Arati Tripathi, Nagendran Ramalingam, Charlotte Oettgen-Suazo, Theresa Bartels, Manel Boussouf, Max Schäbinger, Erinc Hallacli, Xin Jiang, Amrita Verma, Challana Tea, Zichen Wang, Hiroyuki Hakozaki, Xiao Yu, Kelly Hyles, Chansaem Park, Xinyuan Wang, Thorold W. Theunissen, Haoyi Wang, Rudolf Jaenisch, Susan Lindquist, Beth Stevens, Nadia Stefanova, Gregor Wenning, Wilma D.J. van de Berg, Kelvin C. Luk, Rosario Sanchez-Pernaute, Juan Carlos Gómez-Esteban, Daniel Felsky, Yasujiro Kiyota, Nidhi Sahni, S. Stephen Yi, Chee Yeun Chung, Henning Stahlberg, Isidro Ferrer, Johannes Schöneberg, Stephen J. Elledge, Ulf Dettmer, Glenda M. Halliday, Tim Bartels, Vikram Khurana

In the originally published version of the paper, we, the authors, found some errors in Table S1. The SNCA (A53T>T) iPSC lines from Yumanity Therapeutics/BWH are female but are indicated as male in column C (“Gender”). This applies to tab “Non-transgenic lines,” rows 11–15, and tab “Transgenic lines,” rows 3, 7–9, 12, 15, 16, 21, and 29. In addition, under tab “Non-transgenic lines,” SNCA-triplication-2 clones in column B (“iPSC line”), rows 20–23, were wrongfully denoted as “SNCA-triplication-1.” This has now been corrected online. The authors apologize for the error.

