# Supplementary material for: Rapid iPSC inclusionopathy models shed light on formation, consequence, and molecular subtype of α-synuclein inclusions
Source: Neuron. Author manuscript; Available in PMC 2024 Sep 6. (PMC11377155; doi:10.1016/j.neuron.2024.06.002)
Supplement: MMC1 [file NIHMS2014139-supplement-MMC1.pdf]

Neuron, Volume *112*

Supplemental information

Rapid iPSC inclusionopathy models shed light on formation, consequence, and molecular subtype of α-synuclein inclusions

Isabel Lam, Alain Ndayisaba, Amanda J. Lewis, YuHong Fu, Giselle T. Sagredo, Anastasia Kuzkina, Ludovica Zaccagnini, Meral Celikag, Jackson Sandoe, Ricardo L. Sanz, Aazam Vahdatshoar, Timothy D. Martin, Nader Morshed, Toru Ichihashi, Arati Tripathi, Nagendran Ramalingam, Charlotte Oettgen-Suazo, Theresa Bartels, Manel Boussouf, Max Schäbinger, Erinc Hallacli, Xin Jiang, Amrita Verma, Challana Tea, Zichen Wang, Hiroyuki Hakozaki, Xiao Yu, Kelly Hyles, Chansaem Park, Xinyuan Wang, Thorold W. Theunissen, Haoyi Wang, Rudolf Jaenisch, Susan Lindquist, Beth Stevens, Nadia Stefanova, Gregor Wenning, Wilma D.J. van de Berg, Kelvin C. Luk, Rosario Sanchez-Pernaute, Juan Carlos Gómez-Esteban, Daniel Felsky, Yasujiro Kiyota, Nidhi Sahni, S. Stephen Yi, Chee Yeun Chung, Henning Stahlberg, Isidro Ferrer, Johannes Schöneberg, Stephen J. Elledge, Ulf Dettmer, Glenda M. Halliday, Tim Bartels, and Vikram Khurana

Supplementary Figure 1: Generation of targeted inducible *SNCA* transgene at safe harbor (*AAVS1*) locus, related to Figure 1. (**A**) Immunofluorescence (IF) of DIV21 H9/pi-As (induced via pB-NFIB-SOX9) and parental hESCs with S100β and two different AQP4 antibodies (CL0178 or ab3594). (**B**) IF of DIV21 H1/pi-As (induced via pB-NFIB) with S100β and GFAP antibodies. (**C**) Neuron-astrocyte co-culture of CORR/pi-Ns (DIV25) and H1/pi-As (pB-NFIB) labelled with MAP2 and S100β. (**D**) Schematic of TALEN-mediated gene editing in WIBR-3, clone 38 hESC line with two constructs for doxycycline (Dox)-dependent regulation, one containing the *M2rtTA* reverse tetracycline transactivator, and the other containing the $P_{TRE\text{-}Tight}$-*SNCA* transgene (TRE, tetracycline response element). Inset: Western blot showing doxycycline dose-response in hESCs with *SNCA* transgene integrated at *AAVS1* locus. Ctrl, control sample (no Dox). (**E**) Southern blot of WIBR-3 clones to confirm integration of *SNCA-mK2* construct (4.11 kb) at the second *AAVS1* locus (subclones 1 and 3; c1, c3), compared to single-targeted parental clone 38/16 carrying one P_{CAGGS}-*M2rtTA* (3.6 kb) and one wild-type (WT; 5.4 kb) allele. T, correctly targeted allele. (**F**) Comparison of *SNCA-mK2-AAVS1* transgene expression efficiency via conventional differentiation (terminal differentiation DIV21, 5-week exposure to doxycycline) or NGN2 transdifferentiation, with 11-day exposure to doxycycline (DIV11). Representative image from >3 separate neuronal differentiations. (**G**) c-$N^{GFP\text{-}AAVS1}$ differentiated via spin EB method for neural induction and exposed to doxycycline from DIV8 for 3 weeks demonstrate lack of colocalization of GFP with astroglial marker GFAP (left). GFP signal is exclusively neuronal (colocalized with Tuj1; right). (**H**) Comparison of transgene expression efficiency by targeting to *AAVS1*, *STMN2*, and pB random integration in pi-Ns. Right: Quantification of transgene-expressing cells from IF of NGN2 transdifferentiation in the different transgene models. Three independent replicates across 1 (*AAVS1*) and 2 (pB and *SMTN2*) neuronal differentiations. (**I**) IF and quantification of CUX1 and TBR1 confirm superficial cortical neuron identity (layer II/III). (**J**) IF and quantification of VGLUT1 and βIII Tubulin signals in neurons transdifferentiated from hESC with *SNCA-mK2* transgene at *AAVS1* locus. Three independent replicates from one neuronal differentiation are shown. (**K**) Left: *STMN2* mRNA expression is neuron-specific (source: Brain RNA-Seq browser). Right: SNCA-GFP transgene integration (with A53T or ΔNAC mutations) at *STMN2* locus does not alter *STMN2* expression as measured by qPCR. (**L**) Summary table of number of iPSC lines with pB integration, *AAVS1,* or *STMN2* targeted integration, and the proteotoxic transgene that was targeted.

Supplementary Figure 2: Analysis of αS PFFs used in seeding experiments, related to Figure 2. (**A**) IF and quantification of VGLUT1 and βIII Tubulin in pi-$N^{SNCA\text{-}4/2/0\text{-}copy}$ and pi-$N^{SNCA\text{-}pB}$ models. (**B**) IF and quantification for CUX1 and TBR1 confirms cortical neuronal identity (layer II/III). (**C**) Western blot of αS in CORR and isogenic CORR/pi-$N^{SNCA\text{-}pB}$ neurons versus postmortem ("p.m.") brain lysate (frontal cortex) from 3 MSA cases, 4 LBD cases, and 3 CNS-unaffected controls (healthy controls, "HC"). Related to **Fig. 2D**. (**D**) Negative staining electron microscopy (EM) of recombinant wild-type αS PFFs post-fibrillization and post-sonication. (**E**) Quantification of fibril length in panel D. (**F**) Quantification of pS129 signal in unseeded pi-$N^{SNCA\text{-}4/2/0\text{-}copy}$ and pi-$N^{SNCA\text{-}pB}$ models; 3-5 independent replicates across 3 separate neuronal differentiations. ns, not significant. (**G**) EM of MSA and LBD brain-derived PFFs post-SAA. (**H**) Proteinase K digestion (1μg/mL) of MSA and LBD brain-derived fibrils pre- and post-SAA.

Supplementary Figure 3: Mitochondrial and lysosomal function in seeded inclusionopathy model, related to Figure 3. (**A**) Left: αS western blot in seeded inclusionopathy models. Right: Quantification of 3 independent replicates across 3 separate neuronal differentiations. One-way ANOVA + Tukey's multiple comparison test: *p<0.05; ****p<0.0001. (**B**) IF for pS129 in unseeded pi-$N^{A53T\text{-}pB}$ models; quantification of 2-3 independent replicates across 3 separate neuronal differentiations. (**C**) Seahorse assay in PFF-seeded and unseeded, sfGFP-tagged (top) and

untagged (bottom) pi-Ns (DIV20). "Max Resp.", Maximal Respiration. (**D**) Oxygen consumption rate (OCR) across different conditions taken at baseline (0-18 min of the Seahorse assay) or at maximal respiration (36-54 min of the Seahorse assay). (**E**) Mitochondrial subunit assay in seeded inclusion model and control lines (DIV56). Right: Western blot quantification normalized to TOMM20. (**F**) Autophagic flux assay in seeded and unseeded, sfGFP-tagged and untagged pi-Ns (DIV56). (**G**) Analysis of autophagic flux based on quantification of western blots in panel F. One-way ANOVA + Tukey's multiple comparison test for panels D, E, G: **p<0.01, ***p<0.001, ****p<0.0001; ns, not significant.

Supplementary Figure 4: Characterization of transgenic A53T inclusionopathy models, related to Figures 3 and 4. (**A**) IF of SNCA-A53T-sfGFP transgene expression in MAP2(+) neurons after knock-in at *STMN2* locus. (**B**) IF and quantification of VGLUT1 and βIII Tubulin in pi-N^{STMN2} model. (**C**) IF and quantification of pi-N^{STMN2} model for CUX1 and TBR1. (**D**) Quantification of pS129 area from IF in seeded inclusion models compared to respective ΔNAC control lines; 2-4 independent replicates across 3 separate neuronal differentiations. (**E**) Confocal microscopy examples of pS129(+) soma-type (S) and neurite-type (N) inclusions. (**F**) IF for p62 and ubiquitin in neurite-type inclusions of the seeded inclusionopathy model. (**G**) Left: Low magnification view of ubiquitin and pS129 staining in seeded untagged pi-$N^{A53T\text{-}pB}$ model. Right: Representative examples of soma-type or neurite-type inclusions and their co-staining with ubiquitin. (**H**) Same as (G), except immunostainings are with p62 and pS129. (**I**) IF for ubiquitin and p62 in ΔNAC control neurons. (**J**) Quantification of inclusions positive for pS129, p62 and ubiquitin at different DIV in untagged seeded inclusionopathy model; 3 independent replicates across 3 separate neuronal differentiations. (**K**) Cross-sectional analysis of seeded inclusionopathy model (DIV25) to evaluate association of p62- and ubiquitin-immunopositive inclusions with intact nuclei (presumed live cell) versus fragmented nuclei (presumed dead cell) (quantified in **Fig. 4F**). (**L**) Same analysis as in (K) but in untagged model; 3 independent replicates across 3 separate neuronal differentiations. Paired t-test for panel L: *p<0.05; **p<0.01. One-way ANOVA + Tukey's multiple comparison test for panels D and J: ****p<0.0001. ns, not significant.

Supplementary Figure 5: Frequency of LipidSpot(+) inclusions in seeded inclusionopathy model decreases upon seeding with PFFs and treatment with previously reported PD drug candidates, related to Figures 4 and 5. (**A**) GFP-immunogold electron microscopy (EM) of neurite-type inclusion in PFF-seeded pi-$N^{A53T\text{-}sfGFP\text{-}pB}$ model. (**B**) Correlative light and electron microscopy (CLEM) of untagged seeded pi-$N^{A53T\text{-}pB}$ model. (**C**) CLEM for pS129 in substantia nigra of familial PD brain (A53T). (**D**) Left: Lipid-rich LipidSpot(+) inclusions in untagged seeded inclusion model. Top right graph: Quantification of pS129 signal/cell in pi-$N^{A53T\text{-}pB}$ with and without recombinant PFF treatment. Bottom right graph: Quantification of LipidSpot(+) inclusion-bearing neurons with and without recombinant PFF treatment. 3-4 independent replicates across 3 separate neuronal differentiations. (**E**) LipidSpot staining in pi-$N^{\Delta NAC\text{-}pB}$ and pi-$N^{sfGFP\text{-}pB}$ control neurons. (**F**) Left: High magnification images pre- (T=0min) and post-treatment (T=60min) of LipidSpot(+) inclusions with nortriptyline ("NOR"). Right: Quantification at T=60min post-treatment (3 independent replicates per treatment condition). (**G**) Manual quantification of cell survival every 24 h for five days to assess dose-dependent cellular toxicity associated with trifluoperazine ("TFP") and nortriptyline ("NOR") treatment. One-way ANOVA + Tukey's multiple comparison test for panels D and F: **p<0.01; ****p<0.0001.

Supplementary Figure 6: Characterization of spontaneous inclusionopathy model and development of automated single-cell survival tracking, related to Figure 6. (**A**) Confocal microscopy of soma-type (S) and neurite-type (N) inclusions immunostained for pS129, p62 and

ubiquitin. (**B**) Single-cell inclusion tracking algorithms (PFF2chTracking, E3K2chTracking) automatically detect and track neurons and inclusions, and live/dead status. Individual wells were captured with 5x5 stitched images with phase, RFP and GFP channels. 1. Parameters for automated neuron and inclusion detection. 2. Parameters for detection of neuron death. Micrographs show frame at which a GFP(+) inclusion-containing neuron was called dead by algorithm. 3. Output of the algorithm. (**C**) Example of detection of an RFP(+) neuron and its death at frame 34. (**D**) Accuracy of automated detection and survival of inclusion(+) and inclusion(-) neurons in seeded and spontaneous inclusion models using PFF2chTracking and E3K2chTracking for each respective model. Denominator in parenthesis indicates number of neurons evaluated. "Detection": Neuron was identified correctly as a live, trackable cell. "Survival": Neuron was identified as dead at the correct frame. (**E**) Workflow of single-cell survival and neurite detection algorithm (Survival v2) that tracks neurons based on RFP signal.

Supplementary Figure 7: Automated single-cell inclusion survival tracking of seeded inclusionopathy model, related to Figure 6. (**A**) BioStation CT images of PFF-seeded and unseeded pi-Ns. Red arrows: Neurite-type inclusions. (**B**) Example of neurite-type inclusion mask generated by the algorithm. (**C**) Timeline of neuron culturing and imaging for inclusion survival tracking in seeded inclusionopathy model. Inclusion(+) neurons were tracked with GFP, and inclusion(-) neurons were tracked with RFP. (**D**) Survival tracking at the cellular level. Inclusion status was ignored in this analysis. n, number of neurons tracked. Top: Example of RFP(+) tracked pi-N$^{A53T\text{-}sfGFP\text{-}pB+PFF}$. White arrow: Timeframe at which neuron dies. (**E**) Kaplan-Meier curve of inclusion(+) (soma-type) and inclusion(-) seeded pi-Ns. Top: Examples of 3 inclusion(+) neurons that were tracked. (**F**) Automated detection of GFP(+) neurite-type inclusions and cumulative length per well in seeded and unseeded pi-Ns. Error bars: Mean±SD from 3 wells. Panels D, E, F are representative of 2 neuronal differentiations.

Supplementary Figure 8: Colocalization of Rab markers with inclusion subtypes, related to Figure 7. (**A**) Top: IF for Rab5 and Rab11 in inclusionopathy models. Bottom: IF for Rab5, Rab8 and Rab11 in ΔNAC and sfGFP control pi-Ns. (**B**) Low-magnification micrographs of Rab8-pS129 PLA in seeded pi-Ns. Negative controls exclude primary antibodies (middle) or Rab8 antibody (bottom). (**C**) Rab8-pS129 PLA in control neurons. (**D**) Rab8 signal in αS(+)/BODIPY(+) inclusions in anterior cingulate cortex in postmortem brain. (**E**) BODIPY and Nile red stains in neighboring sections of mouse FFFC white adipose tissue (WAT) and human sporadic PD brain FFFC tissue. (**F**) BODIPY and Nile red stains of neighboring FFFC and FFPE sections of sporadic PD (n=4) and familial PD (n=2) cases. The brightest signals are from intracellular neutral lipids. (**G**) Immunostaining of αS(+) inclusions co-localizing with BODIPY and Nile Red in FFFC sections of sporadic PD (n=4) and familial PD (n=2) brain. Arrowheads, examples of αS(+) inclusions and respective p62, BODIPY or Nile Red colocalization.

Supplementary Figure 9: Convergence of CRISPR screen and MYTH on RhoA, related to Figure 8. (**A**) Left: Western blot to assess transgene expression in U2OS cells. Center: Incucyte cell culture confluence analysis of U2OS pB lines. Right: Inclusion GFP signal in SNCA-3K-sfGFP or SNCA-3K-ΔNAC-sfGFP cells. (**B**) Left: U2OS cells expressing pB-SNCA (A53T or A53TΔNAC) induced with 100ng/mL doxycycline and treated with increasing amounts of recombinant A53T PFFs (1, 5 or 10 μg/mL). Right: Inclusion quantification in A53T-sfGFP versus A53TΔNAC model (top), and in A53T-sfGFP model seeded with increasing amounts of recombinant PFFs (bottom). (**C**) Heat map of U2OS CRISPR screen gene hits with highest $\log_2$ fold-change differential between 3K-sfGFP, WT-sfGFP and sfGFP indicating genetic modifiers of toxicity specific to 3K expression. (**D**) CRISPR/Cas9 genetic screen hits in selected Gene Ontology (GO) categories

with statistically significant enrichment of gene hits. (**E**) CRISPR screen top hits in GO category "Positive regulation of cytoskeleton organization" (GO: 0051495).

Supplementary Figure 10: Colocalization of CRISPR screen hits with αS within inclusions, related to Figure 8. (**A**) ArpC2 IF in inclusionopathy models. (**B**) PABP IF in inclusionopathy models. (**C**) BioStation CT images of DIV20 pi-Ns with the indicated transgenes transduced with RFP and shRNA lentiviruses. (**D**) Western blot of pi-$N^{3K\text{-}sfGFP\text{-}pB}$ neurons transduced with shRNA-RHOA at different lentivirus multiplicity of infections (MOIs). (**E**) IF of RhoA in seeded and spontaneous inclusionopathy neurons. (**F**) Left: RhoA-p62 PLA in seeded inclusionopathy model. Right: Quantification of αS-sfGFP(+) soma-type inclusions with positive RhoA-p62 PLA signal in spontaneous and seeded inclusion models. (**G**) p62-RhoA PLA signal in ΔNAC and sfGFP control neurons. (**H**) pS129-RhoA PLA signal in ΔNAC and sfGFP control neurons. (**I**) IF for RhoA and BODIPY in familial A53T and sporadic PD postmortem brain. (**J**) Gene set enrichment analysis of MYTH, CRISPR, or combined hits ranked according to association with Lewy body stage based on transcriptome-wide analysis. p-values in these comparisons are two-sided and uncorrected, corresponding to the normalized enrichment score-based test for each gene set independently.

Supplementary Figure 1

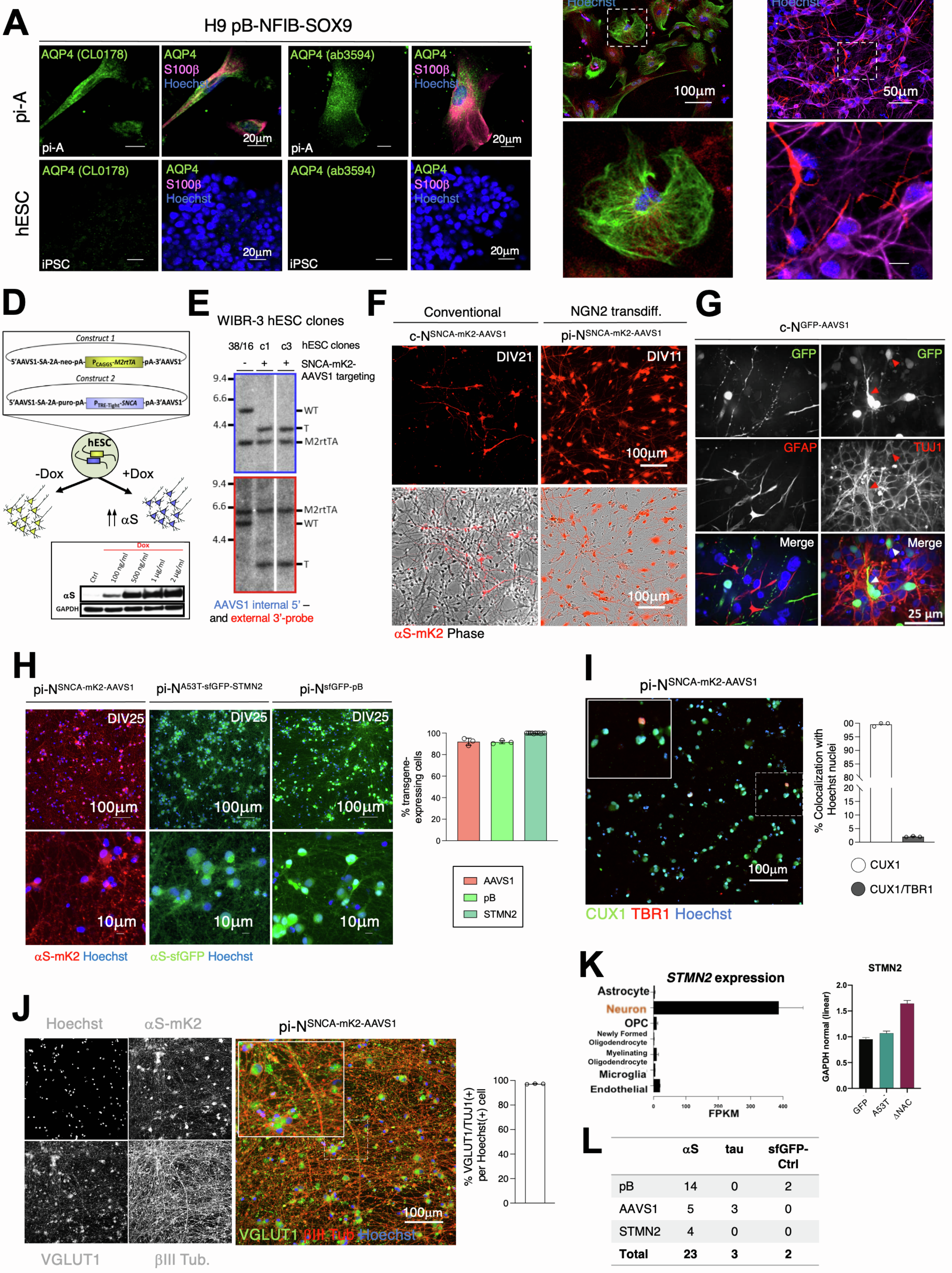

L

	αS	tau	sfGFP-Ctrl
pB	14	0	2
AAVS1	5	3	0
STMN2	4	0	0
**Total**	**23**	**3**	**2**

Supplementary Figure 2

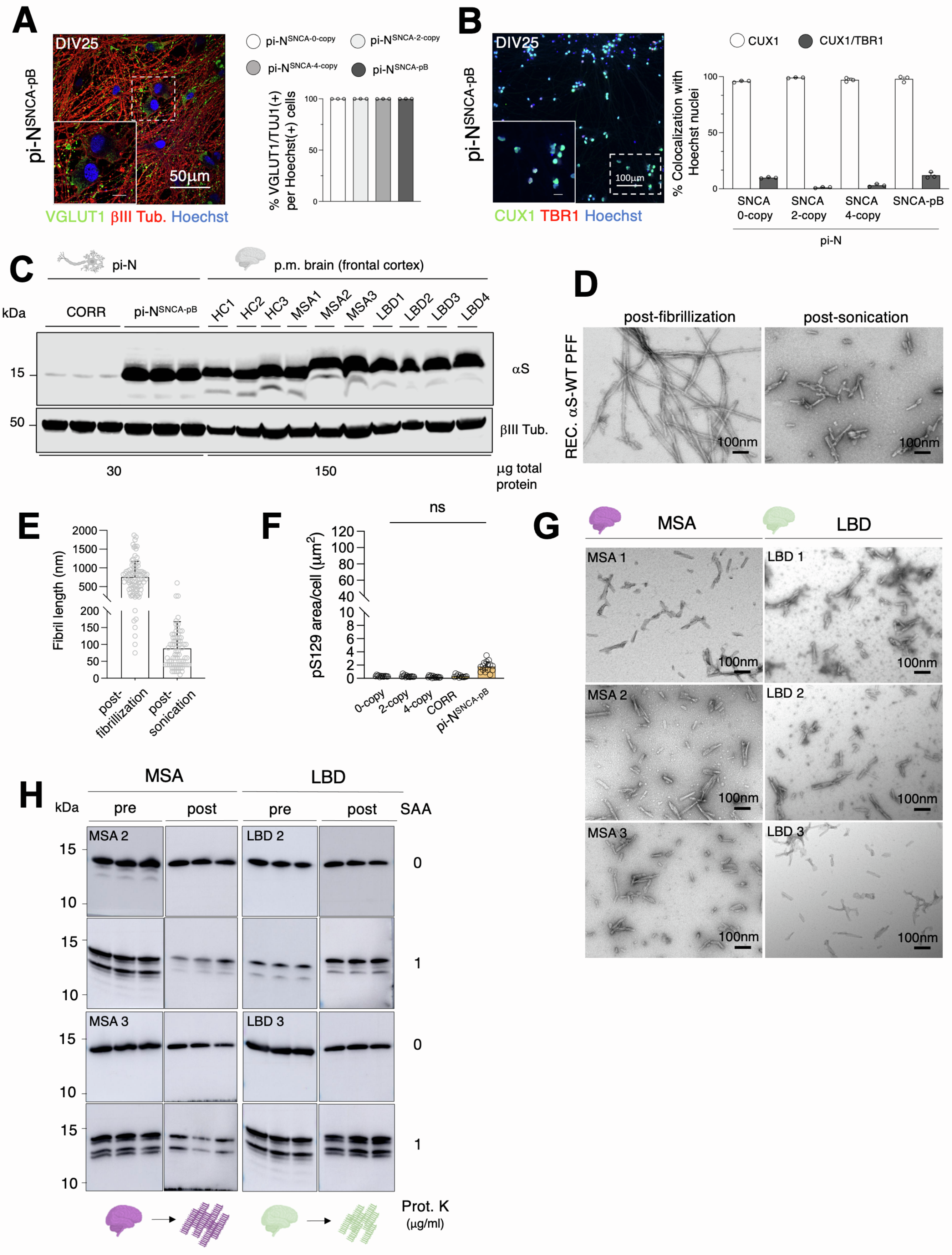

Supplementary Figure 3

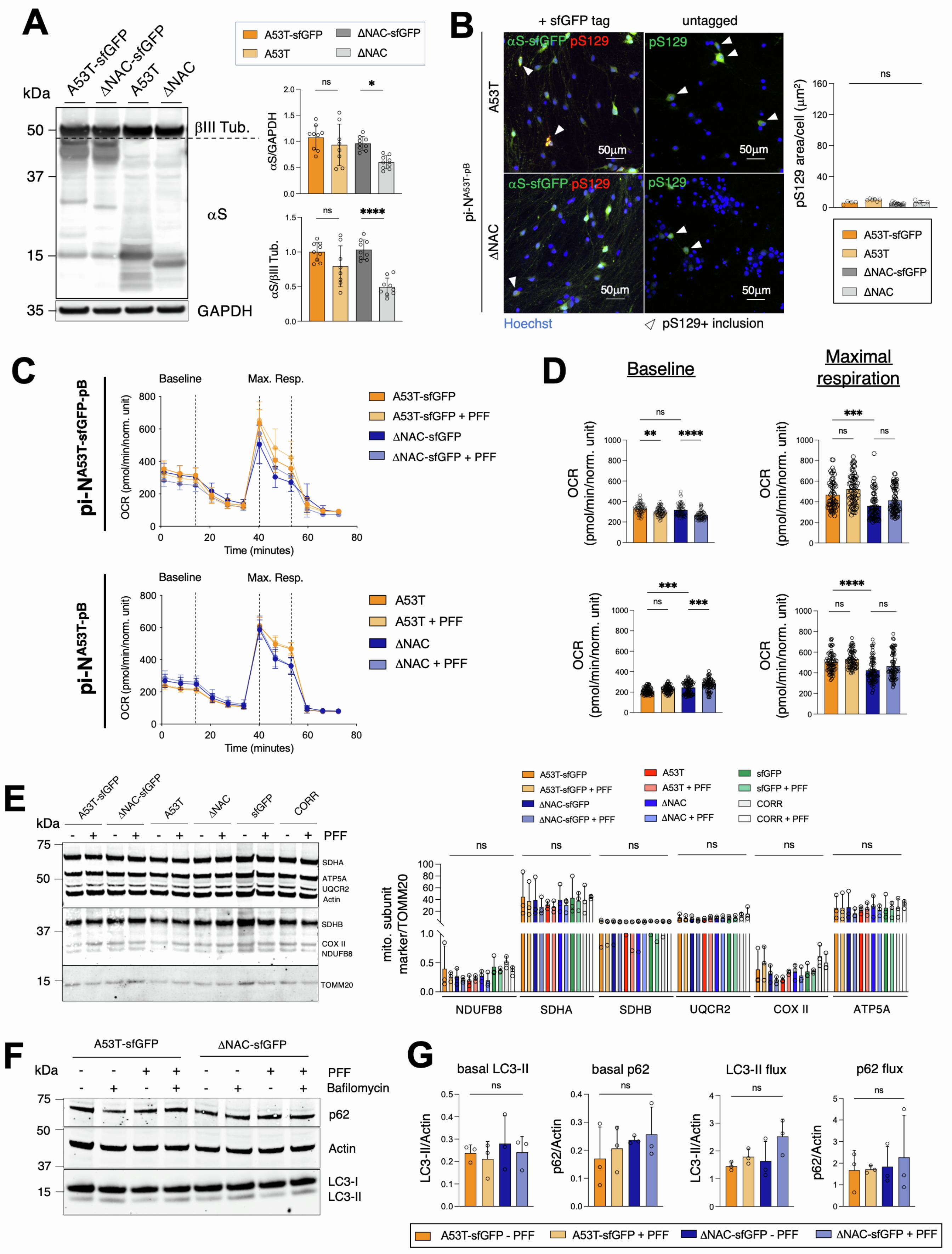

Supplementary Figure 4

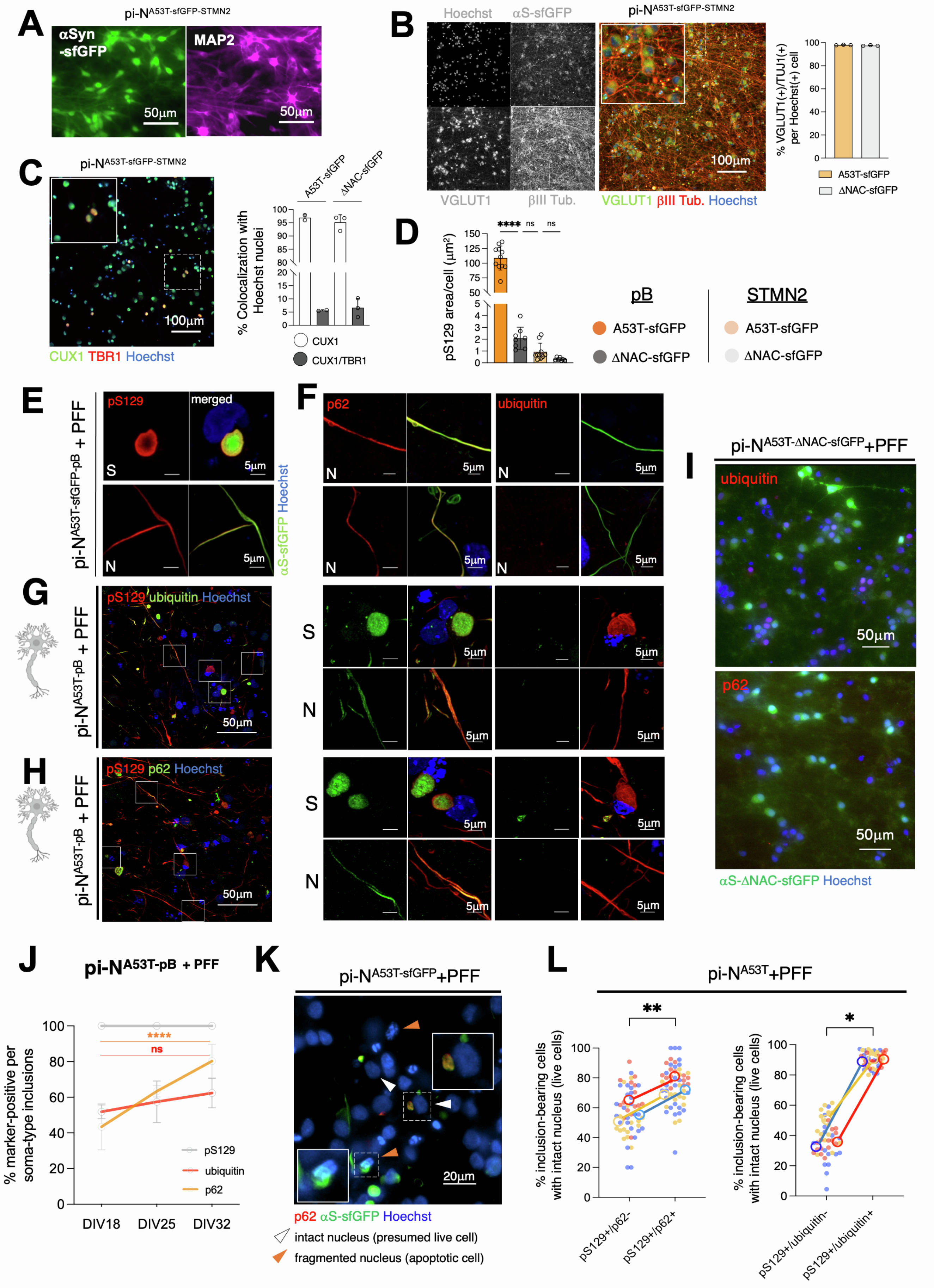

Supplementary Figure 5

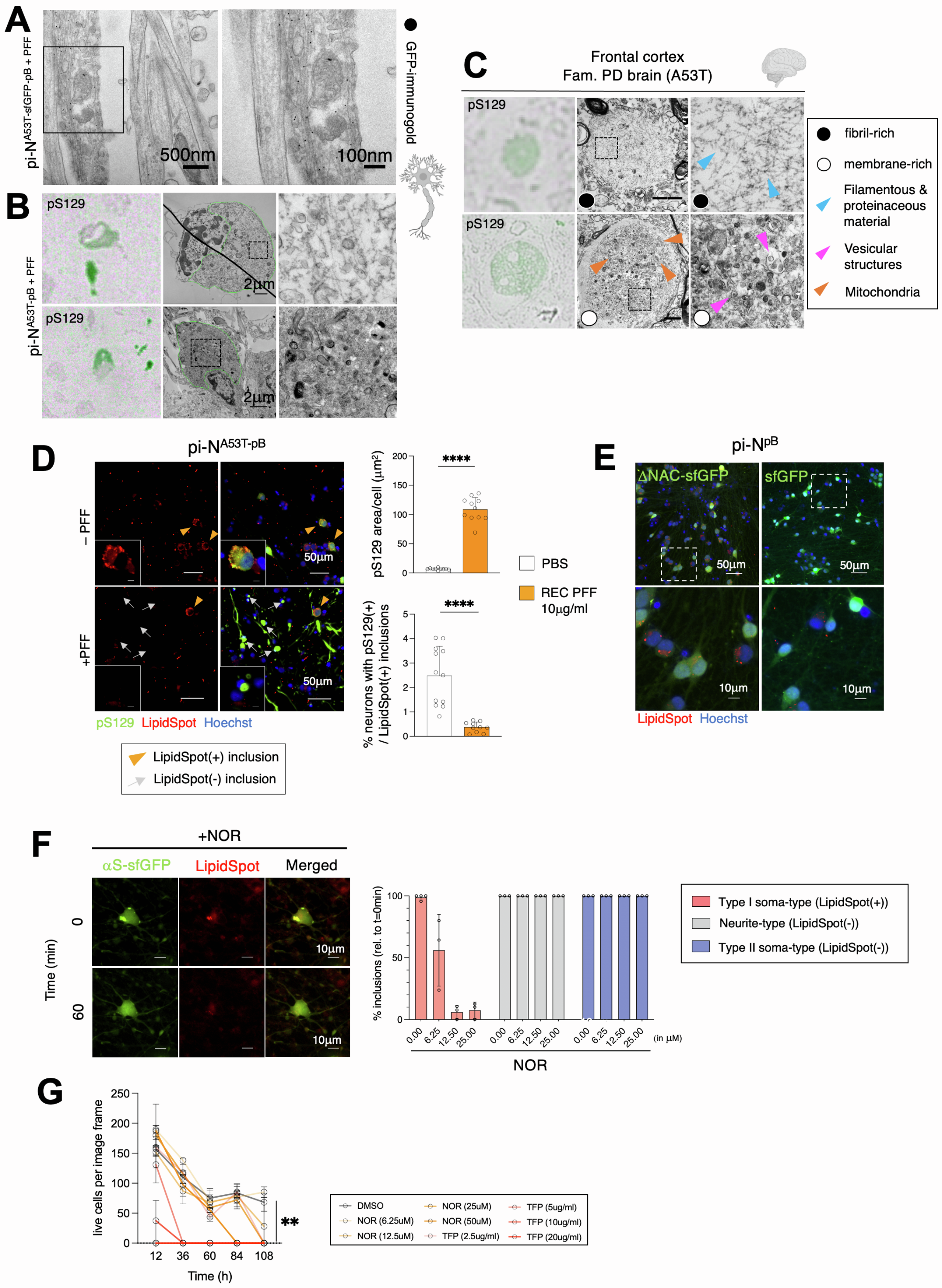

Supplementary Figure 6

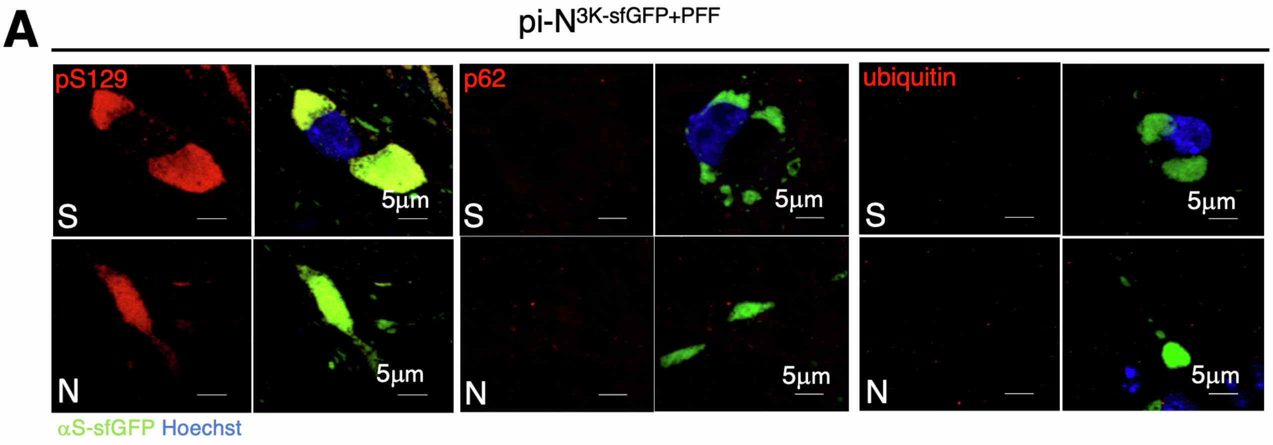

B **SINGLE-CELL INCLUSION TRACKING**

1. Automated detection of neurons and inclusions

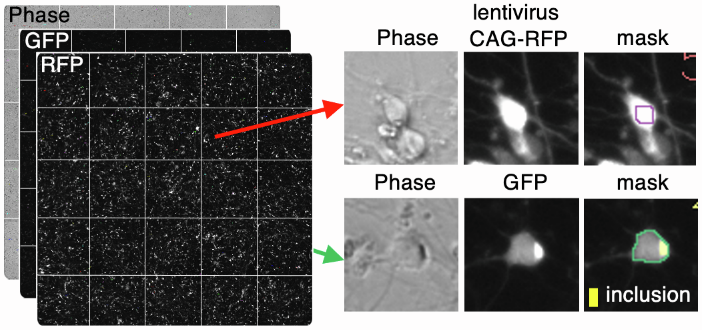

Cell and inclusion detection parameters

	Soma		Neurite
	**Inclusion-**	**Inclusion+**	**Inclusion+**
**Cell body threshold**	-RFP intensity<br>-Cell size	-GFP intensity<br>-Cell size	
**Inclusion threshold**	N/A	-GFP-intensity ratio (inclusion:cell body)<br>- Area ratio (inclusion:cell body)	-GFP-intensity ratio

2. Automated detection of neuron status (live/dead)

Cell death parameters

1. Reduction in cell area
2. Reduction in mean fluorescence intensity (inclusion- RFP; inclusion+ GFP)

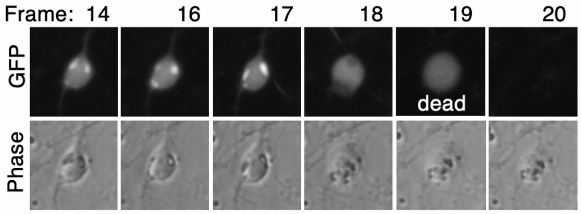

3. Algorithm output

1. Neuron live/dead status (single-cell)
2. Inclusion status (single-cell)
3. Inclusion size (fraction of cell body area)
4. Neurite-type inclusion length (population-avg per well)

C **Automated detection of neuron status (live/dead)**

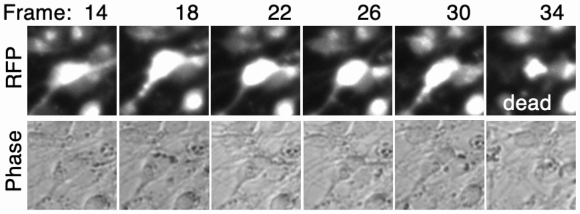

D **Accuracy of automated detection**

		Seeded incl. model	Spontaneous incl. model
Detection	inclusion+	90.6% (87/96)	95.7% (88/92)
	inclusion-	100% (35/35)	95.7% (44/46)
Survival	inclusion+	85.4% (82/96)	82.6% (76/92)
	inclusion-	100% (35/35)	100% (46/46)

E

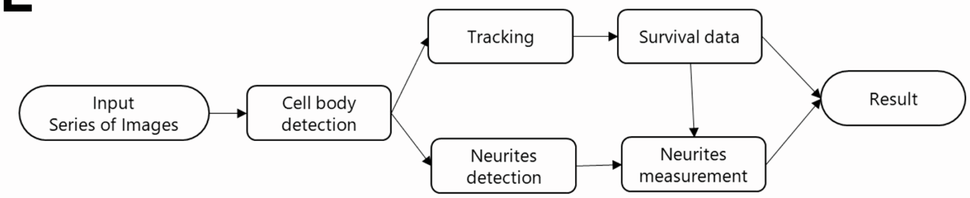

Supplementary Figure 7

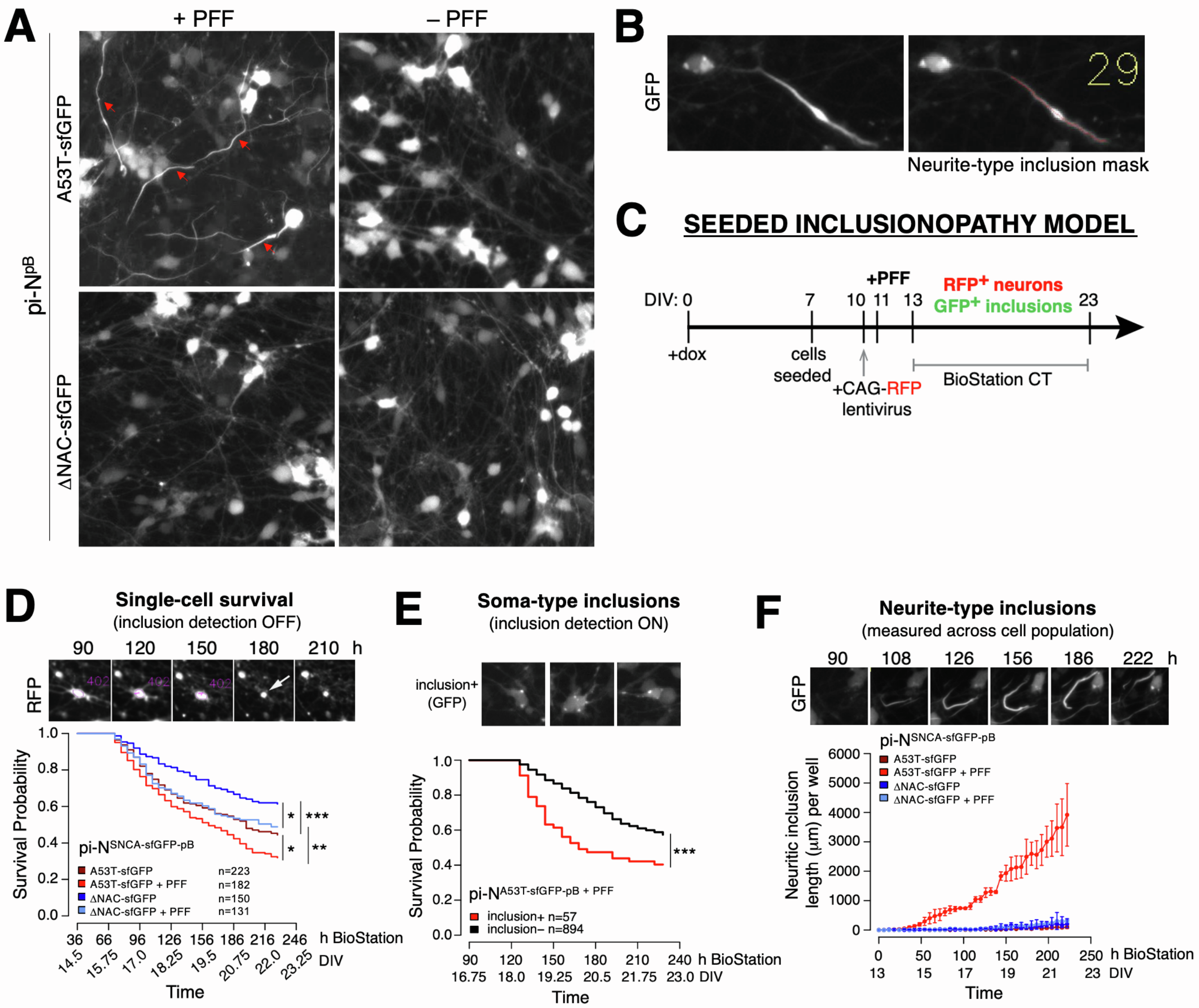

Supplementary Figure 8

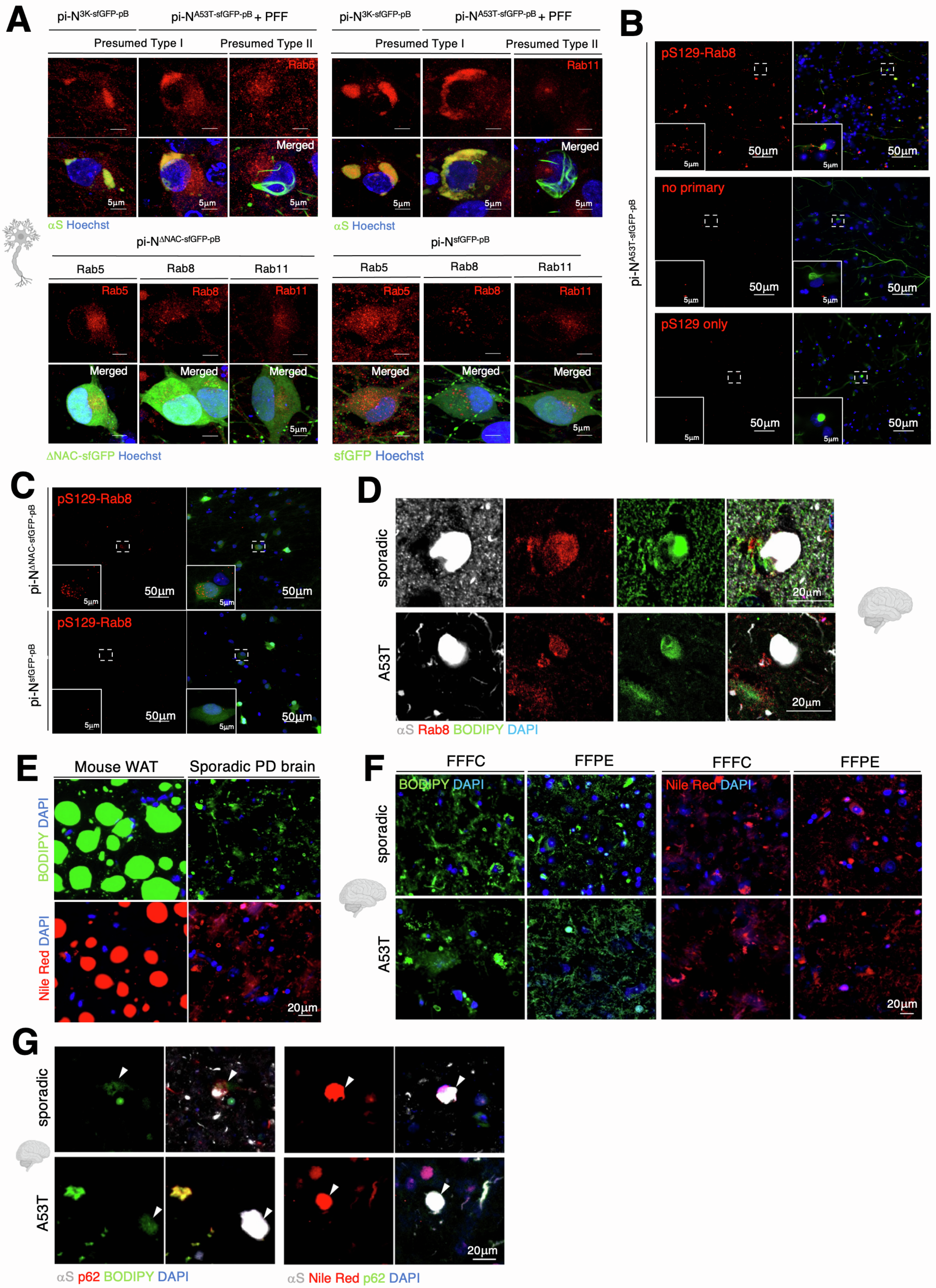

Supplementary Figure 9

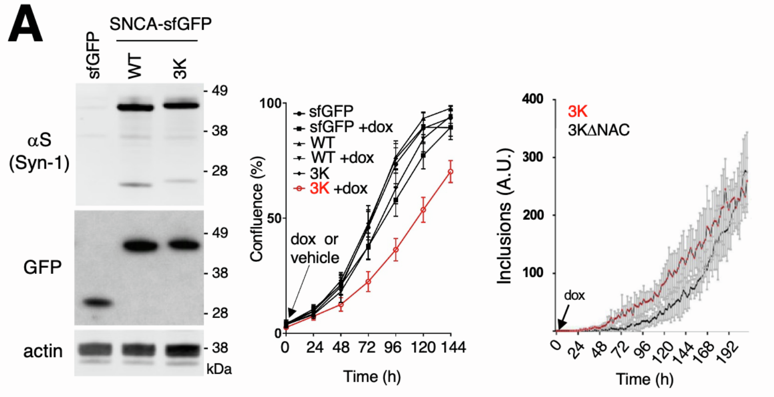

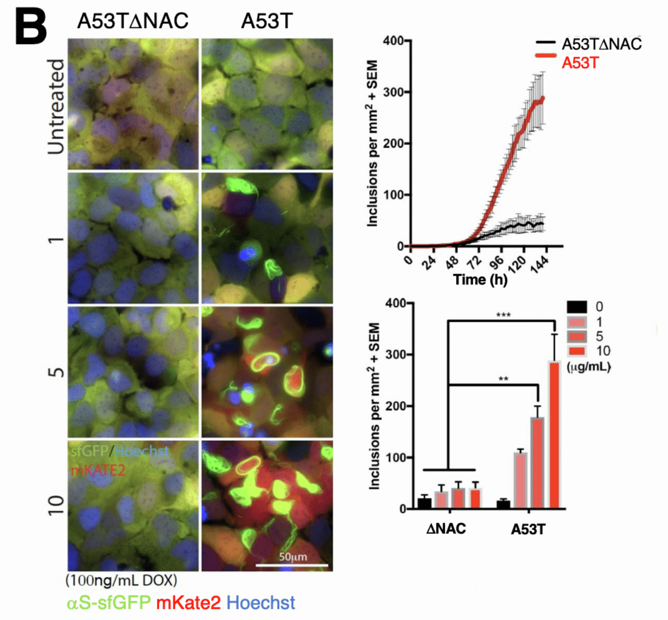

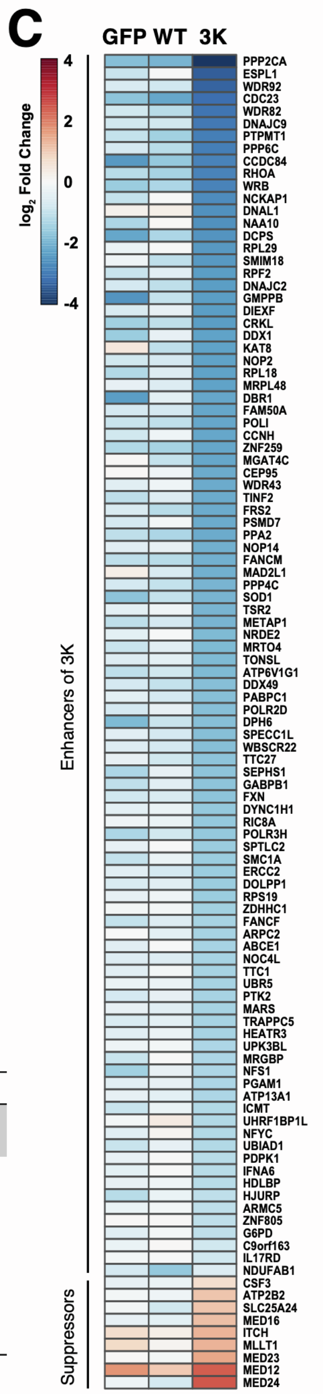

D

U2OS CRISPR/Cas9 genetic screen top hits in selected GO categories

Biological Process	No.	Genes	FDR
Positive regulation of cytoskeleton organization	8	ARPC2, BRK1, CSF3, DYNC1H1, NCKAP1, NRP1, PFN1, RHOA	2.72E-02
RNA metabolic process	38	ADAT3, CCNH, CNOT1, DBR1, DCPS, DDX1, DDX49, ERCC2, EWSR1, FAM50A, GTF2H4, HSD17B10, KAT8, MARS, MED12, MED16, MED23, MED24, MRTO4, NOC4L, NOP14, NOP2, NRDE2, PABPC1, PDE12, POLR2D, POLR3H, PPP2CA, RPF2, RPS19, TSR2, URB1, UTP25, WBSCR22, WDR43, WDR82, YARS2, ZNF259	8.11E-08

E

CRISPR screen top hits in GO category
Positive regulation of cytoskeleton organization (GO:0051495)

Gene	Description
ARPC2	Actin-related protein 2/3 complex subunit 2
BRK1	BRICK1 subunit of SCAR/WAVE actin nucleating complex
CSF3	Member of IL-6 superfamily of cytokines
DYNC1H1	Dynein cytoplasmic 1 heavy chain 1; microtubule-activated ATPase that functions as molecular motor
NCKAP1	Component of WAVE complex that regulates actin filament reorganization via interaction with Arp2/3 complex
NRP1	Cell-surface receptor involved in signaling pathways that control cell migration
PFN1	Profilin 1; involved in monomeric actin conversion to filamentous actin
RHOA	Rho family small GTPase; involved in reorganization of actin cytoskeleton

Supplementary Figure 10

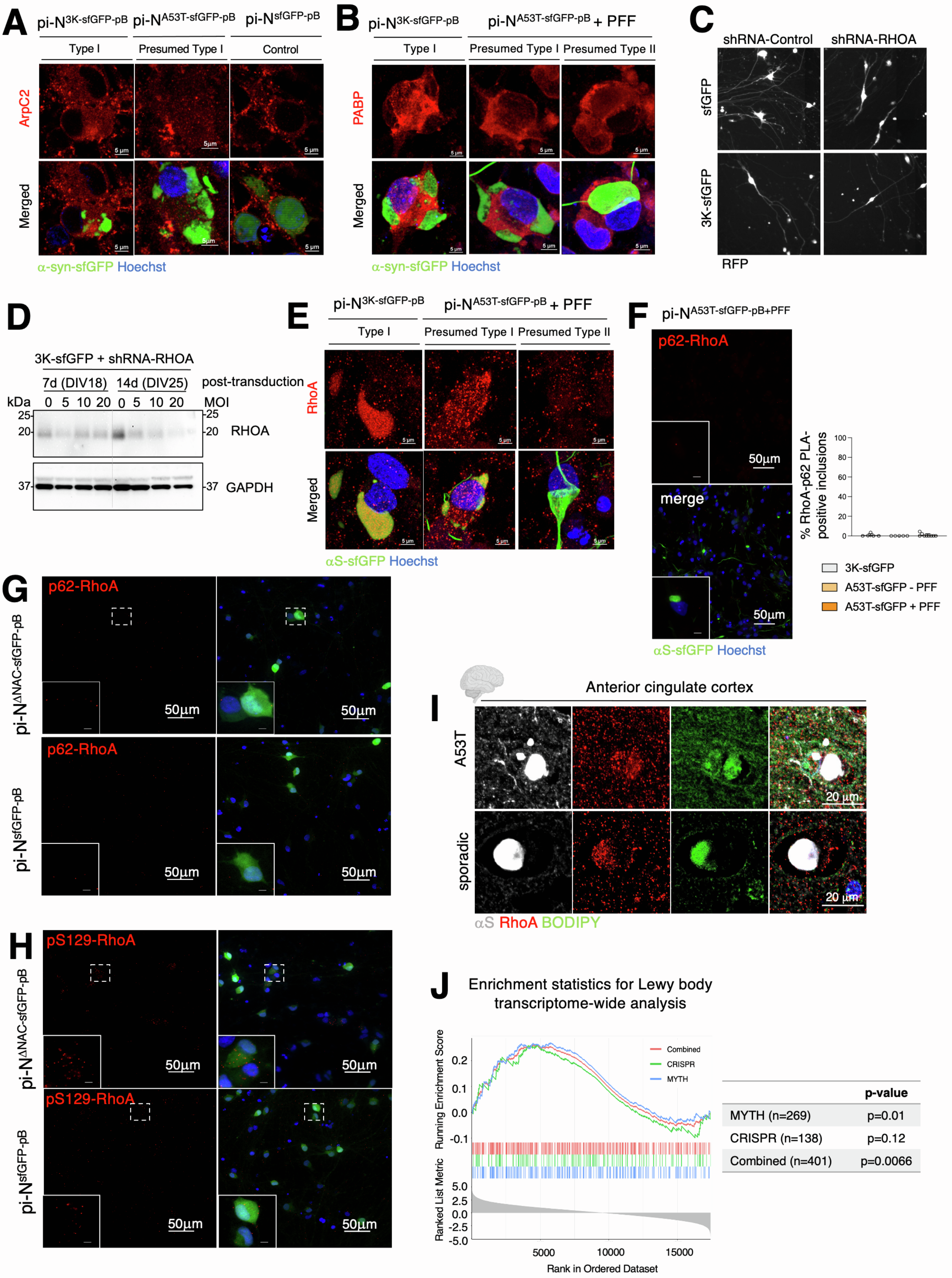

	p-value
MYTH (n=269)	p=0.01
CRISPR (n=138)	p=0.12
Combined (n=401)	p=0.0066
